# Supplementary material for: The Swr1 chromatin-remodeling complex prevents genome instability induced by replication fork progression defects
Source: Nat Commun. 2018 Sep 11;9:3680. doi: 10.1038/s41467-018-06131-2 (PMC6134005; doi:10.1038/s41467-018-06131-2)
Supplement: Supplementary file 1 — Supplementary Information [file 41467_2018_6131_MOESM1_ESM.pdf]

SUPPLEMENTARY INFORMATION FOR

The Swr1 chromatin-remodeling complex prevents genome instability induced by replication fork progression defects

By

Anjana Srivatsan[1], Binzhong Li[1], Barnabas Szakal[2], Dana Branzei[2], Christopher D. Putnam[1,3], and Richard D. Kolodner[1,4,5,6]

From

Ludwig Institute for Cancer Research[1], Departments of Medicine[3] and Cellular and Molecular Medicine[4], Moores-UCSD Cancer Center[5] and Institute of Genomic Medicine[6], University of California School of Medicine, San Diego, 9500 Gilman Drive, La Jolla, CA 92093-0669

And

The FIRC Institute of Molecular Oncology Foundation[2], Via Adamello 16, 20139 Milan, Italy

Address correspondence to:
Richard D. Kolodner
rkolodner@ucsd.edu
(858) 534-7804 (phone)
(858) 822-4479 (fax)

Supplementary Figure 1

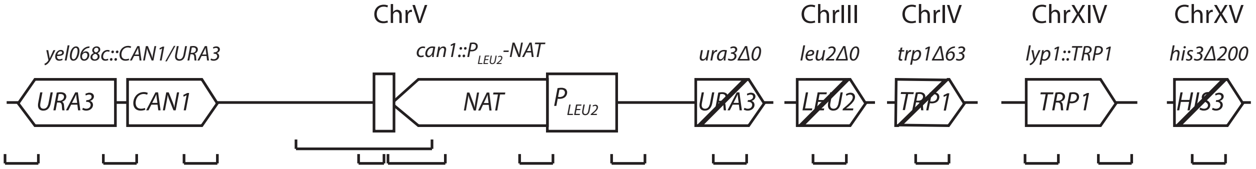

	Junction:	5-A	5-B	5-C	5-DE	5-D	5-E	5-F	5-G	5-H	3-A	4-A	14-A	14-B	15-A
A wild-type	ASY987	262/200	220/211	427/215	139/199	49/131	15/0	137/109	397/117	228/117	208/67	352/121	208/171	71/313	271/107
	ASY988	455/212	522/219	850/222	185/279	119/160	40/127	155/91	666/88	437/145	251/51	548/117	308/203	191/357	545/162
	ASY989	234/142	272/165	421/153	104/18	65/79	17/0	106/79	354/71	302/103	180/32	315/93	221/152	103/301	261/95
	ASYG281	-/-	-/-	-/-	-/-	-/-	-/-	145/59	692/94	381/133	131/27	261/80	143/128	69/258	264/104
	ASYG282	-/-	-/-	-/-	-/-	-/-	-/-	-/-	-/-	197/65	99/37	264/59	148/91	108/196	187/61
	ASYG283	-/-	-/-	-/-	-/-	-/-	-/-	-/-	-/-	228/81	142/34	356/93	160/111	152/307	349/106
	ASYG284	-/-	-/-	-/-	-/-	-/-	-/-	-/-	-/-	241/95	118/32	21/62	163/115	100/256	248/83
	ASYG285	-/-	-/-	-/-	276/220	75/113	37/120	254/77	580/66	316/74	153/25	546/134	277/191	265/348	477/151
	ASYG286	-/-	-/-	-/-	-/-	-/-	-/-	-/-	-/-	389/142	250/80	530/153	316/201	177/367	482/148
	ASYG287	-/-	-/-	-/-	-/-	-/-	-/-	446/158	1108/192	792/208	288/80	628/157	304/215	143/381	469/136
	ASYG288	-/-	-/-	-/-	-/-	-/-	-/-	199/46	700/107	260/76	178/51	319/88	202/143	138/276	295/98
	ASYG289	-/-	-/-	-/-	-/-	-/-	-/-	-/-	-/-	335/140	285/67	534/142	316/166	160/377	585/151
	ASYG291	-/-	-/-	-/-	-/-	-/-	-/-	-/-	-/-	224/78	200/37	325/84	159/131	86/258	256/83
	ASYG292	-/-	-/-	-/-	-/-	-/-	-/-	-/-	-/-	594/135	363/60	918/167	489/232	353/412	867/210
	ASYG293	-/-	-/-	-/-	-/-	-/-	-/-	-/-	-/-	384/124	314/56	529/194	289/196	157/310	469/148
	ASYG294	-/-	-/-	-/-	-/-	-/-	-/-	36/10	423/64	298/111	186/34	361/90	220/144	110/287	285/109
	ASYG470	-/-	-/-	-/-	161/225	88/146	33/92	124/79	454/78	347/115	260/69	394/47	309/169	123/307	355/115
	ASYG471	606/218	739/235	1005/231	264/265	127/157	66/132	213/90	777/100	531/119	291/61	691/127	379/234	318/374	687/162
	ASYG472	-/-	-/-	-/-	-/-	-/-	-/-	-/-	-/-	384/128	328/62	571/115	316/194	228/349	524/118
	ASYG473	-/-	-/-	-/-	114/2	92/141	23/0	107/78	483/77	278/101	260/49	380/94	206/161	99/311	331/117
	ASYG474	-/-	-/-	-/-	146/220	47/162	20/0	145/83	360/90	292/122	251/72	385/119	242/170	86/327	325/88
	ASYG475	-/-	-/-	-/-	-/-	-/-	-/-	-/-	-/-	266/101	232/56	279/97	226/142	73/292	257/101
	ASYG476	-/-	-/-	-/-	76/189	76/128	25/0	86/63	315/53	270/100	204/41	284/86	166/126	82/269	243/102
B *swr1*	ASY1118	657/268	899/277	1425/309	328/405	103/204	100/226	330/167	945/120	460/157	343/74	671/161	266/317	260/205	760/211
	ASY1119	578/229	591/246	821/216	313/294	115/179	54/136	251/102	711/96	485/145	277/70	645/153	393/207	262/380	607/142
	ASY1120	380/173	412/173	607/171	130/221	94/161	37/110	121/82	469/65	304/97	244/50	401/104	284/151	141/346	331/107
	ASYG308	-/-	-/-	-/-	-/-	-/-	-/-	370/63	1253/165	907/233	261/63	733/164	369/219	316/423	539/164
	ASYG309	-/-	-/-	-/-	-/-	-/-	-/-	-/-	-/-	203/80	77/45	344/93	204/131	138/269	316/96
	ASYG310	-/-	-/-	-/-	-/-	-/-	-/-	-/-	-/-	282/103	142/29	353/91	218/159	149/331	321/132
	ASYG311	-/-	-/-	-/-	-/-	-/-	-/-	-/-	-/-	318/103	207/60	399/129	279/168	131/310	343/124
	ASYG312	-/-	-/-	-/-	-/-	-/-	-/-	-/-	-/-	231/82	154/31	302/89	148/138	96/272	300/112
	ASYG313	-/-	-/-	-/-	142/221	81/83	44/123	143/85	456/73	294/109	181/30	420/90	225/152	131/305	333/103
	ASYG314	-/-	-/-	559/232	235/270	67/183	18/0	217/111	476/125	420/159	327/94	502/143	355/215	105/347	390/135
	ASYG315	-/-	-/-	-/-	-/-	-/-	-/-	-/-	-/-	284/117	288/67	385/110	231/162	83/313	322/117
	ASYG316	-/-	-/-	-/-	-/-	-/-	-/-	-/-	-/-	198/84	138/35	276/63	132/122	75/215	190/68
	ASYG317	-/-	-/-	-/-	-/-	-/-	-/-	162/42	896/107	359/133	245/55	506/125	298/182	162/349	397/109
	ASYG318	-/-	-/-	-/-	126/235	96/144	31/113	147/69	460/61	311/114	240/51	451/105	245/186	170/306	427/106
	ASYG484	-/-	-/-	-/-	-/-	-/-	-/-	-/-	-/-	369/144	223/80	449/116	310/224	196/332	442/131
	ASYG485	-/-	-/-	-/-	-/-	-/-	-/-	371/45	1216/114	1057/229	339/64	719/136	476/226	293/363	523/132
	ASYG486	-/-	-/-	-/-	-/-	-/-	-/-	176/74	472/115	360/136	345/72	517/132	332/202	103/353	396/138
	ASYG487	-/-	-/-	-/-	-/-	-/-	-/-	-/-	-/-	400/151	411/73	637/150	354/4	144/390	415/153
	ASYG488	-/-	-/-	-/-	-/-	-/-	-/-	-/-	-/-	267/143	225/75	365/145	254/225	99/533	257/105
	ASYG489	-/-	-/-	-/-	-/-	-/-	-/-	-/-	521/93	353/119	310/52	599/114	322/189	176/368	449/135
	ASYG490	-/-	-/-	-/-	-/-	-/-	-/-	161/126	1072/140	689/204	299/56	489/117	273/201	184/366	462/117

Supplementary Figure 1

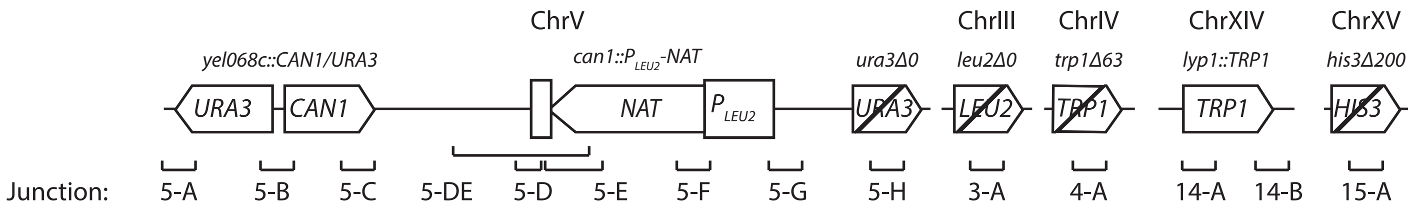

C

mrc1

Strain	5-A	5-B	5-C	5-DE	5-D	5-E	5-F	5-G	5-H	3-A	4-A	14-A	14-B	15-A
ASY1112	411/197	433/210	545/202	374/303	97/151	72/160	317/137	49/131	262/105	201/61	551/135	273/183	191/356	426/131
ASY1113	483/260	610/260	705/218	493/365	116/181	142/203	459/182	850/222	281/132	243/67	669/186	252/196	206/407	539/180
ASY1114	279/153	320/166	385/140	234/257	54/106	50/155	246/125	421/153	202/86	172/42	391/86	210/124	99/301	282/100
ASYG295	-/-	-/-	-/-	-/-	-/-	-/-	-/-	-/-	537/217	335/65	563/132	327/190	231/368	471/127
ASYG296	-/-	-/-	-/-	-/-	-/-	-/-	-/-	-/-	579/180	216/52	547/106	333/206	188/330	383/110
ASYG297	-/-	-/-	-/-	346/231	93/107	64/136	302/87	482/58	292/105	190/36	549/121	268/181	179/321	437/123
ASYG298	-/-	-/-	-/-	400/311	84/151	66/169	344/96	527/57	318/111	200/42	613/121	329/188	247/339	473/136
ASYG299	-/-	-/-	-/-	249/265	97/146	53/129	230/95	504/63	294/102	239/50	493/103	293/172	164/332	414/110
ASYG300	-/-	-/-	-/-	-/-	-/-	-/-	294/54	739/96	227/106	175/51	432/131	355/196	190/338	370/111
ASYG301	-/-	-/-	-/-	-/-	-/-	-/-	-/-	-/-	64/90	87/22	97/62	22/60	24/165	178/81
ASYG302	-/-	-/-	-/-	180/189	489/371	132/193	431/150	826/87	263/89	196/46	525/98	297/146	194/310	403/121
ASYG303	-/-	-/-	-/-	-/-	-/-	-/-	-/-	-/-	221/71	139/24	408/77	240/142	146/293	323/111
ASYG306	-/-	-/-	-/-	-/-	-/-	-/-	127/77	280/78	217/88	182/69	359/115	216/173	114/291	263/105
ASYG307	-/-	-/-	-/-	-/-	-/-	-/-	-/-	-/-	490/159	192/55	437/102	263/135	150/294	334/110
ASYG477	-/-	-/-	-/-	-/-	-/-	-/-	-/-	36/45	199/127	203/91	419/146	268/193	130/334	305/129
ASYG478	-/-	-/-	-/-	-/-	-/-	-/-	-/-	59/18	165/79	123/39	301/71	160/122	105/261	259/73
ASYG479	-/-	-/-	-/-	-/-	-/-	-/-	-/-	-/-	354/119	154/27	381/80	225/127	116/259	303/88
ASYG480	-/-	-/-	-/-	423/268	124/122	66/152	431/112	438/52	368/120	180/32	516/126	206/141	147/261	347/101
ASYG481	-/-	-/-	-/-	-/-	-/-	-/-	295/34	641/94	425/142	151/28	370/108	291/139	111/281	241/87
ASYG482	-/-	-/-	-/-	-/-	-/-	-/-	158/34	530/69	306/101	132/23	286/70	175/121	103/258	258/77
ASYG483	-/-	-/-	-/-	-/-	-/-	-/-	413/41	678/87	510/152	195/37	552/121	284/140	163/291	360/88

D

mrc1 swr1

Strain	5-A	5-B	5-C	5-DE	5-D	5-E	5-F	5-G	5-H	3-A	4-A	14-A	14-B	15-A
ASY1202	445/151	479/173	715/180	389/241	90/105	62/142	285/95	505/51	267/90	132/25	433/104	209/159	194/296	449/127
ASY1203	442/176	456/240	753/213	304/273	64/139	46/154	255/120	516/72	298/105	140/31	510/323	252/171	177/368	367/142
ASY1204	579/187	705/199	923/202	493/298	89/149	87/168	394/122	661/61	377/99	176/40	672/141	283/191	279/380	660/161
ASYG320	-/-	-/-	-/-	-/-	-/-	-/-	-/-	-/-	281/108	222/39	497/137	246/161	122/302	350/121
ASYG321	-/-	-/-	-/-	-/-	-/-	-/-	-/-	-/-	353/100	171/62	550/136	298/182	158/340	389/135
ASYG322	-/-	-/-	-/-	300/222	118/119	56/122	269/86	505/40	439/109	157/41	637/123	340/164	292/319	492/139
ASYG323	-/-	-/-	-/-	-/-	-/-	-/-	23/45	481/54	381/102	159/34	565/113	299/194	283/337	489/117
ASYG324	-/-	-/-	-/-	250/233	64/131	27/102	185/69	501/62	336/95	126/40	520/116	325/335	253/190	408/123
ASYG325	-/-	-/-	-/-	-/-	-/-	-/-	-/-	-/-	370/113	98/25	309/70	179/113	129/241	239/93
ASYG326	-/-	-/-	-/-	-/-	-/-	-/-	-/-	-/-	281/92	118/28	456/89	249/156	207/331	378/99
ASYG327	527/166	-/-	-/-	341/298	130/178	45/144	303/92	633/80	445/150	302/54	645/113	366/215	290/407	586/169
ASYG328	-/-	-/-	-/-	-/-	-/-	-/-	-/-	-/-	248/93	188/38	438/110	245/174	164/335	322/114
ASYG329	-/-	-/-	-/-	-/-	-/-	-/-	-/-	-/-	383/146	227/53	703/184	402/235	218/411	661/188
ASYG330	-/-	-/-	-/-	-/-	-/-	-/-	-/-	-/-	414/181	346/70	626/150	414/235	173/372	454/157
ASYG331	-/-	-/-	373/127	120/160	78/106	17/0	103/57	295/65	410/139	185/34	451/133	247/140	121/306	385/144
ASYG332	-/-	-/-	-/-	-/-	-/-	-/-	-/-	-/-	319/115	278/59	466/139	354/205	151/356	370/122
ASYG333	610/246	-/-	821/260	507/368	118/198	74/175	440/114	614/90	486/161	290/70	705/169	492/253	286/437	584/164
ASYG547	-/-	-/-	593/190	425/340	126/184	65/165	440/128	544/83	531/122	280/56	800/148	460/215	278/396	560/153
ASYG548	-/-	-/-	-/-	193/278	113/153	62/137	173/103	520/87	346/118	287/42	506/130	291/183	118/332	400/111
ASYG549	281/133	439/71	421/154	175/186	68/126	23/0	152/68	385/66	276/90	205/36	367/99	256/155	131/311	314/104
ASYG550	-/-	-/-	-/-	-/-	-/-	-/-	-/-	-/-	331/109	486/101	416/113	252/167	103/315	319/113
ASYG551	-/-	-/-	-/-	-/-	-/-	-/-	-/-	-/-	334/115	357/44	545/124	255/184	141/341	462/167
ASYG554	-/-	-/-	567/171	314/280	109/147	72/140	336/107	529/87	389/119	296/53	622/140	301/200	208/371	498/125
ASYG555	-/-	-/-	-/-	-/-	-/-	-/-	-/-	-/-	327/79	152/32	377/90	231/107	151/270	330/86
ASYG319	212/135	323/86	406/117	105/172	66/91	8/0	76/50	295/54	226/77	166/51	289/80	170/129	102/253	244/89

Supplementary Figure 1

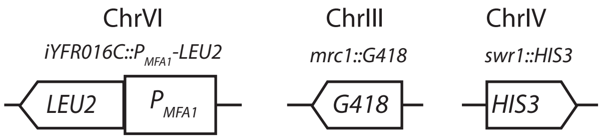

E

Junction:	6-A	6-B	6-C	3-B	3-C	4-B	4-C
**wild-type**							
ASY987	320/210	406/119	258/330	n.a.	n.a.	n.a.	n.a.
ASY988	512/183	764/134	595/315	n.a.	n.a.	n.a.	n.a.
ASY989	275/156	392/96	252/241	n.a.	n.a.	n.a.	n.a.
ASYG281	279/144	377/98	305/284	n.a.	n.a.	n.a.	n.a.
ASYG282	208/90	298/69	264/191	n.a.	n.a.	n.a.	n.a.
ASYG283	432/168	554/116	515/312	n.a.	n.a.	n.a.	n.a.
ASYG284	245/136	364/95	273/251	n.a.	n.a.	n.a.	n.a.
ASYG285	556/194	635/118	534/271	n.a.	n.a.	n.a.	n.a.
ASYG286	486/249	607/153	400/334	n.a.	n.a.	n.a.	n.a.
ASYG287	433/222	565/132	390/319	n.a.	n.a.	n.a.	n.a.
ASYG288	362/136	384/89	244/238	n.a.	n.a.	n.a.	n.a.
ASYG289	470/210	617/147	521/308	n.a.	n.a.	n.a.	n.a.
ASYG291	319/148	357/97	287/246	n.a.	n.a.	n.a.	n.a.
ASYG292	835/259	994/163	798/350	n.a.	n.a.	n.a.	n.a.
ASYG293	479/173	653/149	613/306	n.a.	n.a.	n.a.	n.a.
ASYG294	339/178	489/118	445/278	n.a.	n.a.	n.a.	n.a.
ASYG470	347/168	520/121	411/299	n.a.	n.a.	n.a.	n.a.
ASYG471	747/247	906/163	690/325	n.a.	n.a.	n.a.	n.a.
ASYG472	523/201	698/137	449/306	n.a.	n.a.	n.a.	n.a.
ASYG473	402/172	503/138	453/307	n.a.	n.a.	n.a.	n.a.
ASYG474	289/158	437/130	233/282	n.a.	n.a.	n.a.	n.a.
ASYG475	273/175	409/114	274/274	n.a.	n.a.	n.a.	n.a.
ASYG476	279/147	424/113	321/284	n.a.	n.a.	n.a.	n.a.

H

swr1							
ASY1118	803/288	1066/209	971/414	n.a.	n.a.	340/192	407/120
ASY1119	678/237	797/154	566/318	n.a.	n.a.	331/178	468/154
ASY1120	378/193	493/127	372/269	n.a.	n.a.	230/136	258/109
ASYG308	637/236	732/164	563/343	n.a.	n.a.	387/192	385/173
ASYG309	300/156	423/106	426/283	n.a.	n.a.	183/119	184/77
ASYG310	440/206	574/119	540/319	n.a.	n.a.	210/128	220/75
ASYG311	340/192	484/140	368/309	n.a.	n.a.	198/123	225/97
ASYG312	337/189	504/127	491/331	n.a.	n.a.	120/77	138/52
ASYG313	352/190	542/135	410/282	n.a.	n.a.	224/129	259/92
ASYG314	410/233	550/160	332/329	n.a.	n.a.	283/180	340/174
ASYG315	323/146	478/118	338/2	n.a.	n.a.	212/122	299/132
ASYG316	277/138	404/99	404/275	n.a.	n.a.	151/98	173/64
ASYG317	410/199	562/137	490/299	n.a.	n.a.	272/110	332/108
ASYG318	446/190	619/129	472/297	n.a.	n.a.	556/213	582/172
ASYG484	471/198	547/138	430/312	n.a.	n.a.	305/125	354/115
ASYG485	632/217	756/142	500/292	n.a.	n.a.	382/149	541/170
ASYG486	424/209	529/149	372/319	n.a.	n.a.	257/159	354/170
ASYG487	518/267	696/169	496/350	n.a.	n.a.	323/178	356/192
ASYG488	335/0	427/166	311/515	n.a.	n.a.	208/194	229/122
ASYG489	513/199	646/148	451/295	n.a.	n.a.	347/148	442/136
ASYG490	498/199	628/164	521/331	n.a.	n.a.	339/143	354/148

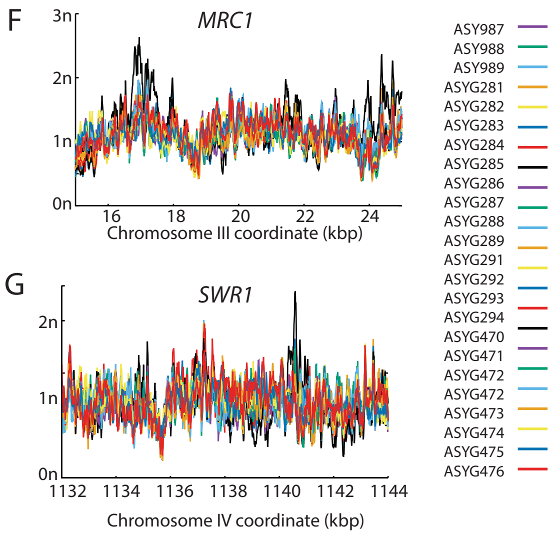

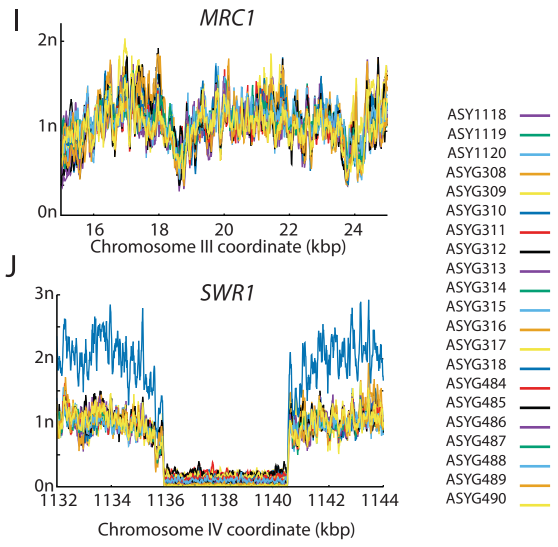

Supplementary Figure 1

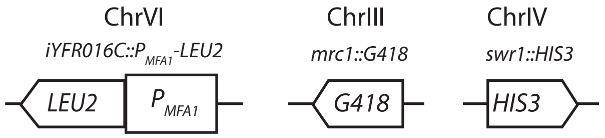

K

	Junction:	6-A	6-B	6-C	3-B	3-C	4-B	4-C
mrc1	ASY1112	445/191	529/133	475/352	357/84	879/182	n.a.	n.a.
	ASY1113	571/265	733/173	508/365	471/160	1137/215	n.a.	n.a.
	ASY1114	375/161	375/104	337/277	250/79	631/150	n.a.	n.a.
	ASYG295	520/220	630/147	511/344	500/119	952/192	n.a.	n.a.
	ASYG296	537/184	521/119	490/331	372/85	899/155	n.a.	n.a.
	ASYG297	494/198	612/104	543/276	371/66	852/157	n.a.	n.a.
	ASYG298	485/190	586/11	550/312	431/78	953/178	n.a.	n.a.
	ASYG299	431/193	555/143	471/318	360/87	833/171	n.a.	n.a.
	ASYG300	411/184	484/128	413/321	339/95	632/138	n.a.	n.a.
	ASYG301	299/170	277/119	370/255	140/27	713/130	n.a.	n.a.
	ASYG302	423/205	529/119	548/305	309/11	954/168	n.a.	n.a.
	ASYG303	385/157	441/101	410/287	246/98	819/120	n.a.	n.a.
	ASYG306	261/164	376/116	265/302	241/90	502/105	n.a.	n.a.
	ASYG307	299/164	425/121	310/260	302/90	496/117	n.a.	n.a.
	ASYG477	347/216	448/142	346/309	284/66	594/119	n.a.	n.a.
	ASYG478	236/108	309/74	251/252	230/43	402/89	n.a.	n.a.
	ASYG479	333/134	388/98	385/251	268/56	694/128	n.a.	n.a.
	ASYG480	343/136	386/72	486/255	334/52	883/151	n.a.	n.a.
	ASYG481	307/115	401/93	303/249	297/64	489/111	n.a.	n.a.
	ASYG482	297/127	362/85	333/236	223/50	503/114	n.a.	n.a.
	ASYG483	397/148	501/98	333/236	389/77	813/147	n.a.	n.a.

N

		6-A	6-B	6-C	3-B	3-C	4-B	4-C
mrc1 swr1	ASY1202	536/194	536/194	541/298	352/55	829/138	275/153	309/77
	ASY1203	523/183	523/183	519/323	345/82	962/178	279/168	321/93
	ASY1204	676/223	676/223	722/320	1409/204	414/77	502/177	398/101
	ASYG320	387/192	387/192	395/307	360/88	603/148	232/148	297/122
	ASYG321	454/199	454/199	479/304	380/95	807/192	275/170	319/104
	ASYG322	482/169	482/169	479/283	551/94	632/131	332/136	417/113
	ASYG323	479/189	479/189	506/252	497/103	764/165	283/152	358/109
	ASYG324	579/226	579/226	518/323	384/97	698/148	291/149	359/100
	ASYG325	324/128	324/128	292/221	457/92	704/167	148/91	227/75
	ASYG326	427/148	427/148	384/257	282/59	496/116	229/125	308/87
	ASYG327	673/232	673/232	583/322	573/108	822/175	375/157	498/129
	ASYG328	428/201	428/201	406/307	607/158	333/88	213/136	273/109
	ASYG329	708/290	708/290	678/387	484/113	1036/220	361/194	467/165
	ASYG330	637/256	637/256	475/364	456/121	835/197	326/155	429/162
	ASYG331	405/156	405/156	336/267	320/80	450/98	289/153	425/160
	ASYG332	441/173	441/173	369/310	405/119	621/157	252/131	346/156
	ASYG333	704/276	704/276	660/383	539/140	1072/229	704/297	803/254
	ASYG547	563/190	563/190	584/318	659/138	933/177	348/170	408/156
	ASYG548	514/184	514/184	471/324	372/86	831/167	236/127	349/138
	ASYG549	330/146	330/146	295/263	359/86	402/97	178/114	269/107
	ASYG550	380/167	380/167	345/299	644/149	891/221	226/127	292/115
	ASYG551	528/224	528/224	561/345	357/91	816/143	273/139	363/126
	ASYG554	585/182	585/182	508/287	491/98	863/168	312/155	413/147
	ASYG555	373/139	373/139	332/255	251/65	643/160	222/105	271/93
	ASYG319	273/143	273/143	278/275	271/68	426/98	156/100	187/73

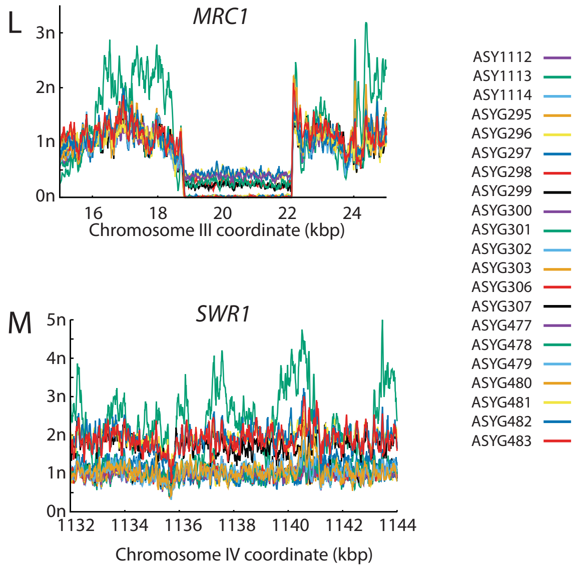

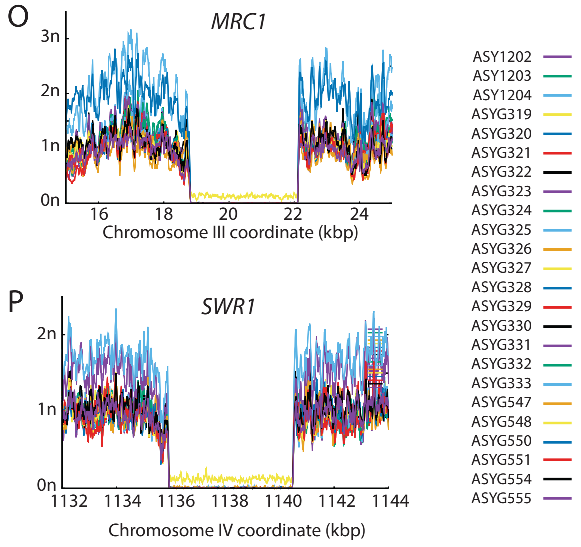

Supplementary Figure 1. Identification of the engineered chromosomal features in uGCR assay strains by WGS. A-E, H, K, N. For each junction, the evidence in the paired-end sequencing data is reported. The number preceding the slash is the number of junction-defining read pairs (those for which one read maps to one side of the junction and the other read maps to the other side of the junction). The number following the slash is the number of junction-sequencing reads (those that can be aligned to derive the sequence of the junction). "-/-" indicates a junction that could have been observed but was not observed, which is typically due to a GCR-related deletion. Note that some sequences are short enough that some read pairs span multiple junctions, e.g. junction 5-DE contains read pairs that span both junctions 5-D and 5-E. **F, G, I, J, L-P.** Read-depth analysis of the regions including *MRC1* and *SWR1*, indicating that the expected deletions were observed for strains of each relevant genotype. In some cases, GCR-associated chromosomal rearrangements increase the copy number of sequences adjacent to the deletion to 2n.

Supplementary Figure 2

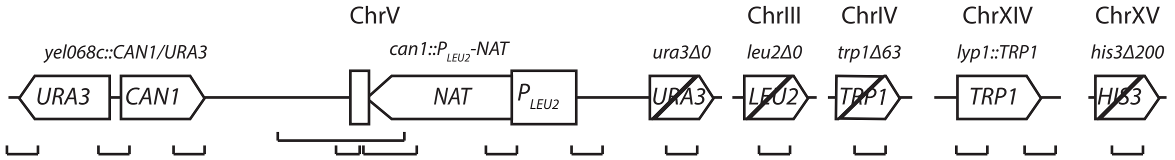

		Junction:	5-A	5-B	5-C	5-DE	5-D	5-E	5-F	5-G	5-H	3-A	4-A	14-A	14-B	15-A
A	*swr1*	ASY566	12/20	21/21	27/14	9/5	12/4	12/7	13/7	14/9	24/9	25/2	32/11	11/30	16/12	20/2
		ASYG176	-/-	-/-	-/-	-/-	-/-	-/-	4/0	9/2	38/9	16/12	21/6	6/27	14/8	11/3
		ASYG178	-/-	-/-	-/-	-/-	-/-	-/-	-/-	22/11	55/25	28/5	19/9	4/0	12/15	14/3
		ASYG179	-/-	-/-	-/-	-/-	-/-	-/-	-/-	-/-	26/8	33/11	24/9	8/17	21/6	12/6
		ASYG180	-/-	-/-	-/-	-/-	-/-	-/-	-/-	-/-	21/5	16/10	26/8	2/0	15/10	16/4
		ASYG261	-/-	-/-	-/-	-/-	-/-	-/-	-/-	-/-	19/7	12/4	15/5	7/20	6/7	20/6
		ASYG262	-/-	-/-	-/-	-/-	-/-	-/-	-/-	-/-	35/15	24/10	16/6	5/0	14/21	15/7
		ASYG271	-/-	-/-	-/-	-/-	-/-	-/-	-/-	-/-	20/5	26/10	16/7	3/0	14/11	22/5
		ASYG272	-/-	-/-	-/-	-/-	-/-	-/-	-/-	-/-	18/7	28/10	18/9	4/0	9/11	15/9
		ASYG273	-/-	-/-	-/-	-/-	-/-	-/-	-/-	-/-	21/11	11/6	10/3	2/0	17/14	11/8
		ASYG274	-/-	-/-	-/-	-/-	-/-	-/-	-/-	-/-	13/2	21/2	17/8	6/11	14/17	10/19
		ASYG275	-/-	-/-	-/-	-/-	-/-	-/-	-/-	-/-	20/3	14/0	17/0	3/0	7/4	10/4
B	*mrc1*	ASY558	137/136	176/144	230/130	61/148	74/89	17/60	69/57	151/81	191/66	170/58	206/79	122/118	58/356	191/76
		ASYG158	-/-	-/-	-/-	-/-	-/-	-/-	-/-	201/70	218/88	160/62	302/90	144/114	66/244	200/114
		ASYG159	-/-	-/-	-/-	-/-	-/-	-/-	-/-	-/-	141/68	100/41	187/57	112/74	35/184	149/75
		ASYG160	-/-	-/-	-/-	-/-	-/-	-/-	-/-	94/40	119/47	59/25	157/61	78/61	36/136	128/66
		ASYG161	-/-	-/-	-/-	-/-	-/-	-/-	-/-	167/52	179/74	151/57	149/100	134/91	34/200	170/93
		ASYG162	-/-	-/-	-/-	-/-	-/-	-/-	-/-	103/44	177/57	148/44	215/82	147/99	41/198	135/81
		ASYG163	-/-	-/-	-/-	-/-	-/-	-/-	-/-	143/53	171/77	158/49	206/131	171/151	56/246	158/43
		ASYG164	-/-	-/-	-/-	-/-	-/-	-/-	-/-	194/56	206/123	229/52	306/89	182/131	71/260	227/68
		ASYG266	-/-	-/-	-/-	-/-	-/-	-/-	-/-	172/108	198/66	208/60	220/72	178/121	42/211	165/57
		ASYG267	-/-	-/-	-/-	43/31	4/0	20/0	112/39	153/53	198/66	196/52	230/67	159/127	41/218	186/56
		ASYG268	-/-	-/-	194/95	93/95	65/65	14/0	91/39	142/65	269/76	239/50	283/92	164/100	58/235	197/53
		ASYG269	-/-	-/-	-/-	-/-	-/-	-/-	-/-	48/42	220/65	175/51	264/120	151/105	47/228	169/54
C	*mrc1-C14*	ASY639	204/183	269/269	373/204	126/251	61/126	33/129	186/165	163/188	225/132	182/53	246/78	147/140	82/249	235/128
		ASYG187	-/-	-/-	-/-	-/-	-/-	-/-	-/-	203/113	205/102	211/71	242/92	169/136	89/256	244/111
		ASYG188	-/-	-/-	-/-	-/-	-/-	-/-	-/-	-/-	211/118	201/89	211/96	148/138	37/386	170/114
		ASYG189	-/-	-/-	-/-	-/-	-/-	-/-	-/-	60/38	320/127	201/89	387/93	237/131	136/272	321/145
		ASYG190	-/-	-/-	-/-	-/-	-/-	-/-	-/-	-/-	170/66	170/70	184/89	137/131	44/349	146/92
		ASYG191	-/-	-/-	-/-	-/-	-/-	-/-	-/-	54/44	179/69	174/54	240/82	165/114	49/335	181/86
		ASYG263	-/-	-/-	-/-	-/-	-/-	-/-	-/-	79/51	113/83	119/84	126/90	97/112	17/0	117/91
		ASYG264	-/-	-/-	-/-	-/-	-/-	-/-	-/-	-/-	100/101	106/100	132/106	70/117	21/226	130/88
		ASYG276	-/-	-/-	-/-	-/-	-/-	-/-	-/-	134/59	121/102	135/59	107/92	103/109	29/278	142/112
		ASYG277	-/-	-/-	-/-	-/-	-/-	-/-	-/-	-/-	108/88	120/73	117/74	97/95	22/231	110/86
		ASYG278	-/-	-/-	-/-	-/-	-/-	-/-	-/-	-/-	163/84	154/69	189/90	137/130	57/321	167/103
		ASYG279	-/-	-/-	-/-	-/-	-/-	-/-	-/-	90/39	115/66	118/53	133/60	102/117	34/315	128/80
D	*mrc1 swr1*	ASY749	136/89	118/81	197/73	110/89	67/57	31/0	102/41	126/37	129/48	160/24	205/75	141/88	67/203	148/66
		ASYG230	-/-	-/-	-/-	274/184	150/111	70/55	252/78	252/106	143/61	203/52	250/82	189/116	106/259	212/100
		ASYG231	-/-	-/-	233/84	96/111	24/36	59/136	62/108	69/32	51/27	69/33	86/23	18/36	18/169	76/28
		ASYG232	-/-	-/-	287/128	137/142	58/67	51/62	139/62	136/51	147/67	125/47	194/65	142/127	56/233	170/95
		ASYG233	-/-	-/-	-/-	-/-	-/-	-/-	-/-	101/71	201/77	210/74	262/129	120/117	46/247	215/60
		ASYG234	-/-	-/-	359/166	223/180	131/99	58/0	206/103	273/86	291/120	352/103	388/125	288/161	89/337	262/66
		ASYG235	-/-	-/-	-/-	-/-	-/-	-/-	-/-	216/85	211/109	268/49	341/90	217/128	72/290	223/64
		ASYG236	-/-	-/-	-/-	-/-	-/-	-/-	-/-	127/58	139/50	149/38	223/100	139/106	43/210	188/88
		ASYG237	-/-	-/-	-/-	233/164	113/79	54/94	211/88	189/85	207/97	287/51	326/108	239/146	102/290	259/66
		ASYG238	-/-	-/-	-/-	-/-	-/-	-/-	-/-	99/53	210/60	314/75	310/87	206/144	81/280	207/99
		ASYG239	-/-	-/-	-/-	-/-	-/-	-/-	-/-	181/90	175/71	289/63	272/77	172/137	41/0	192/60
E	*mrc1-C14 swr1*	ASY746	100/69	122/93	205/91	62/90	69/54	21/0	64/40	94/41	228/71	206/44	248/68	130/106	45/247	177/61
		ASYG200	-/-	-/-	-/-	-/-	-/-	99/94	249/101	541/137	497/161	268/62	544/340	180/122	185/340	468/100
		ASYG201	-/-	-/-	-/-	-/-	-/-	82/176	333/63	276/108	354/109	107/21	389/87	182/132	161/300	344/67
		ASYG202	-/-	-/-	-/-	192/182	104/91	65/99	202/95	246/98	299/84	317/66	424/103	210/153	93/312	391/93
		ASYG203	-/-	-/-	-/-	-/-	-/-	-/-	-/-	-/-	444/113	260/54	332/106	175/113	109/275	296/60
		ASYG204	-/-	-/-	-/-	-/-	-/-	-/-	393/74	454/134	633/188	357/91	683/142	413/148	152/390	503/119
		ASYG205	433/220	-/-	-/-	322/251	174/107	118/108	293/119	310/87	327/84	346/74	502/112	258/182	140/328	400/79
		ASYG206	-/-	-/-	-/-	-/-	-/-	-/-	-/-	194/59	241/75	220/44	303/81	202/132	69/277	109/49
		ASYG208	-/-	-/-	-/-	-/-	-/-	-/-	-/-	194/51	215/57	235/63	325/123	183/156	68/267	238/82
		ASYG209	-/-	-/-	-/-	417/355	290/130	127/166	380/199	566/171	586/234	191/65	290/88	132/146	32/0	177/63
		ASYG210	-/-	-/-	-/-	-/-	-/-	-/-	-/-	-/-	503/118	321/85	352/128	210/164	80/294	289/82

Supplementary Figure 2

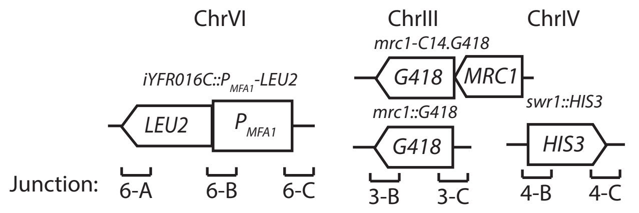

Panel	Genotype	Strain	6-A	6-B	6-C	3-B	3-C	4-B	4-C
F	*swr1*	ASY566	19/8	26/10	13/15	n.a.	n.a.	9/9	14/4
		ASYG176	22/6	24/6	23/6	n.a.	n.a.	12/8	11/1
		ASYG178	24/14	23/11	13/20	n.a.	n.a.	16/12	10/1
		ASYG179	24/5	16/9	14/10	n.a.	n.a.	19/12	13/18
		ASYG180	18/12	25/5	18/11	n.a.	n.a.	21/8	11/9
		ASYG261	22/10	16/5	13/16	n.a.	n.a.	6/6	12/6
		ASYG262	23/19	22/16	22/20	n.a.	n.a.	13/8	12/17
		ASYG271	22/12	35/10	16/17	n.a.	n.a.	10/9	14/8
		ASYG272	29/15	22/6	29/18	n.a.	n.a.	19/10	17/15
		ASYG273	11/11	20/9	17/20	n.a.	n.a.	10/8	13/13
		ASYG274	15/9	35/6	20/18	n.a.	n.a.	8/7	10/7
		ASYG275	7/5	18/9	12/18	n.a.	n.a.	8/10	9/8
G	*mrc1*	ASY558	176/98	253/78	137/185	189/70	238/62	n.a.	n.a.
		ASYG158	229/134	310/106	193/186	236/124	311/105	n.a.	n.a.
		ASYG159	169/80	212/63	163/176	130/64	200/70	n.a.	n.a.
		ASYG160	182/76	206/72	183/98	135/59	216/64	n.a.	n.a.
		ASYG161	177/111	248/85	218/132	184/87	217/92	n.a.	n.a.
		ASYG162	195/128	312/103	208/134	200/99	208/83	n.a.	n.a.
		ASYG163	253/162	284/98	253/173	192/91	318/104	n.a.	n.a.
		ASYG164	316/129	322/138	270/165	257/73	289/86	n.a.	n.a.
		ASYG266	233/118	297/85	202/187	208/63	226/59	n.a.	n.a.
		ASYG267	247/117	296/66	214/155	196/57	239/77	n.a.	n.a.
		ASYG268	241/122	297/99	232/152	244/80	208/68	n.a.	n.a.
		ASYG269	190/112	266/91	193/128	297/65	201/63	n.a.	n.a.
H	*mrc1-C14*	ASY639	294/173	310/125	321/185	271/102	416/156	n.a.	n.a.
		ASYG187	294/166	332/114	313/169	257/90	341/131	n.a.	n.a.
		ASYG188	216/148	277/98	175/353	206/97	206/102	n.a.	n.a.
		ASYG189	216/148	374/131	318/155	464/87	387/124	n.a.	n.a.
		ASYG190	186/131	210/82	165/282	181/90	212/82	n.a.	n.a.
		ASYG191	243/150	288/122	176/303	206/83	232/92	n.a.	n.a.
		ASYG263	126/148	183/95	91/264	112/92	135/96	n.a.	n.a.
		ASYG264	81/96	142/91	79/189	116/85	106/77	n.a.	n.a.
		ASYG276	154/140	176/68	128/252	142/84	163/64	n.a.	n.a.
		ASYG277	119/132	175/81	84/259	92/65	115/112	n.a.	n.a.
		ASYG278	221/163	271/106	195/323	221/110	278/118	n.a.	n.a.
		ASYG279	164/121	202/105	157/294	201/87	178/98	n.a.	n.a.
I	*mrc1 swr1*	ASY749	198/96	238/66	193/160	166/51	231/65	143/66	99/68
		ASYG230	297/167	335/109	307/219	314/69	387/103	176/86	141/119
		ASYG231	179/107	193/63	305/160	104/21	443/103	120/42	62/105
		ASYG232	238/145	260/75	268/163	194/61	349/85	175/52	96/104
		ASYG233	234/188	303/92	258/206	224/70	279/92	204/100	108/132
		ASYG234	366/192	455/140	410/225	408/104	452/106	301/103	174/126
		ASYG235	323/156	426/142	409/247	259/85	389/100	257/90	162/123
		ASYG236	171/125	266/74	258/150	162/47	310/89	178/77	84/90
		ASYG237	617/241	794/236	678/548	321/83	431/97	224/83	151/128
		ASYG238	270/127	370/135	310/155	287/58	383/92	465/126	286/158
		ASYG239	302/177	437/94	363/236	245/63	401/122	189/86	108/169
J	*mrc1-C14 swr1*	ASY746	221/126	252/85	160/177	206/56	203/72	199/93	168/122
		ASYG200	571/183	676/179	643/222	356/77	647/184	5653/155	572/222
		ASYG201	372/166	423/119	383/187	310/55	592/110	520/106	384/155
		ASYG202	426/191	562/119	530/259	402/94	541/136	321/115	229/149
		ASYG203	303/112	307/80	300/167	314/59	319/68	272/84	201/106
		ASYG204	601/246	588/164	602/271	606/111	531/144	595/188	428/211
		ASYG205	513/213	569/141	511/291	456/100	540/161	354/120	276/159
		ASYG206	274/156	335/86	307/202	258/67	322/124	172/91	179/133
		ASYG208	297/161	392/104	371/238	273/68	324/88	205/85	166/115
		ASYG209	209/128	294/84	237/174	193/76	210/93	143/94	122/111
		ASYG210	308/153	374/94	362/195	342/94	307/110	362/178	268/188

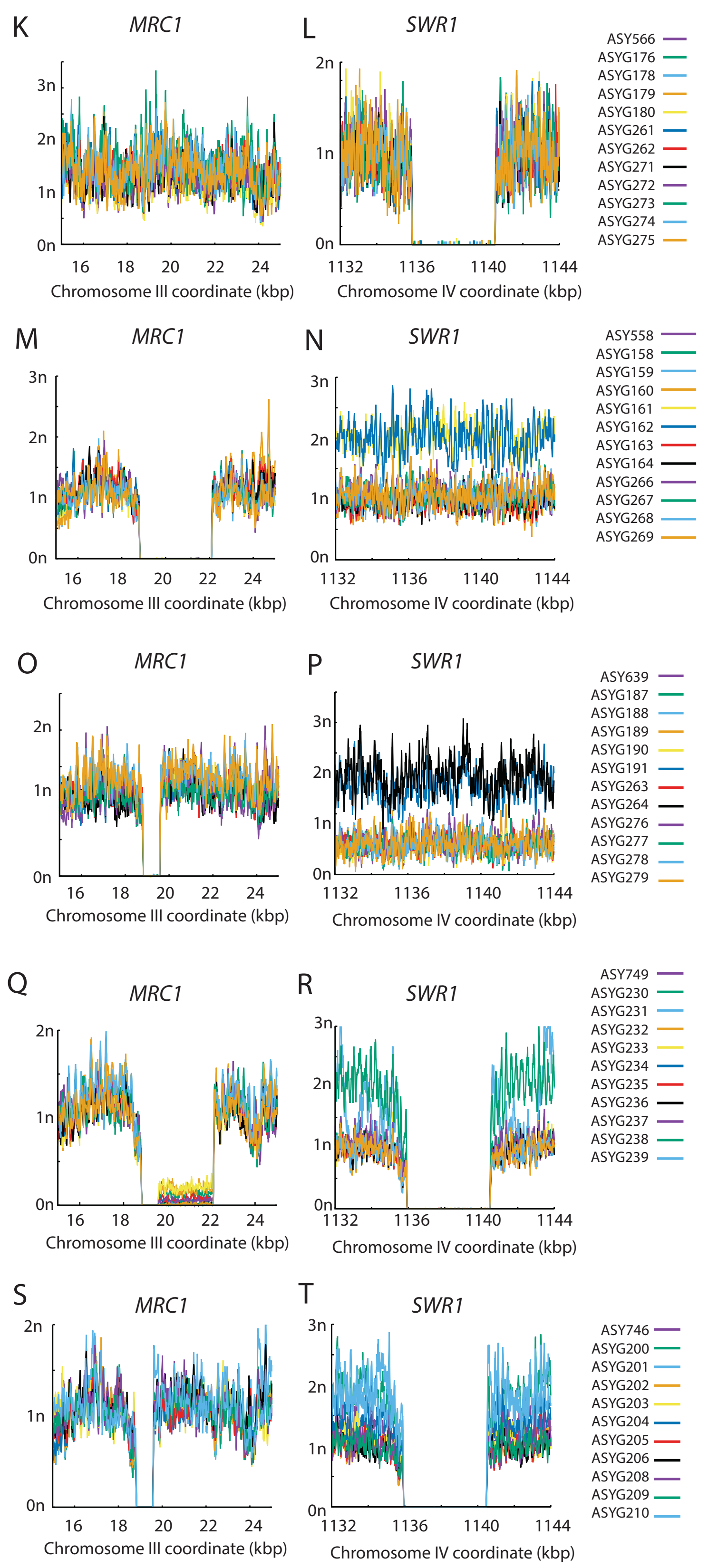

Supplementary Figure 2. Identification of the engineered chromosomal features in sGCR assay strains by WGS. A-J. Evidence for each engineered junction displayed as in Supplementary Fig. 1. **K-T.** Read-depth analysis of the *MRC1* and *SWR1*-containing chromosomal regions displayed as in Supplementary Fig. 1.

Supplementary Figure 3.

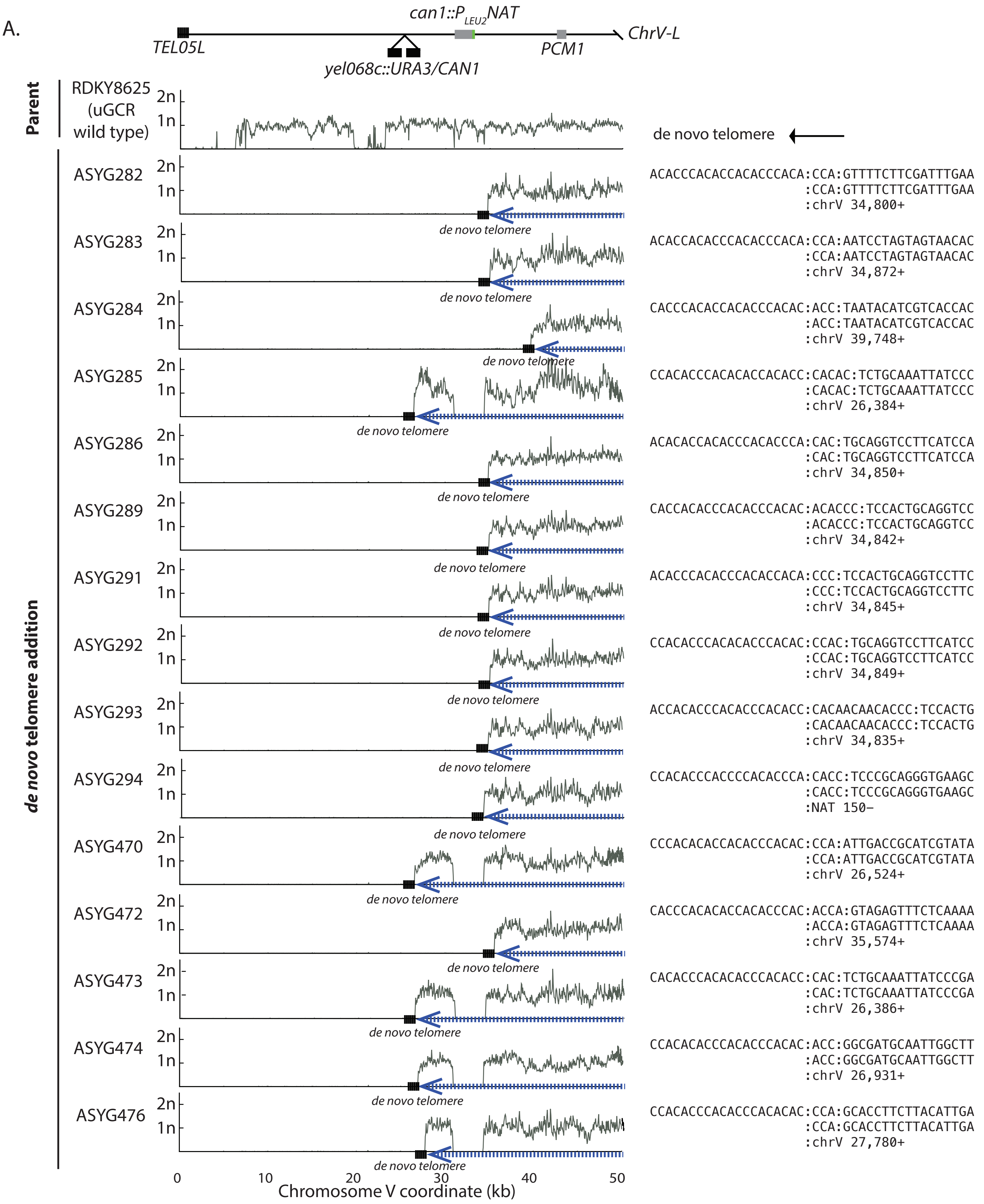

Supplementary Figure 3.

B.

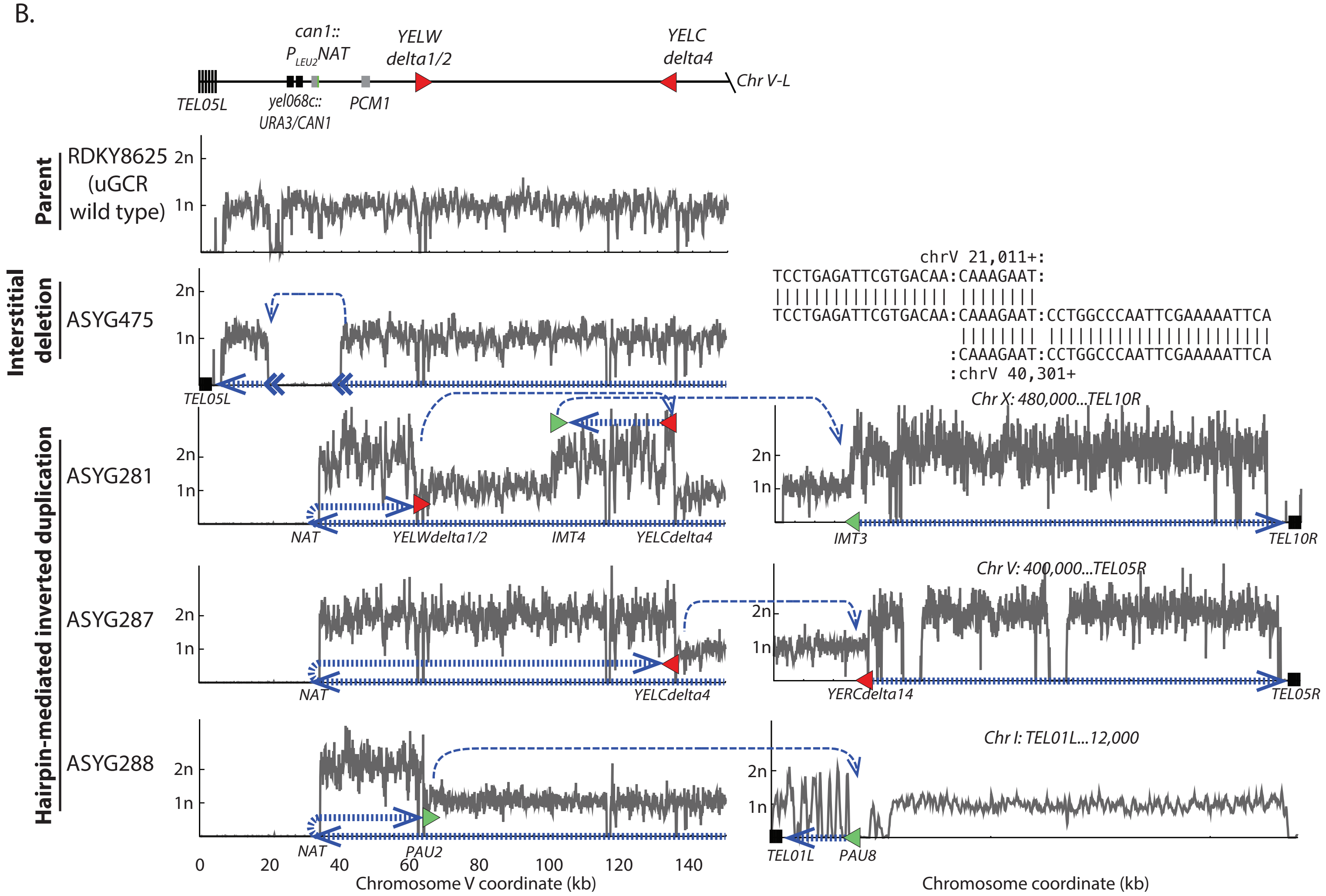

Supplementary Figure 3. Analysis of wild-type GCRs isolated in the uGCR assay. Analysis of *de novo* telomere additions (**A**), interstitial deletions and hairpin-mediated inverted duplications (**B**) isolated from a wild-type uGCR parental strain (RDKY8625). Copy-number analysis of the sequenced parental strain and GCR-containing strains shows that all GCRs include deletion of the *CAN1/URA3*-containing portion of chromosome V L (left) and either duplication of a terminal region of a target chromosome or addition of a *de novo* telomere or an interstitial deletion whose junction sequences can be identified (right). The thick-hashed blue arrow indicates sequences within the GCR; the thin dashed blue arrow indicates connectivity between portions of the GCR that map to different regions of the reference chromosome(s). Multi-copy sequences involved in GCR-related HR events are shown as triangles; red triangles are Ty-related homologies and green triangles are other homologies.

Supplementary Figure 4.

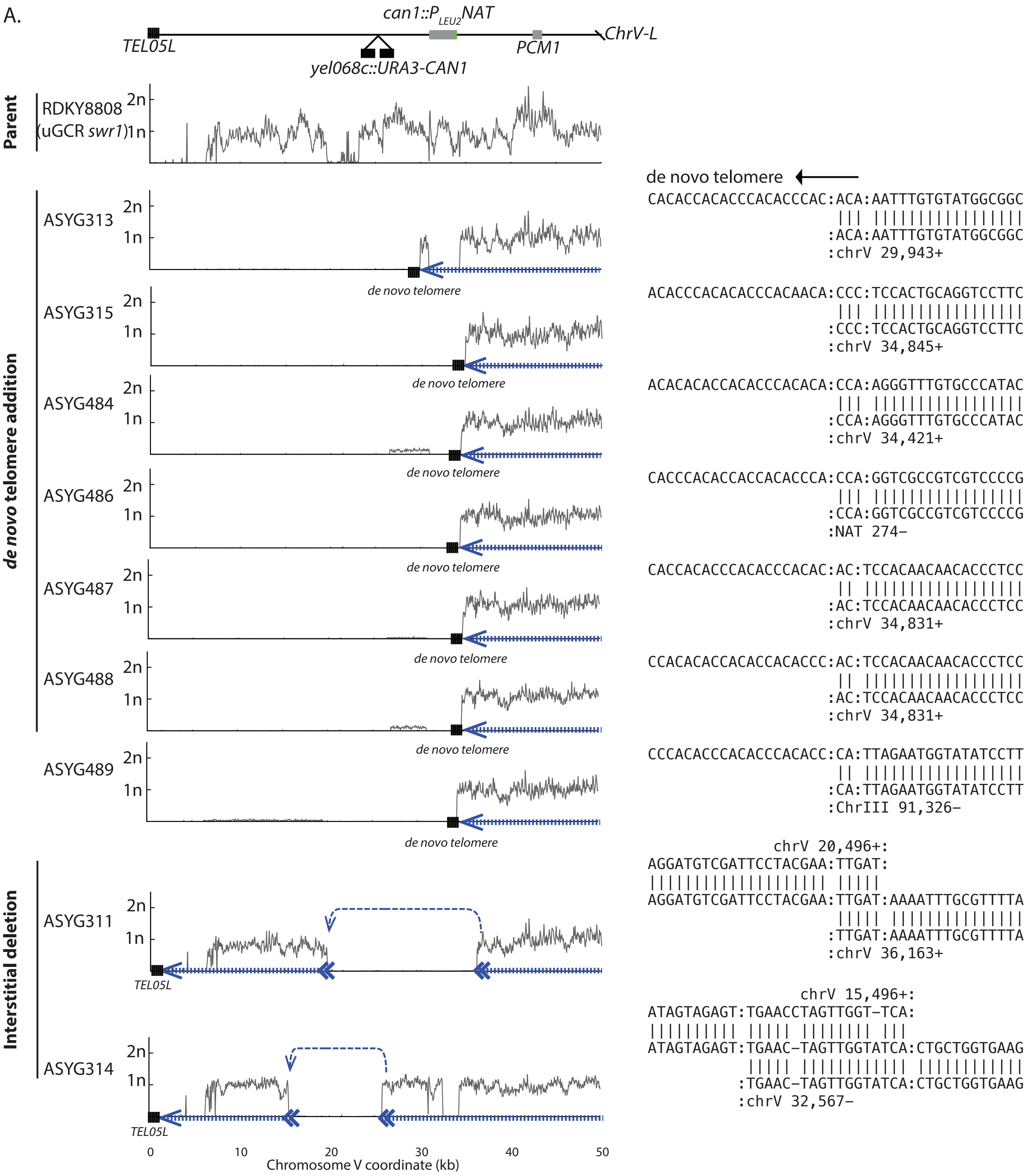

Supplementary Figure 4.

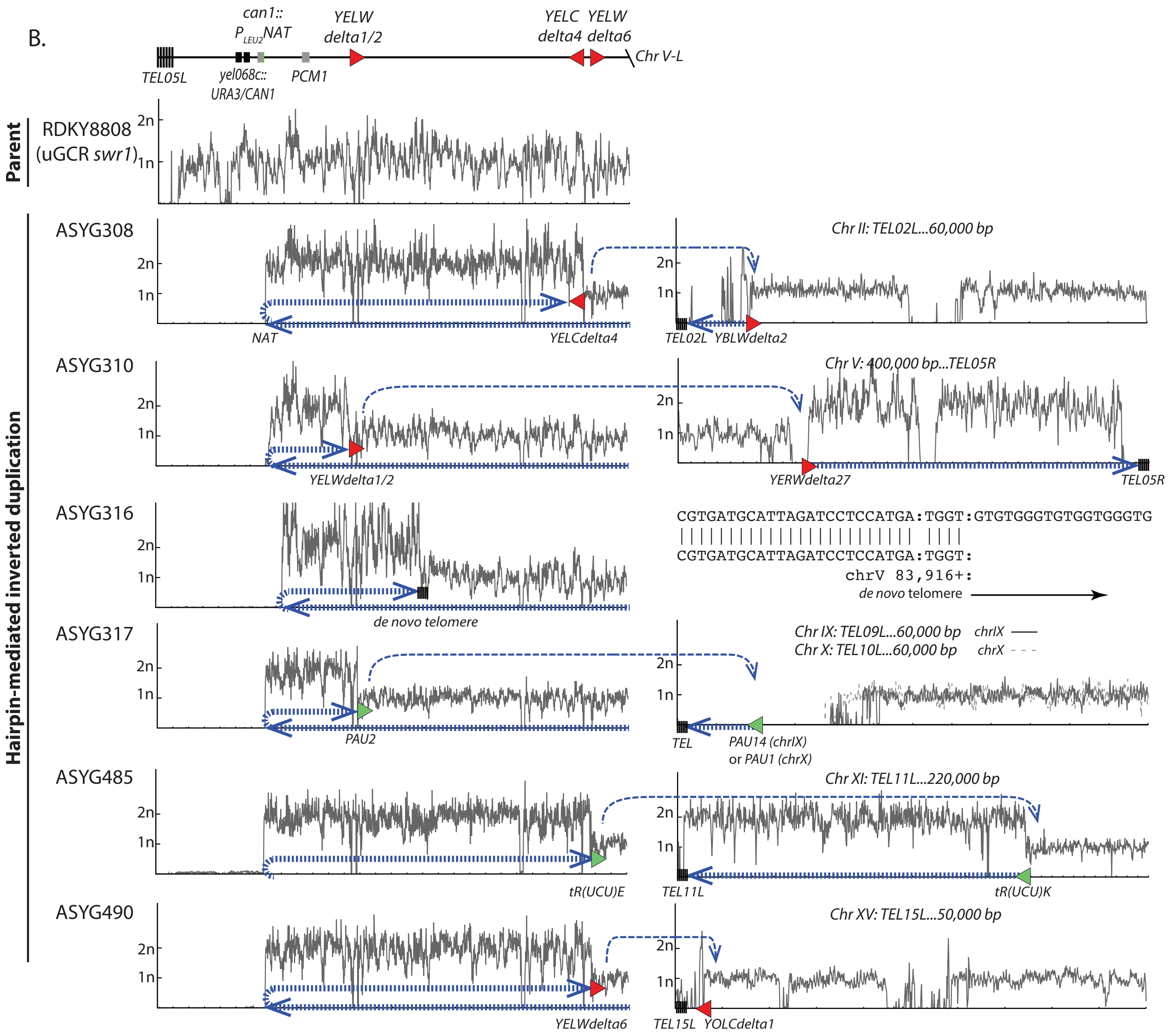

Supplementary Figure 4.

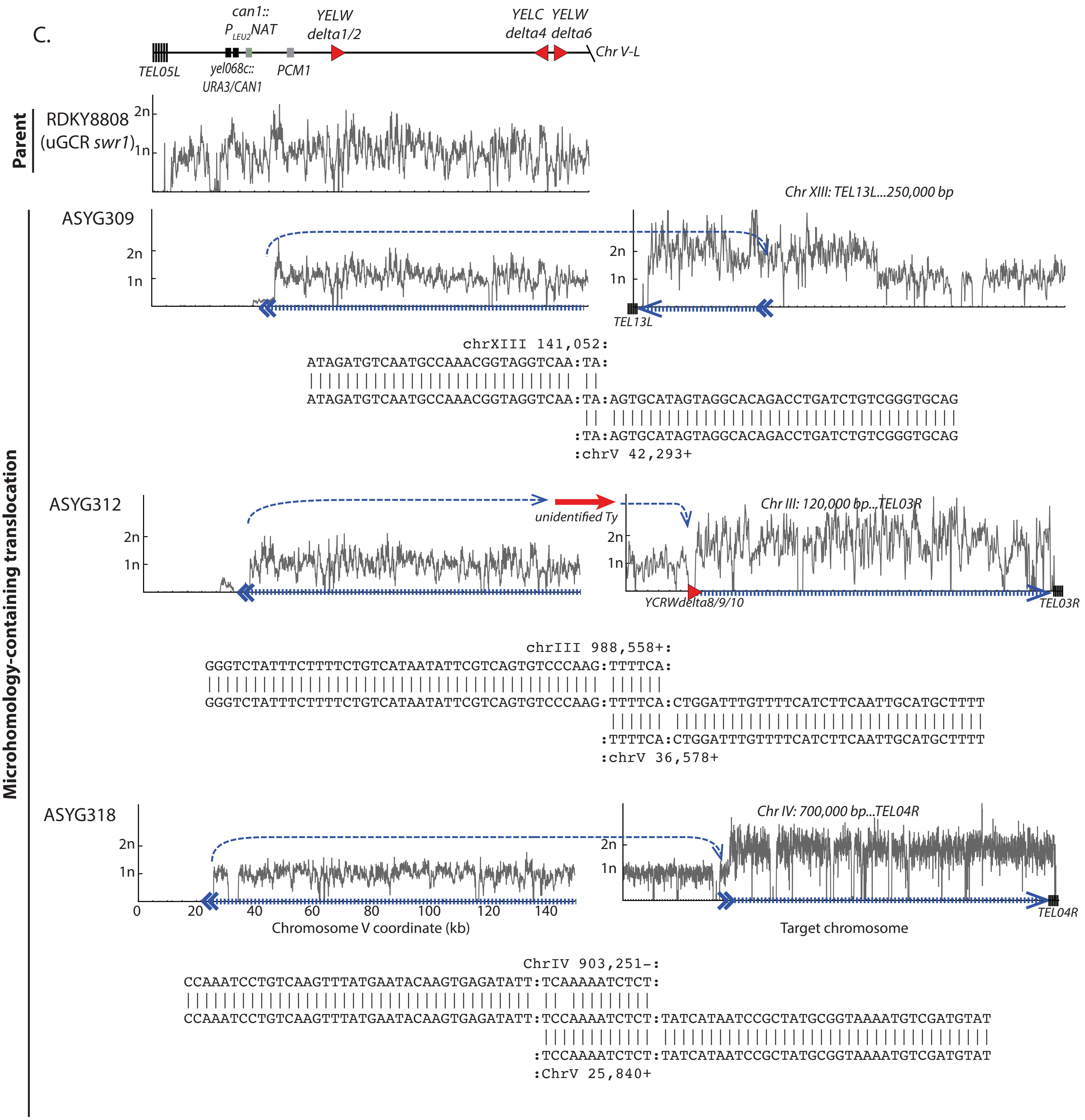

Supplementary Figure 4. Analysis of *swr1Δ* GCRs isolated in the uGCR assay. GCRs derived from a *swr1Δ* uGCR parent strain (RDKY8808) are depicted as described in Supplementary Fig. 3. Observed rearrangements include *de novo* telomere addition-mediated GCRs and interstitial deletions (**A**); hairpin-mediated inverted duplications (**B**); and microhomology-containing translocations (**C**). When more than one GCR is present, the second rearrangement is shown with green arrows.

Supplementary Figure 5.

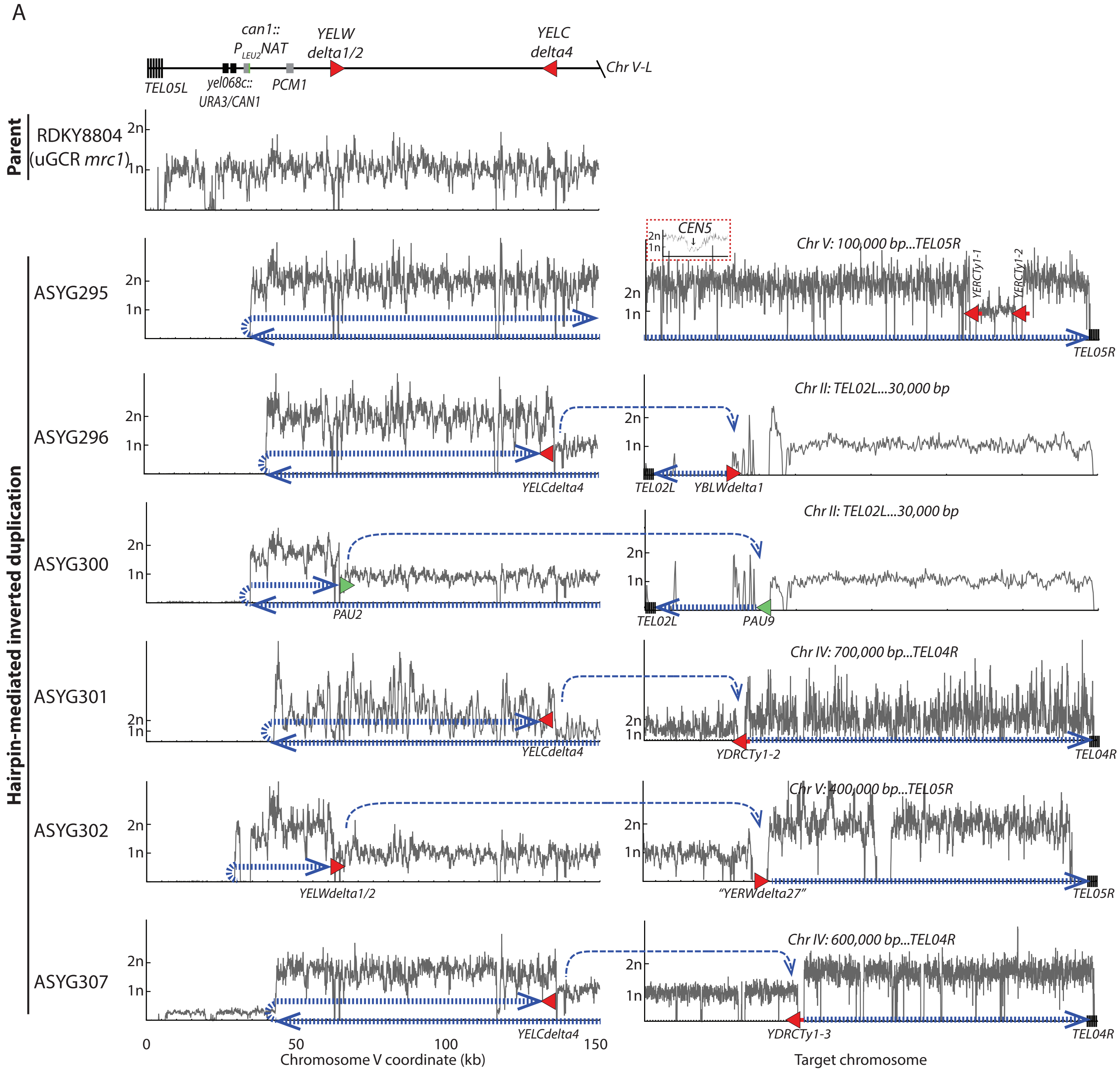

Supplementary Figure 5.

B.

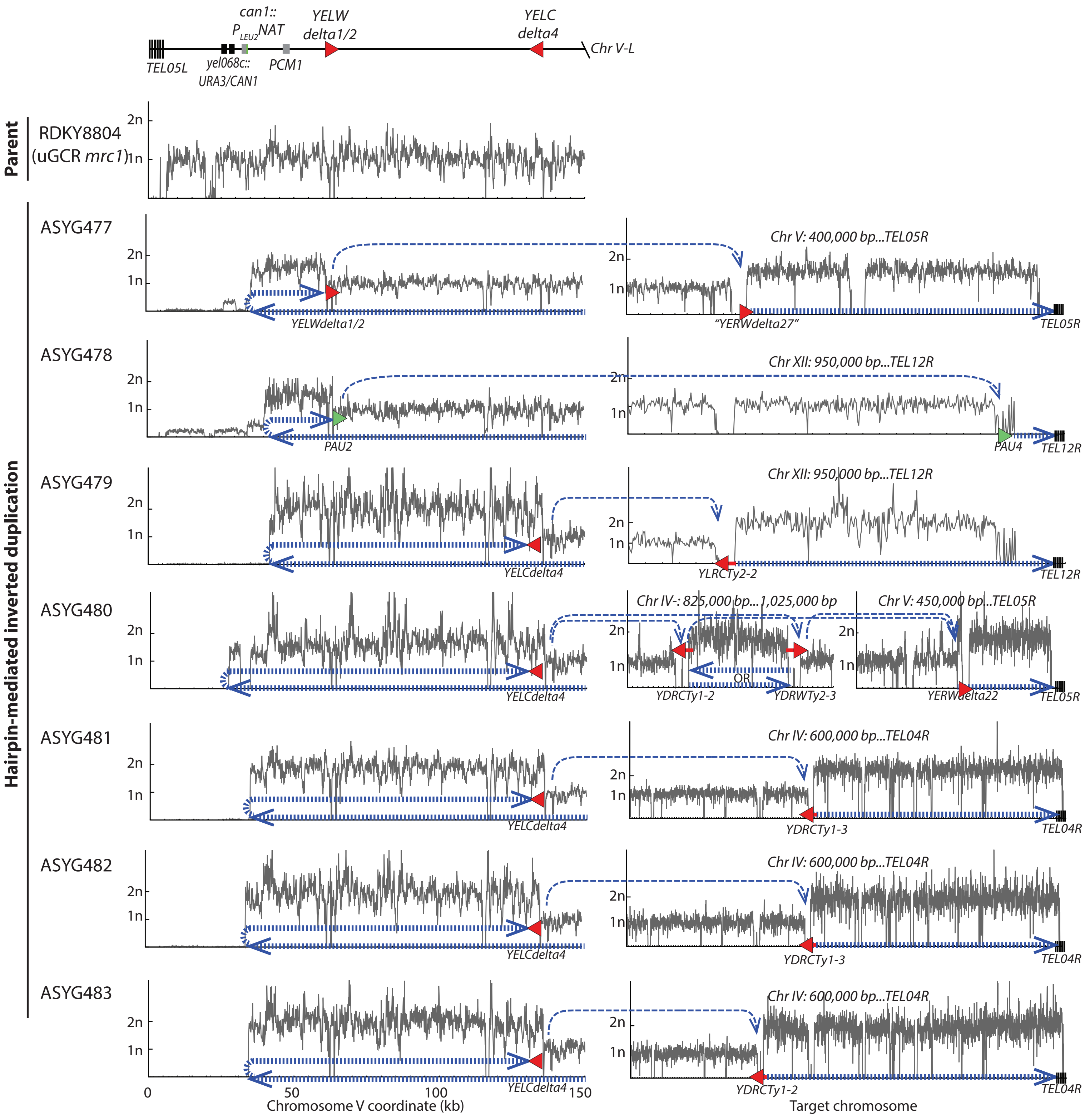

Supplementary Figure 5.

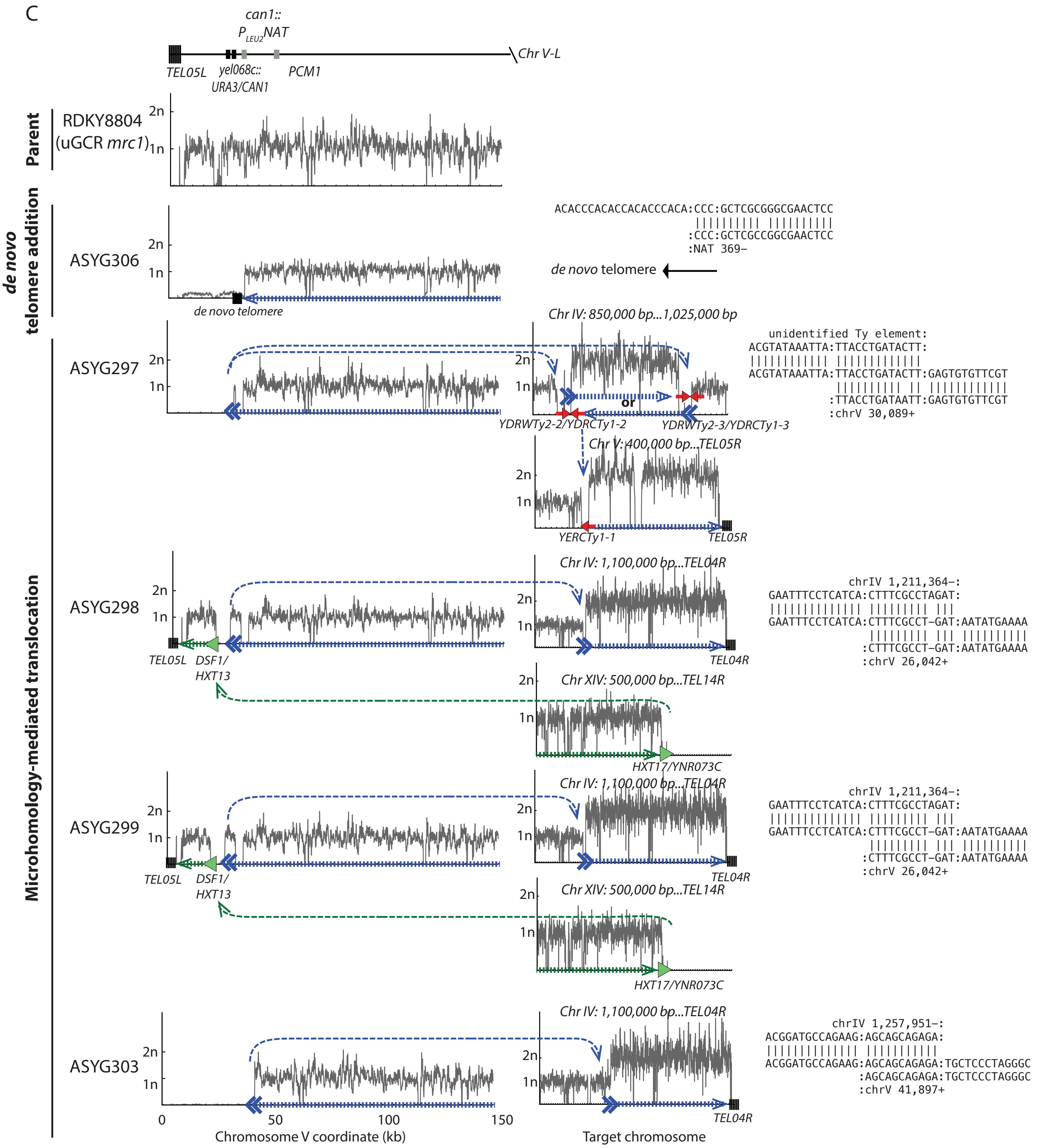

Supplementary Figure 5. Analysis of *mrc1Δ* GCRs isolated in the uGCR assay. GCRs derived from an *mrc1Δ* uGCR parent strain (RDKY8804) are depicted as described in Supplementary Fig. 3. Observed rearrangements include hairpin-mediated inverted duplications (**A and B**); and *de novo* telomere addition-mediated GCRs and microhomology-containing translocations (**C**). When more than one GCR is present, the second rearrangement is shown with green arrows.

Supplementary Figure 6.

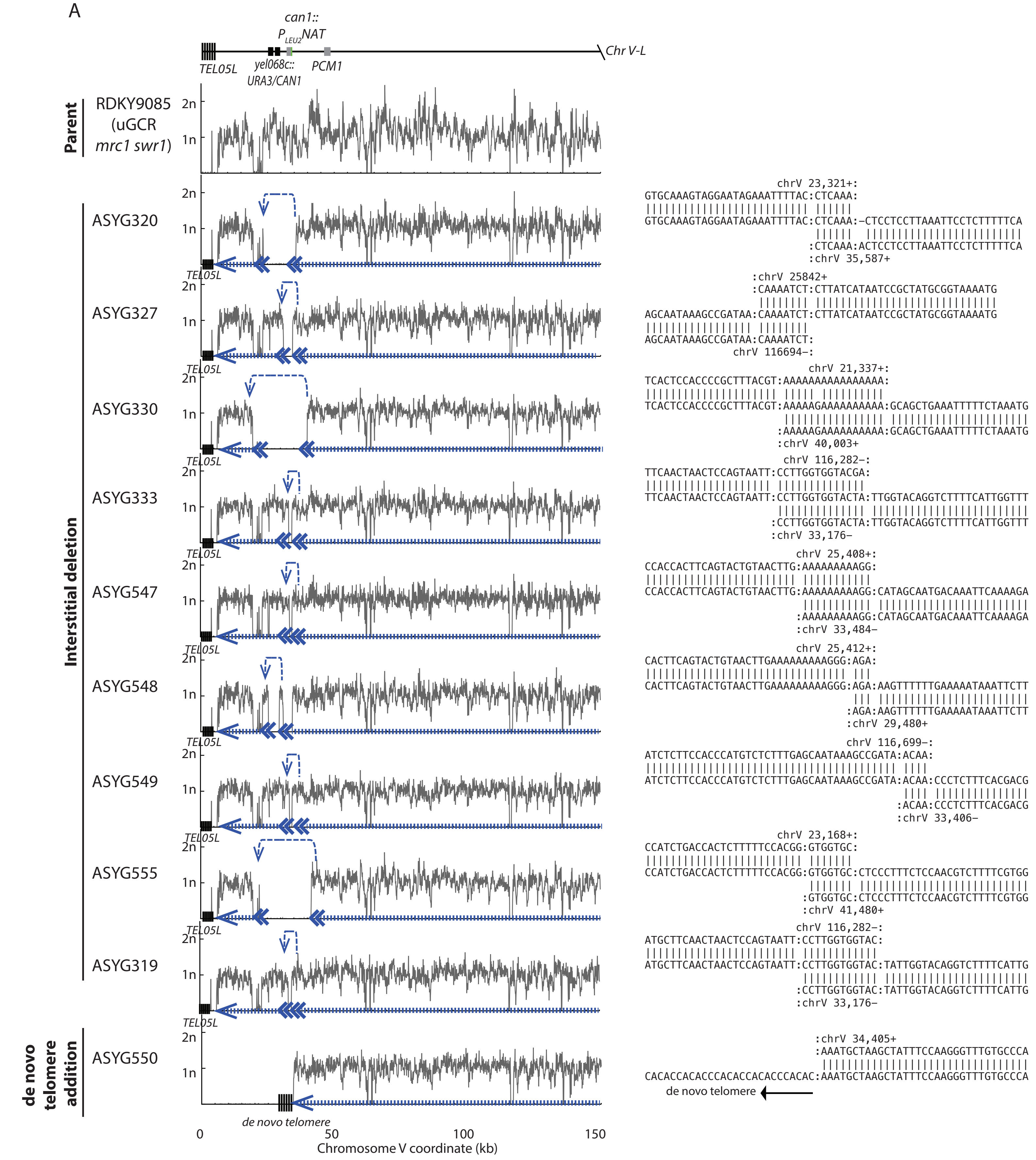

Supplementary Figure 6.

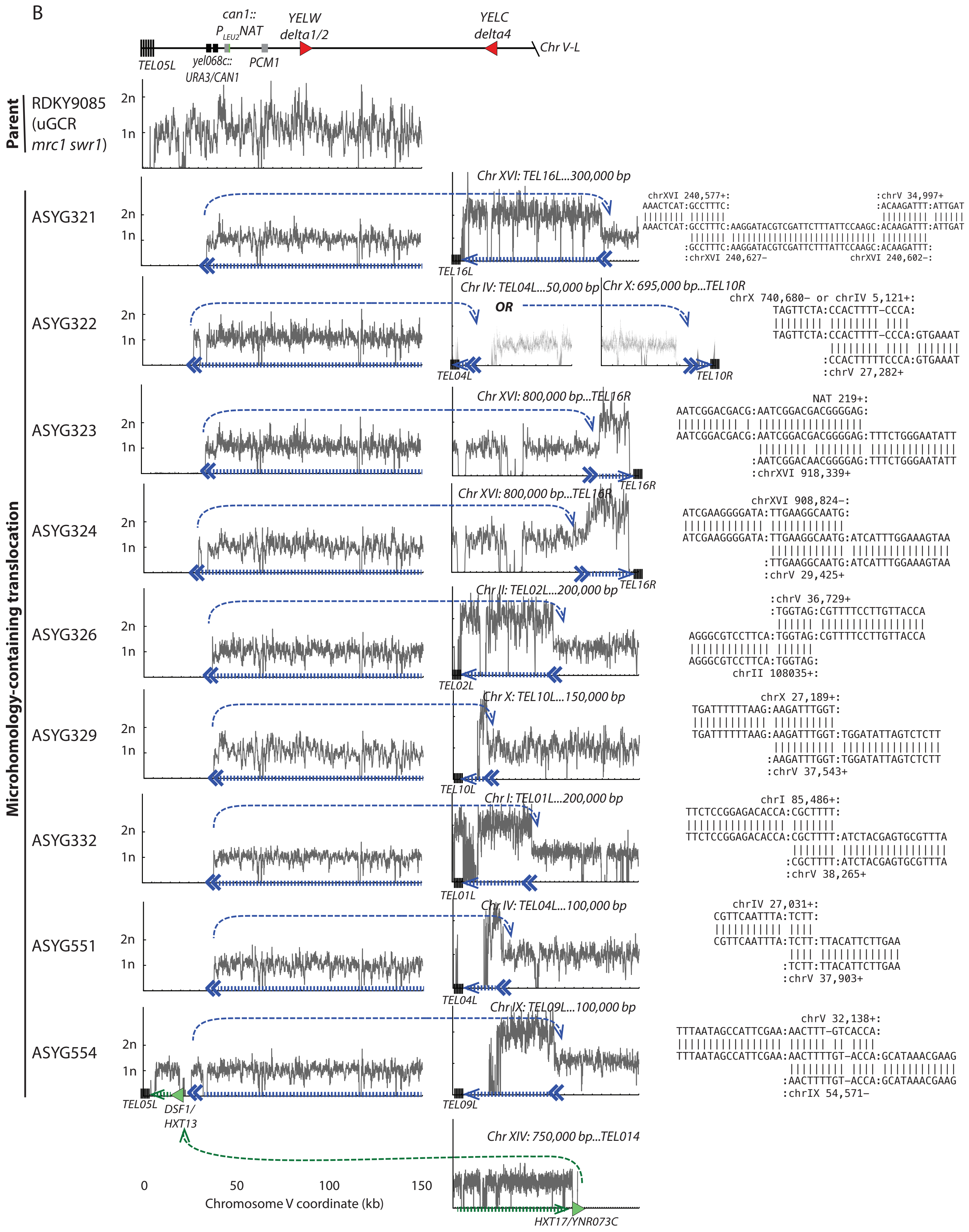

Supplementary Figure 6.

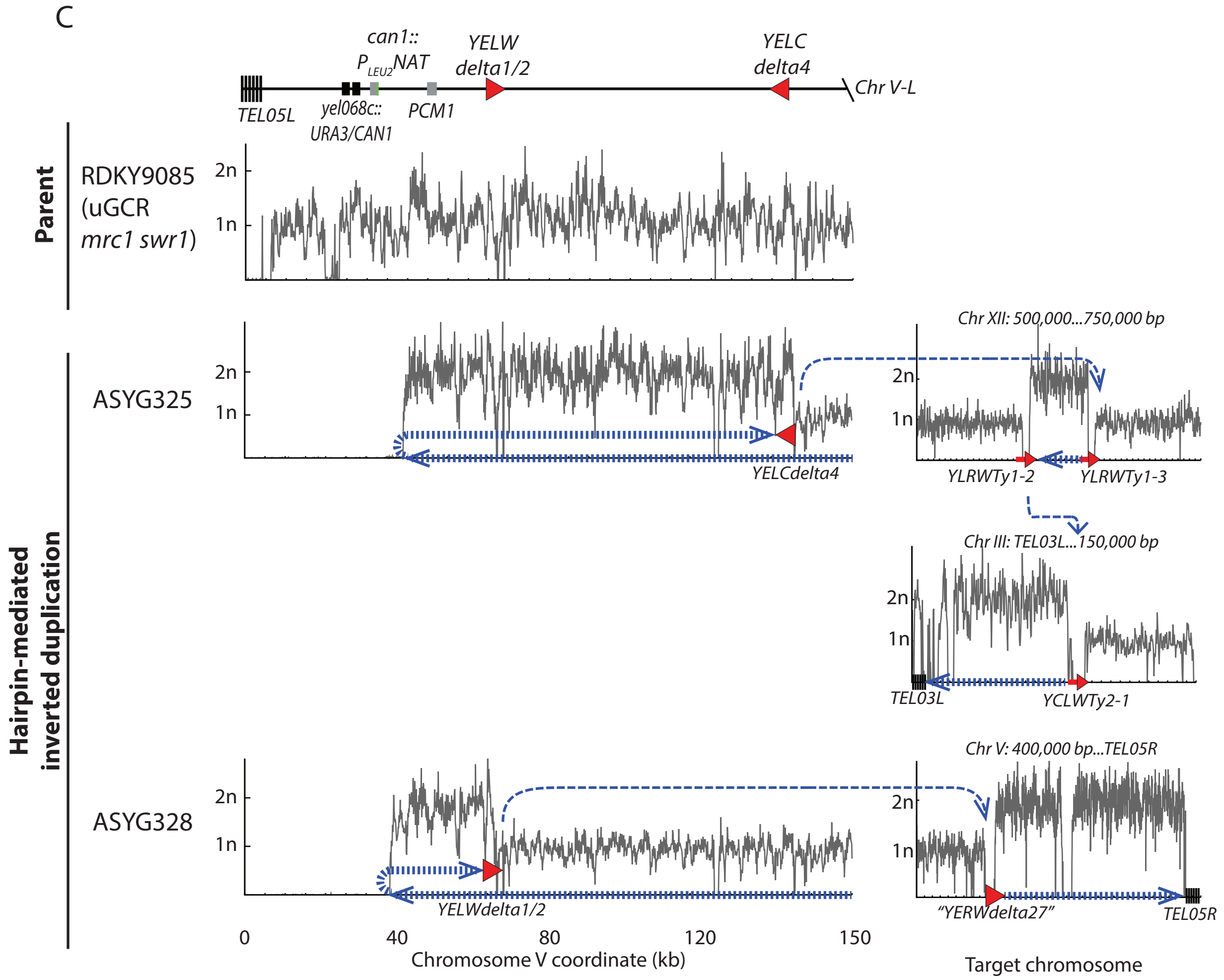

Supplementary Figure 6. Analysis of *mrc1Δ swr1Δ* GCRs isolated in the uGCR assay. GCRs derived from an *mrc1Δ swr1Δ* uGCR parent strain (RDKY9085) are depicted as described in Supplementary Fig. 3. Observed rearrangements include *de novo* telomere addition-mediated GCRs and interstitial deletions (**A**); microhomology-containing translocations (**B**); and hairpin-mediated inverted duplications (**C**). When more than one GCR is present, the second rearrangement is shown with green arrows.

Supplementary Figure 7.

A

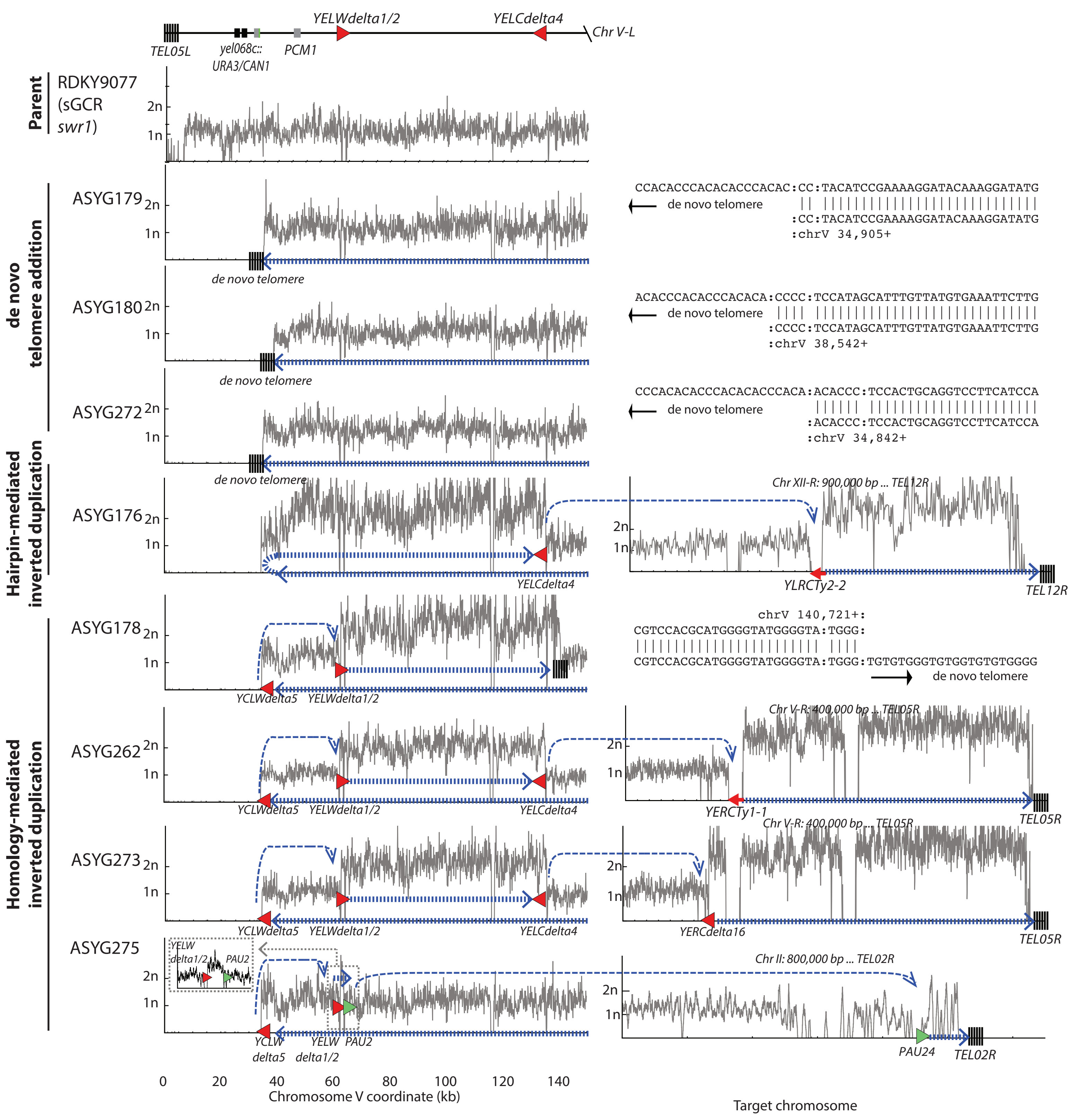

Supplementary Figure 7.

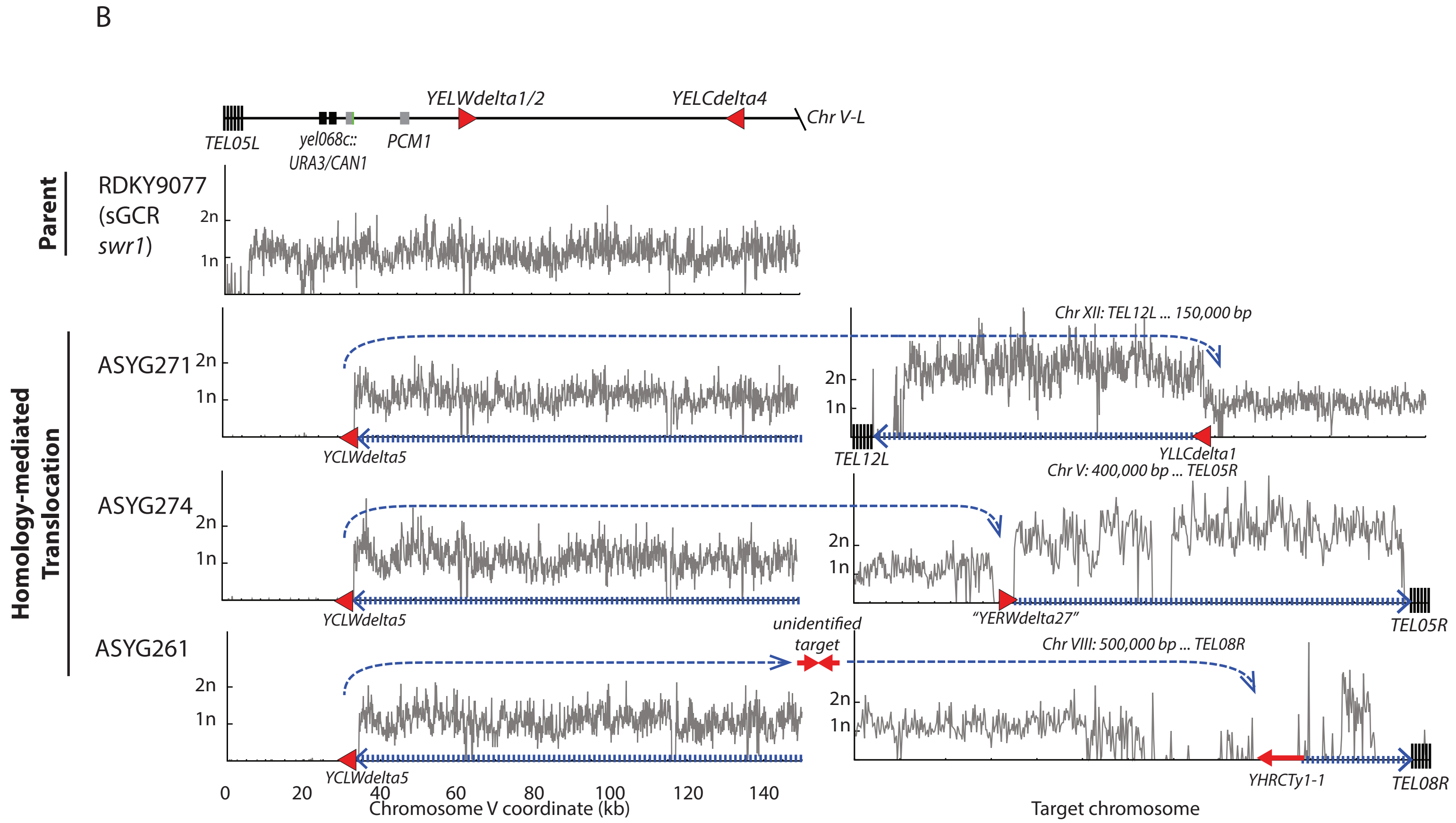

Supplementary Figure 7. Analysis of *swr1Δ* GCRs isolated in the sGCR assay. GCRs derived from a *swr1Δ* sGCR parent strain (RDKY9077) are depicted as described in Supplementary Fig. 3. Observed rearrangements include *de novo* telomere addition-mediated GCRs and interstitial deletions, hairpin-mediated inverted duplications, and homology-mediated inverted duplications (**A**); homology-mediated translocations (**B**).

Supplementary Figure 8.

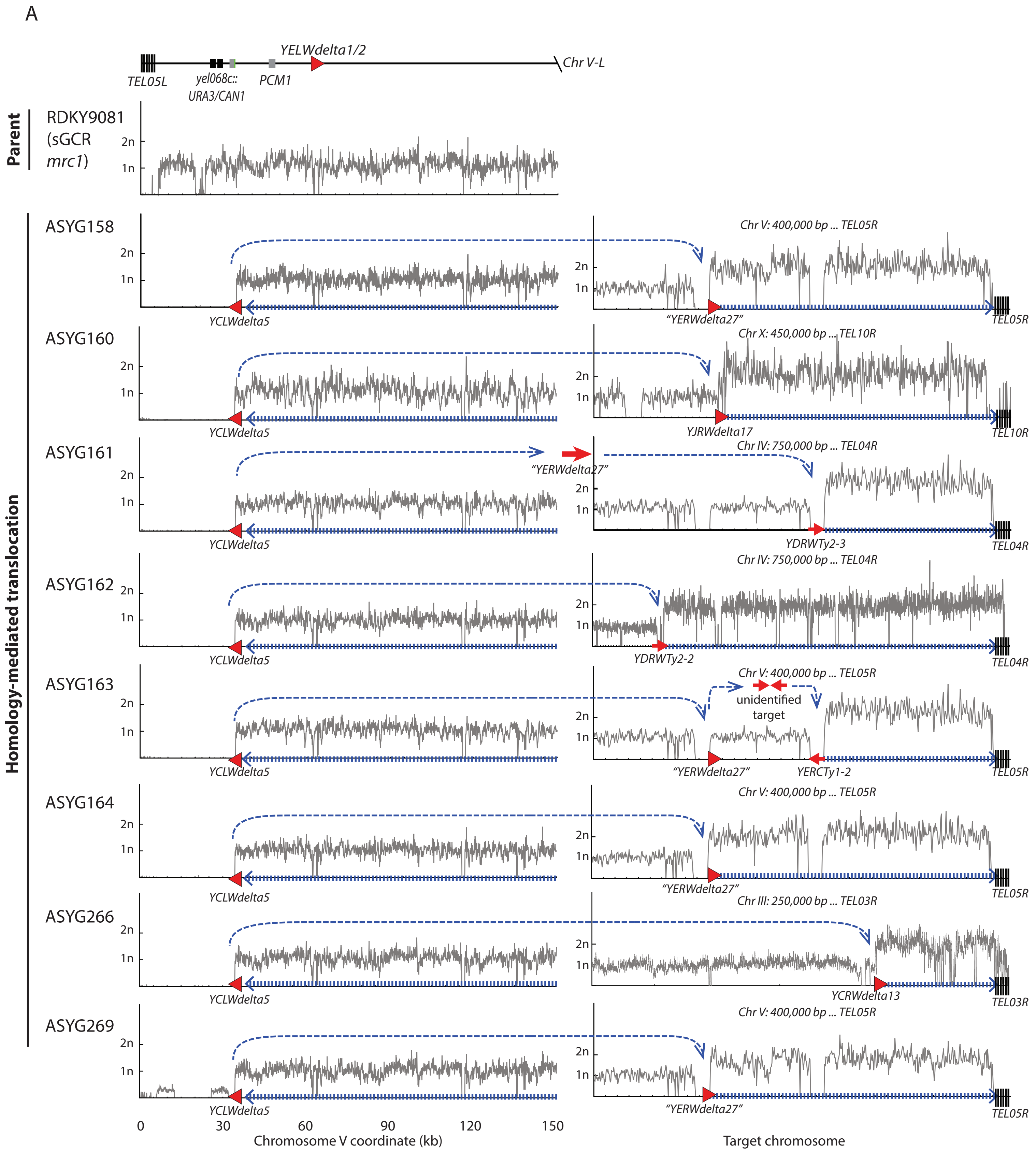

Supplementary Figure 8.

B

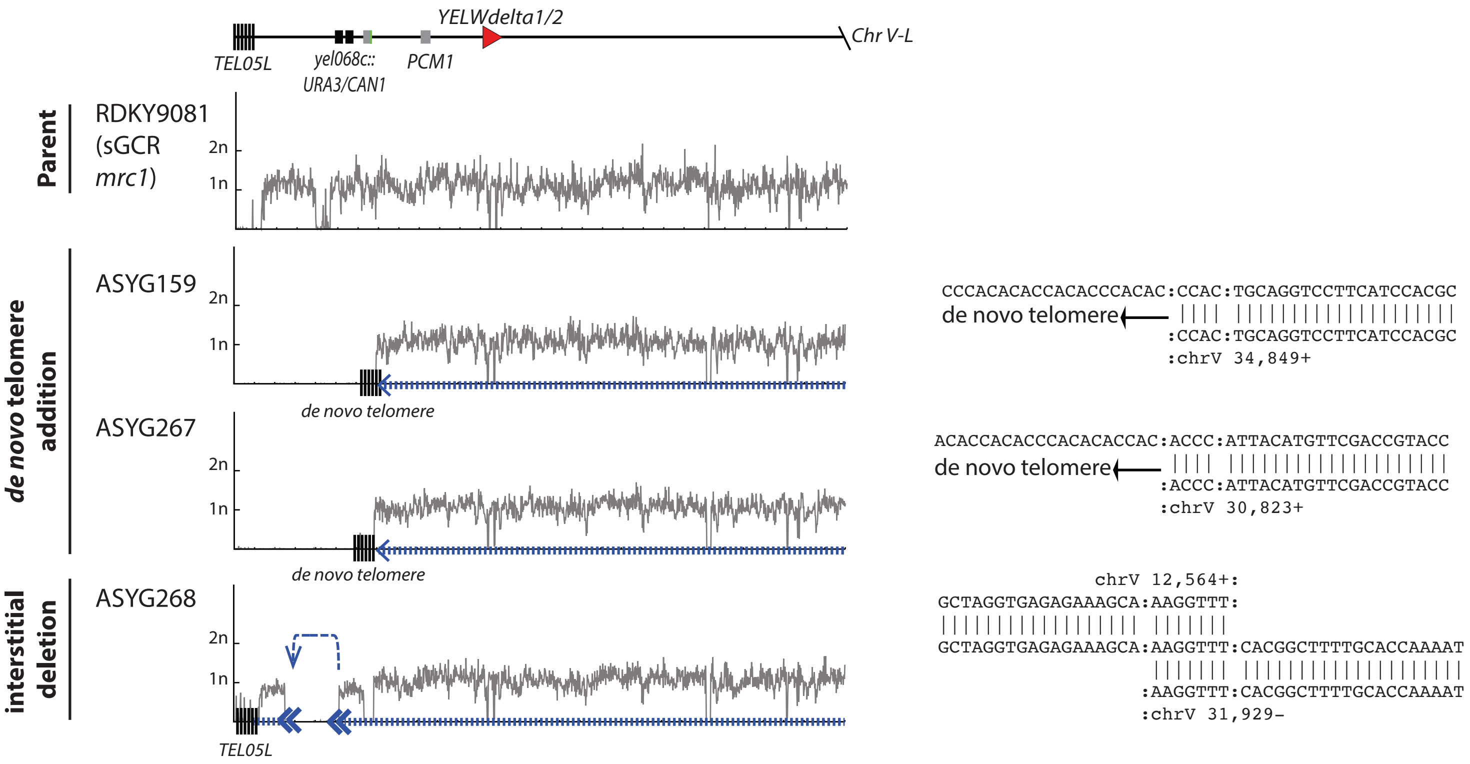

Supplementary Figure 8. Analysis of *mrc1Δ* GCRs isolated in the sGCR assay. GCRs derived from an *mrc1Δ* sGCR parent strain (RDKY9081) are depicted as described in Supplementary Fig. 3. Observed rearrangements include homology-mediated inverted duplications (**A**); and *de novo* telomere addition-mediated GCRs and interstitial deletions (**B**).

Supplementary Figure 9.

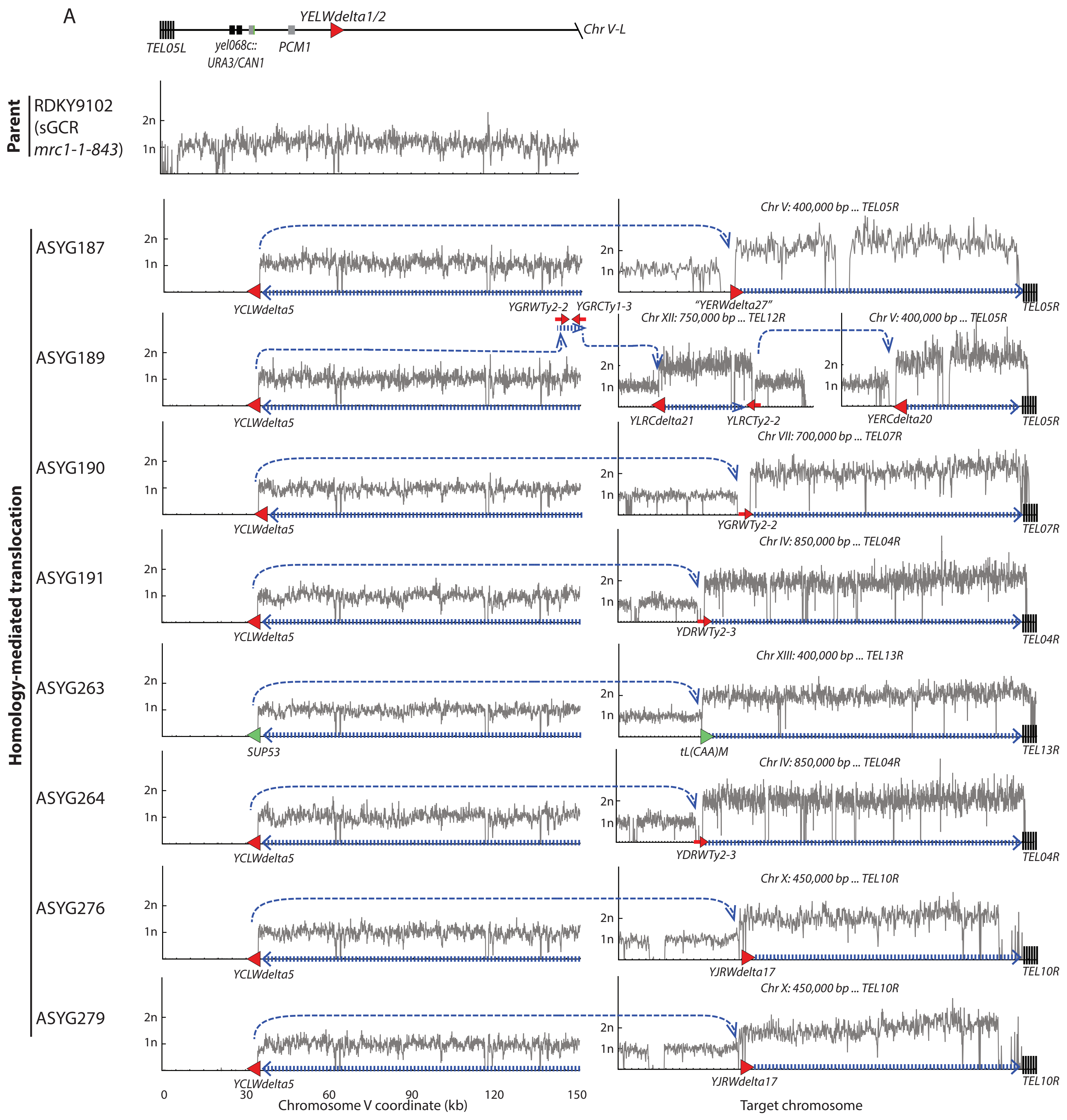

Supplementary Figure 9.

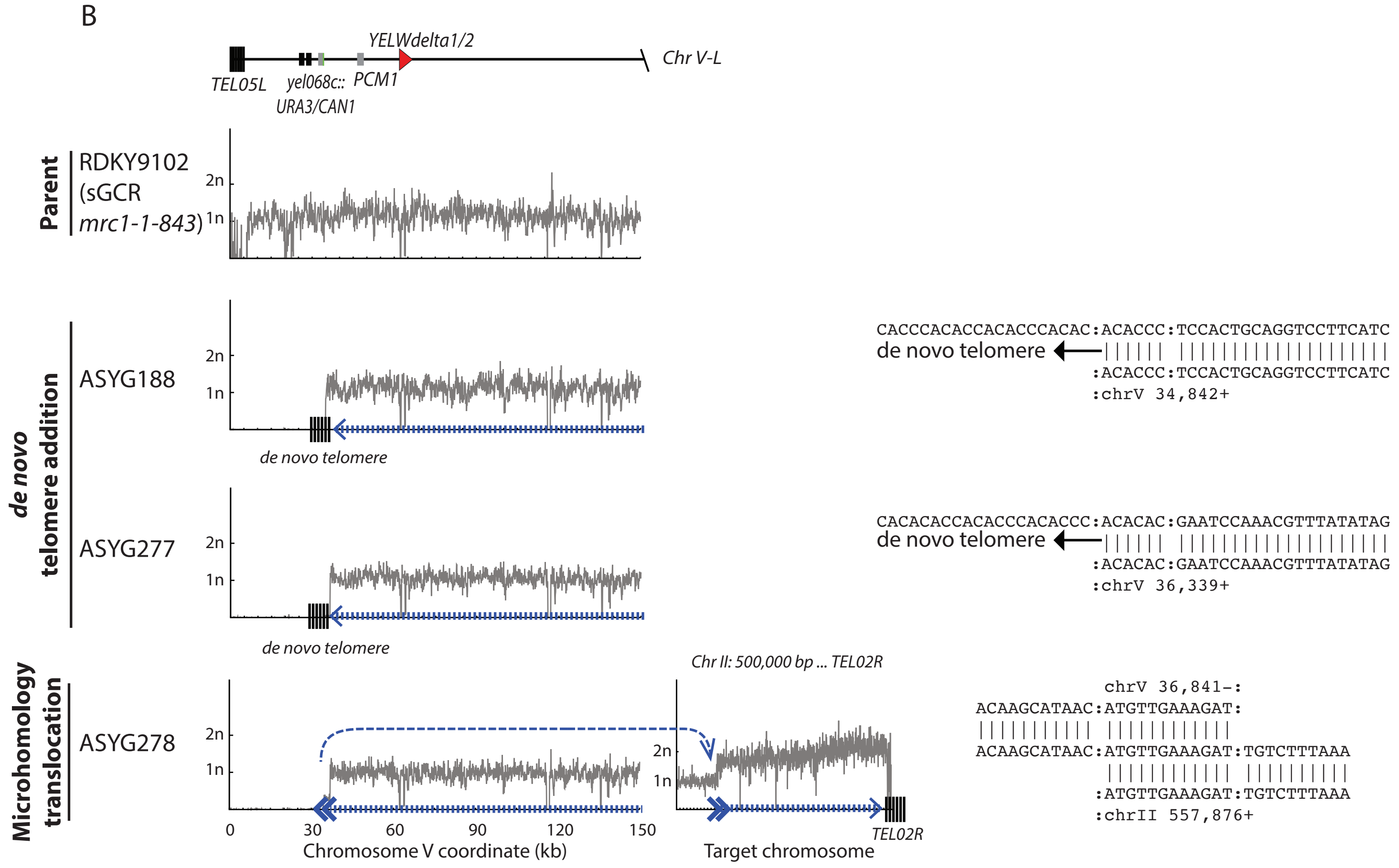

Supplementary Figure 9. Analysis of *mrc1-1-843* GCRs isolated in the sGCR assay. GCRs derived from an *mrc1-1-843* sGCR parent strain (RDKY9102) are depicted as described in Supplementary Fig. 3. Observed rearrangements include homology-mediated translocations (**A**); and *de novo* telomere addition-mediated GCRs and microhomology-mediated translocations (**B**).

Supplementary Figure 10.

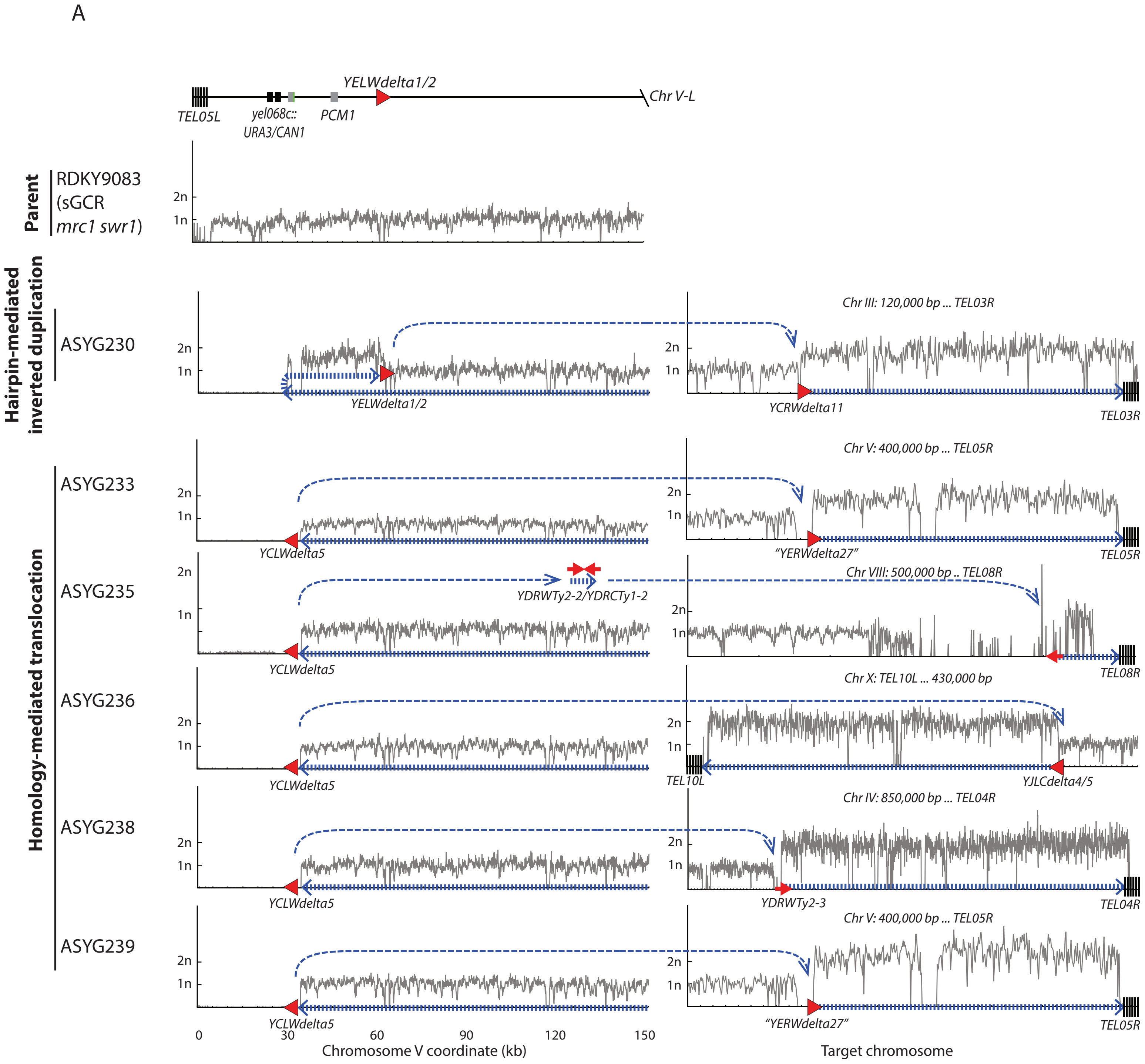

Supplementary Figure 10.

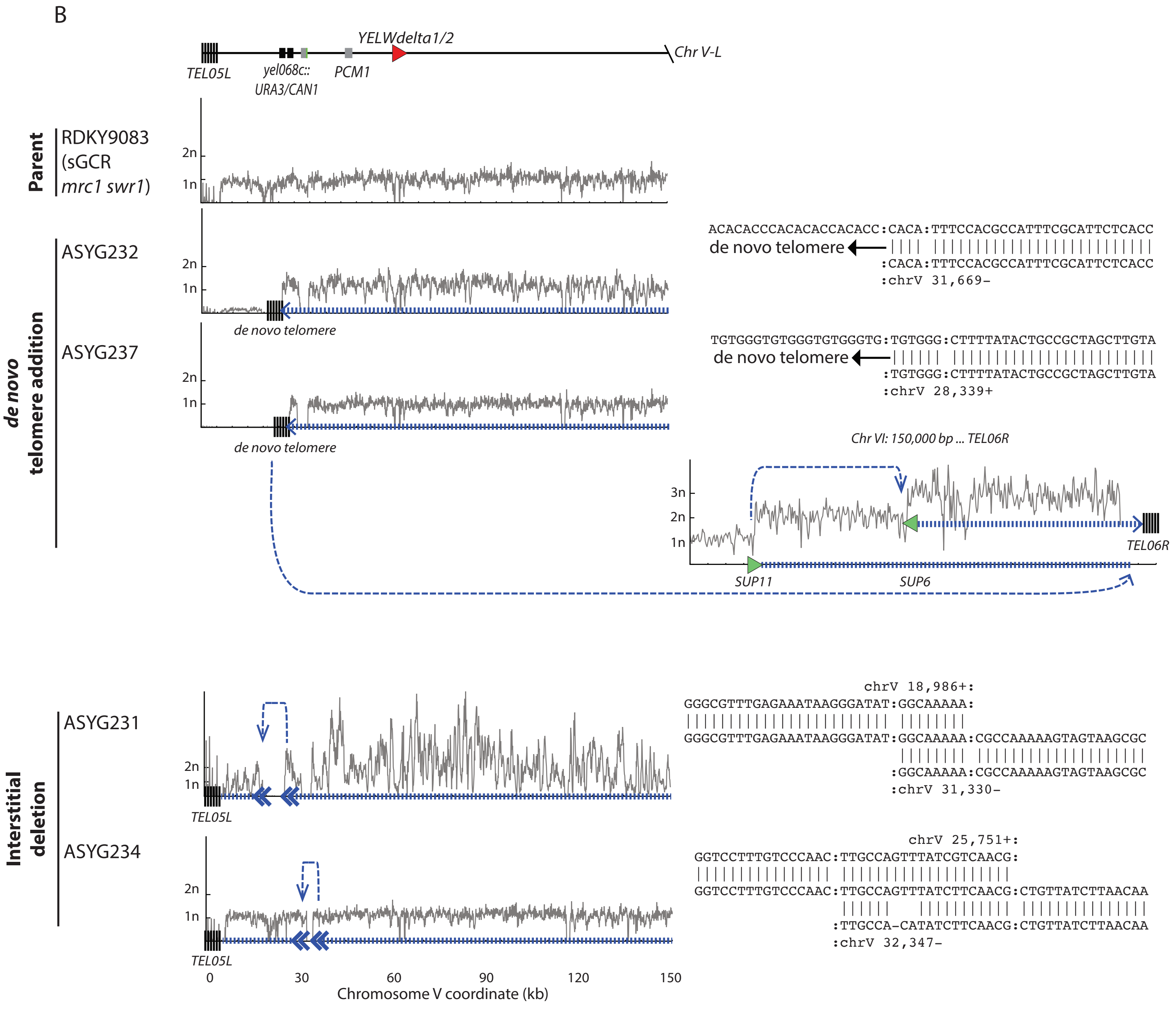

Supplementary Figure 10. Analysis of *mrc1Δ swr1Δ* GCRs isolated in the sGCR assay. GCRs derived from an *mrc1Δ swr1Δ* sGCR parent strain (RDKY9083) are depicted as described in Supplementary Fig. 3. Observed rearrangements include hairpin-mediated inverted duplications and homology-mediated inverted duplications (**A**); and *de novo* telomere addition-mediated GCRs and interstitial deletions (**B**).

Supplementary Figure 11.

A

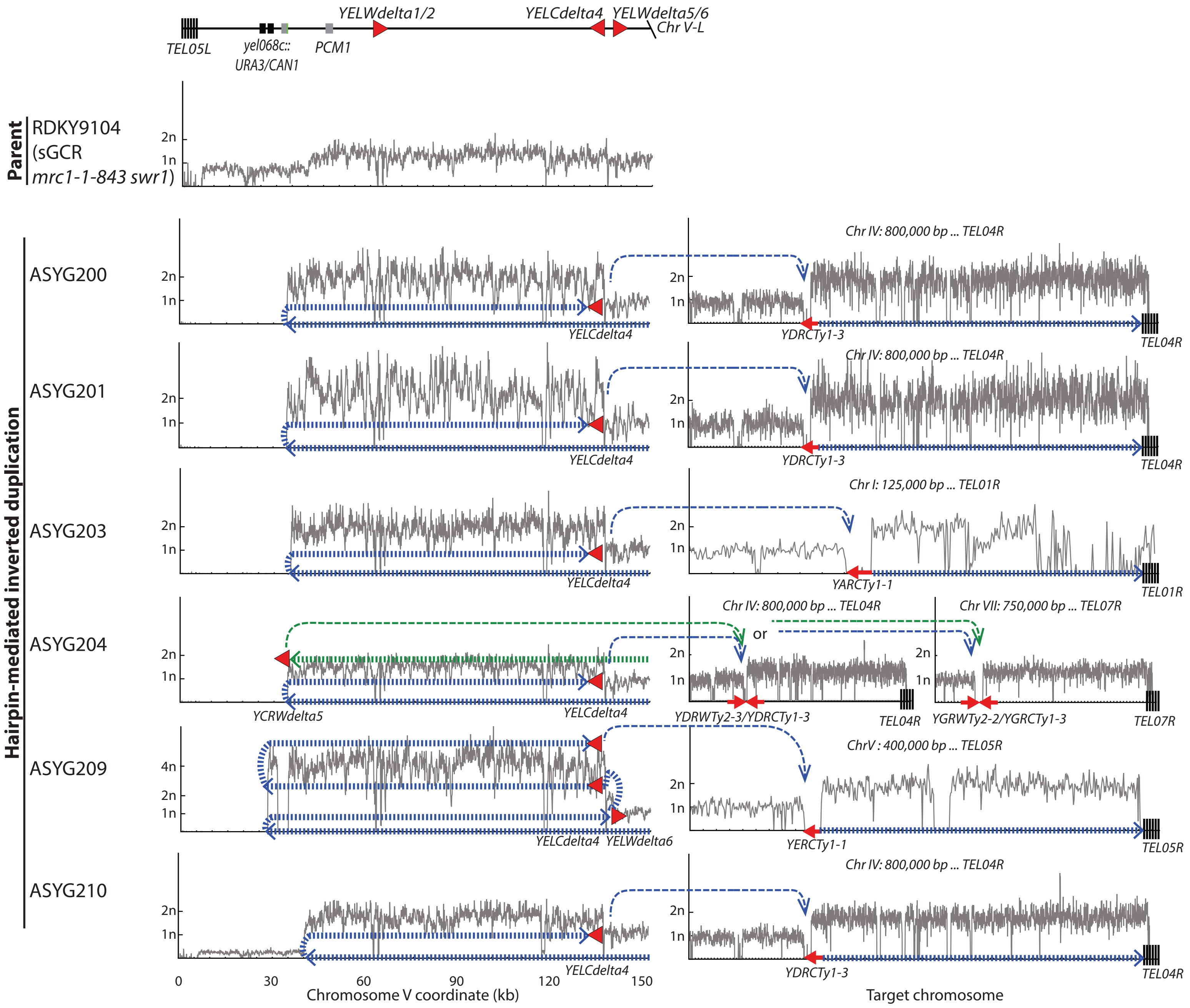

Supplementary Figure 11.

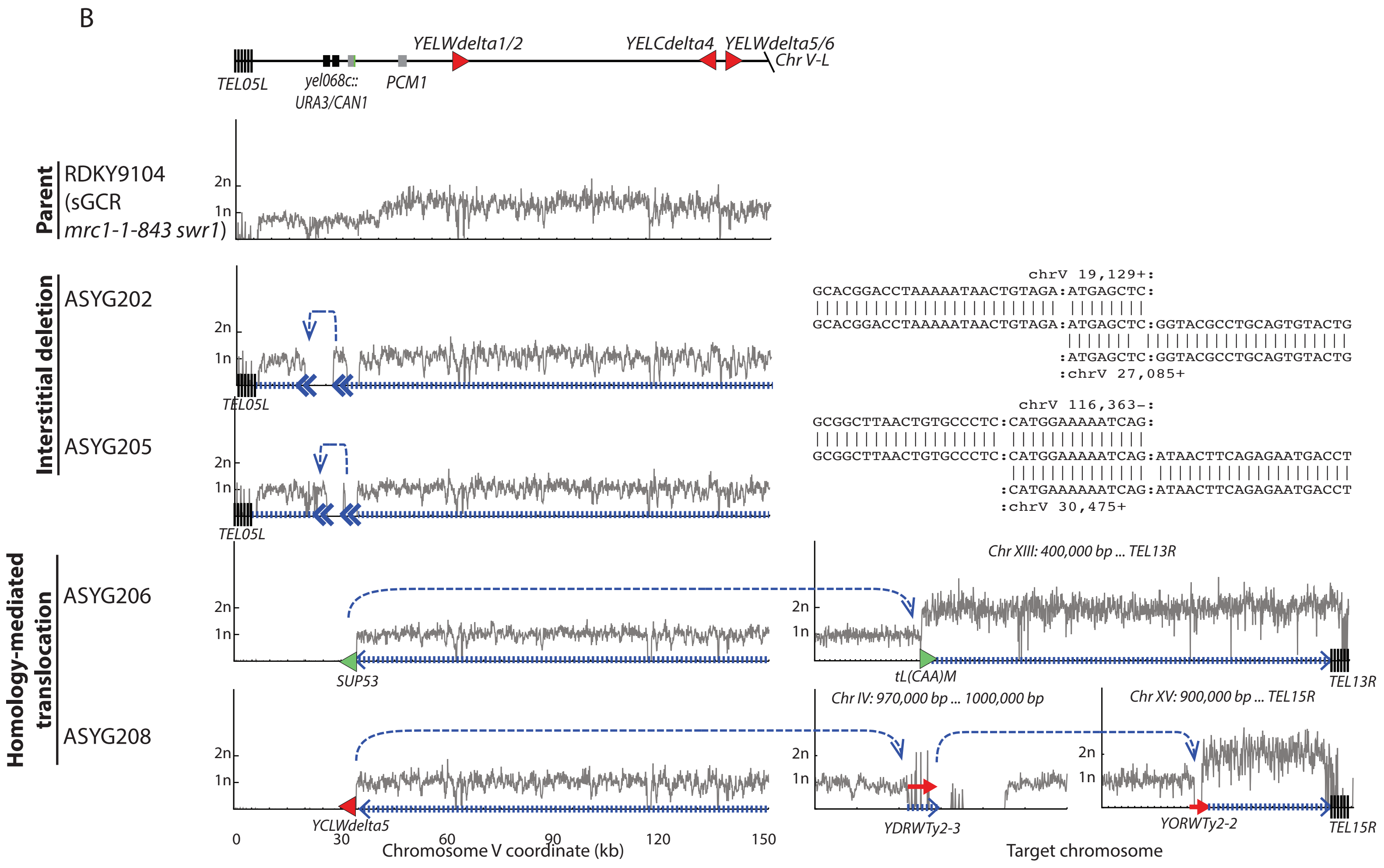

Supplementary Figure 11. Analysis of *mrc1-1-843 swr1Δ* GCRs isolated in the sGCR assay. GCRs derived from an *mrc1-1-843 swr1Δ* sGCR parent strain (RDKY9104) are depicted as described in Supplementary Fig. 3. Observed rearrangements include hairpin-mediated inverted duplications (**A**); and interstitial deletions and homology-mediated translocations (**B**).

Supplementary Figure 12A

ASYG203 (sGCR *mrc1-C14 swr1*)

```
                                                chrV 35,312-:
GAAGGAGCGTATATGTTAGAAAATTGGAAAATATCTGATAATG:TAACA:
||||||||||||||||||||||||||||||||||||||||||| |||||
GAAGGAGCGTATATGTTAGAAAATTGGAAAATATCTGATAATG:TAACA:TATACGCTCCTTCTTCATTAATTAGGAATTTTTTGACTG
                                             ||||| |||||||||||||||||||||||||||||||||||||||
                                            :TAACA:TATACGCTCCTTCTTCATTAATTAGGAATTTTTTGACTG
                                            :chrV 35,342+
```

Potential mechanism of inversion formation

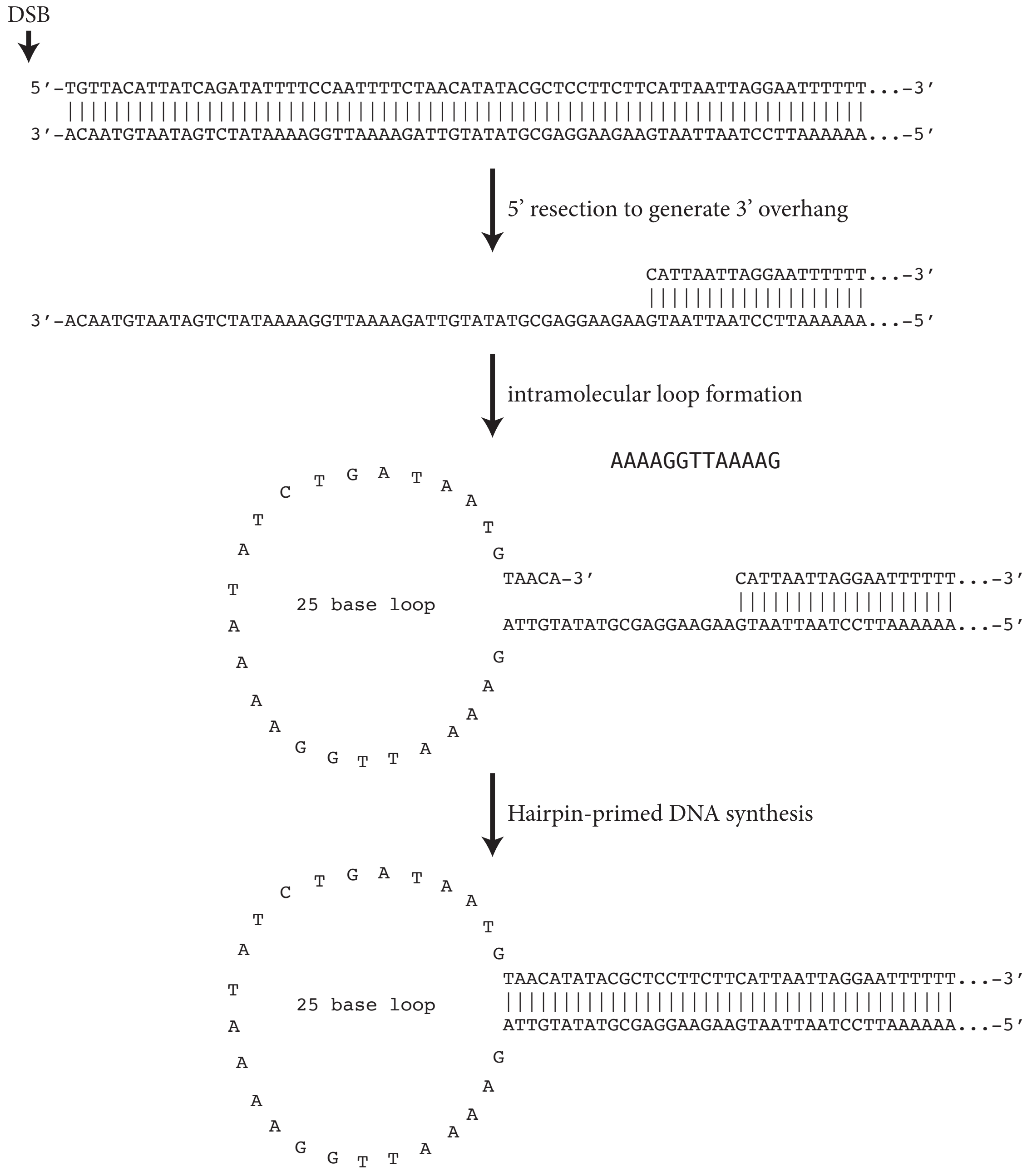

Supplementary Figure 12B

ASYG176
(sGCR *swr1*)

```
                             NAT 125+:
CCTTCACCACCGACACCGTCTTCCGCGT:CACCGCCACCGG:
|||||||||||||||||||||||||||| ||||||||||||
CCTTCACCACCGACACCGTCTTCCGCGT:CACCGCCACCGG:CATCATCATCGTAGAGATACTTG
                             |||||| ||||| |||||||||||||||||||||||
                            :CACCGCGACCGG:CATCATCATCGTAGAGATACTTG
                            :chrV 40,999+
```

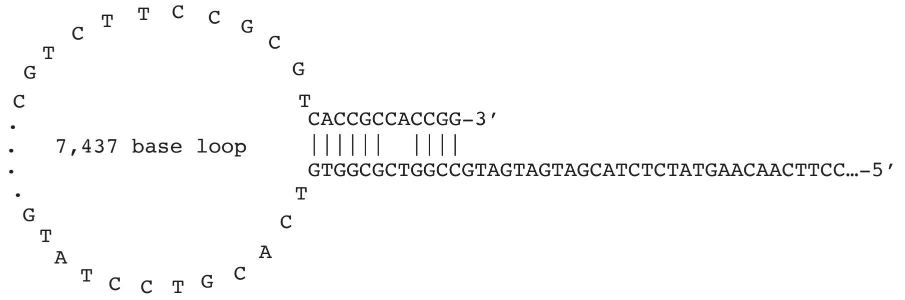

ASYG200
(sGCR *mrc1-C14 swr1*)

```
                                  NAT 166+:
ACGGCTTCACCCTGCGGGAGGTGCCGGTGGACC:CGCCCC:
||||||||||||||||||||||||||||||||| ||||||
ACGGCTTCACCCTGCGGGAGGTGCCGGTGGACC:CGCCCC:GCTCGCGGGCGAACTCCGTCGCGAGCCCCATCAACGCGCGCCCGA
                                  |||||| |||||| ||||||||||||||||||||||||||||||||||||||
                                 :CGCCCC:GCTCGCCGGCGAACTCCGTCGCGAGCCCCATCAACGCGCGCCCGA
                                 :NAT 415-
```

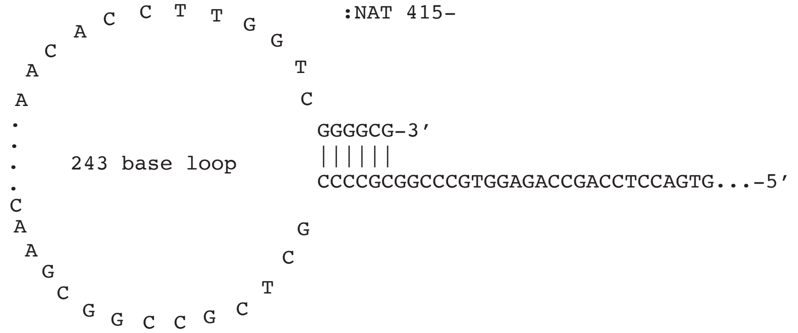

ASYG201
(sGCR *mrc1-C14 swr1*)

```
                              NAT 154+:
ACCGGGGACGGCTTCACCCTGCGGGAGGT:GCCGG:
||||||||||||||||||||||||||||| |||||
ACCGGGGACGGCTTCACCCTGCGGGAGGT:GCCGG:CGGTTCCAGCCGGAGTACGAGACGACCACGAAGCCCGCCAGGTCGCCG
                              ||||| ||||||||  | |||||||||| |||||||||||||||||||||||||
                             :GCCGG:CGGTTCCACGCCGAGTACGAGATGACCACGAAGCCCGCCAGGTCGCCG
                             :NAT 316-
```

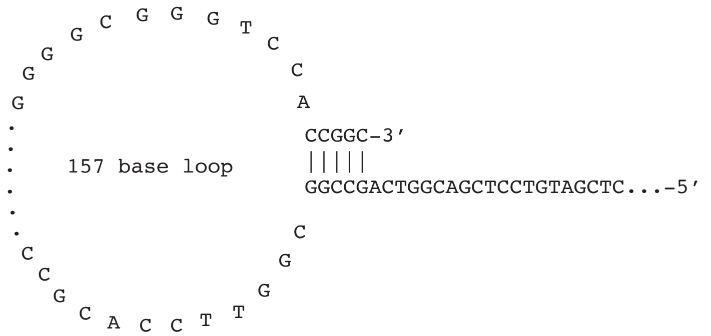

Supplementary Figure 12C

ASYG203
(**sGCR** *mrc1-C14 swr1*)

```
                               chrV 35,312-:
GAAGGAGCGTATATGTTAGAAAATTGGAAAATATCTGATAATG:TAACA:
||||||||||||||||||||||||||||||||||||||||||| |||||
GAAGGAGCGTATATGTTAGAAAATTGGAAAATATCTGATAATG:TAACA:TATACGCTCCTTCTTCATTAATTAGGAATTTTTTGACTG
                                            ||||| |||||||||||||||||||||||||||||||||||||||
                                           :TAACA:TATACGCTCCTTCTTCATTAATTAGGAATTTTTTGACTG
                                           :chrV 35,342+
```

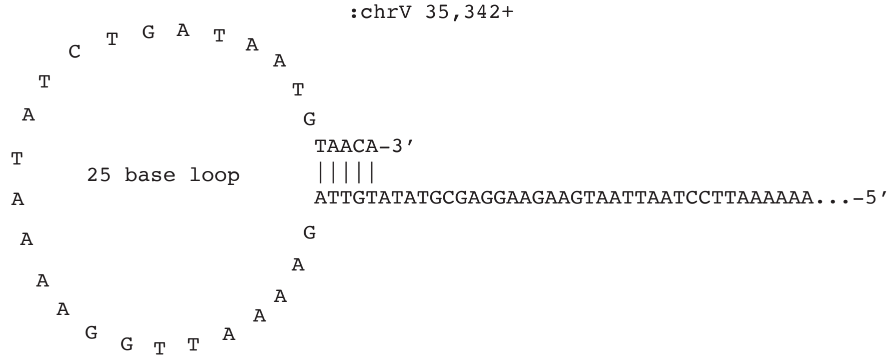

ASYG204
(**sGCR** ***mrc1-C14 swr1***)

```
                                   NAT 204+:
CCCTGACCAAGGTGTTCCCCGACGACGAATCGGA:CGACGAA:
|||||||||||||||||||||||||||||||||| |||||||
CCCTGACCAAGGTGTTCCCCGACGACGAATCGGA:CGACGAA:CGTCCGGGAGTCCGGGTCGCCGTCCTCCCCGTCGTCCG
                                   ||||||| ||||||||||||||||||||||||||||||||||||||
                                  :CGACGAA:CGTCCGGGAGTCCGGGTCGCCGTCCTCCCCGTCGTCCG
                                  :NAT 250-
```

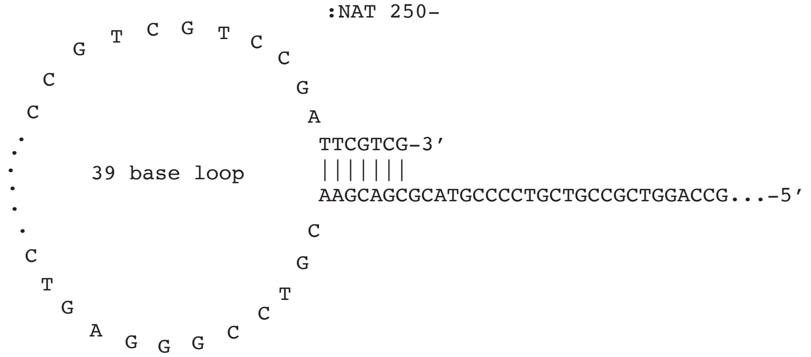

ASYG209
(**sGCR** ***mrc1-C14 swr1***)

```
                                                  chrV 27,718-:
AGAAGATGTGGTTAGCGTTGGCTTGACATCGACCTTGGCTCCTACTTCC:TTTTCCAGCATCT:
||||||||||||||||||||||||||||||||||||||||||||||||| |||||||||||||
AGAAGAGGTGGTTAGCGTTGGCTTGACATCGACCTTGGCTCCTACTTCC:TTTTCCAGCACCT:TCTTACATTGAATTGATAGATCCAG
                                                  ||||||||||||| |||||||||||||||||||||||||
                                                 :TTCTCCAGCACCT:TCTTACATTGAATTGATAGATCCAG
                                                 :chrV 27,776+
```

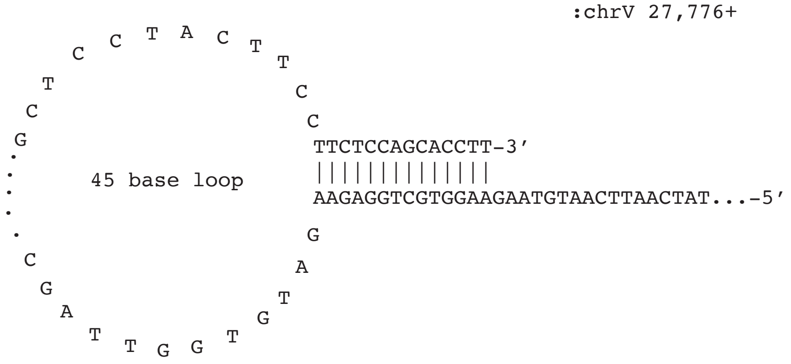

Supplementary Figure 12D

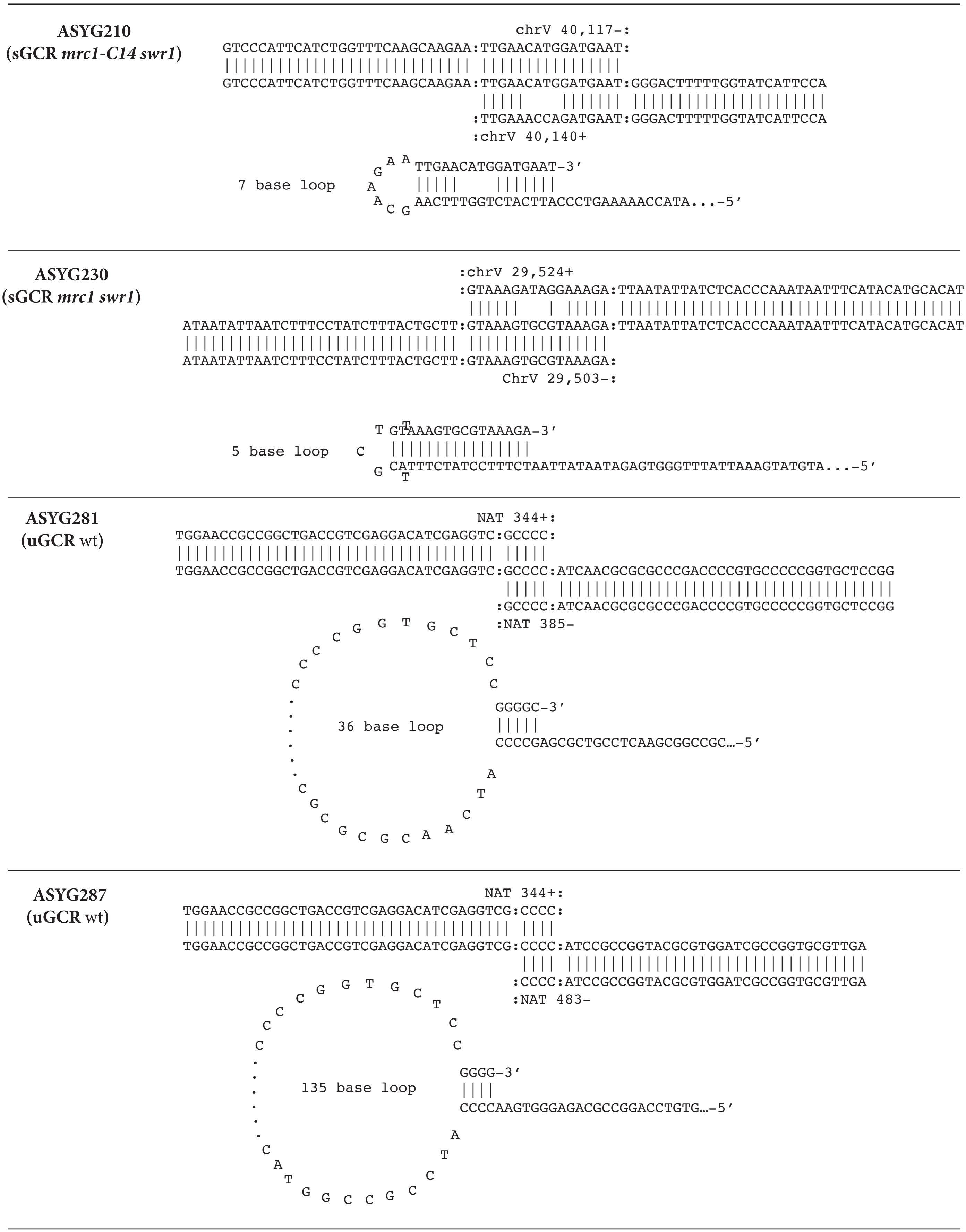

Supplementary Figure 12E

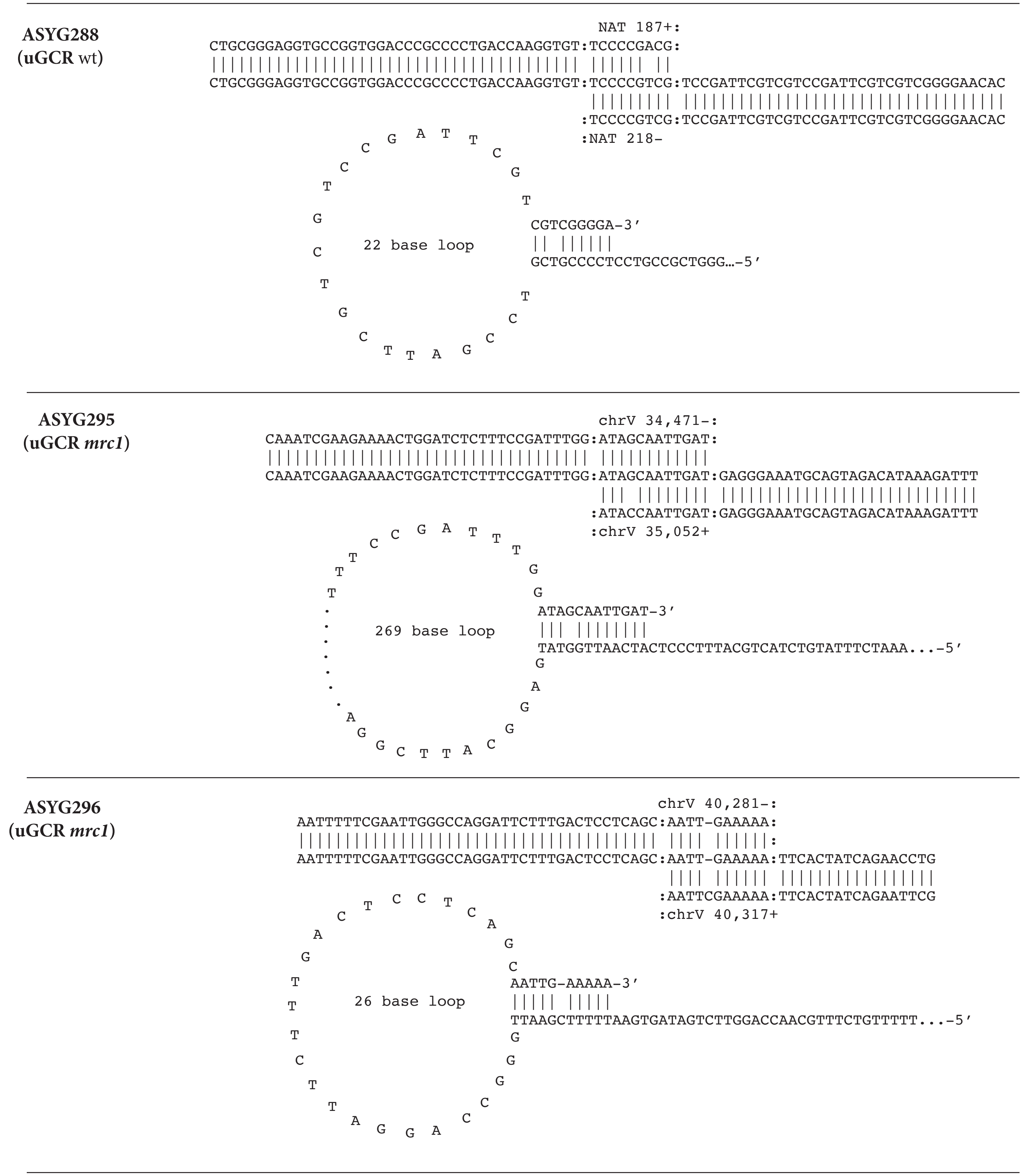

Supplementary Figure 12F

ASYG300
(uGCR *mrc1*)

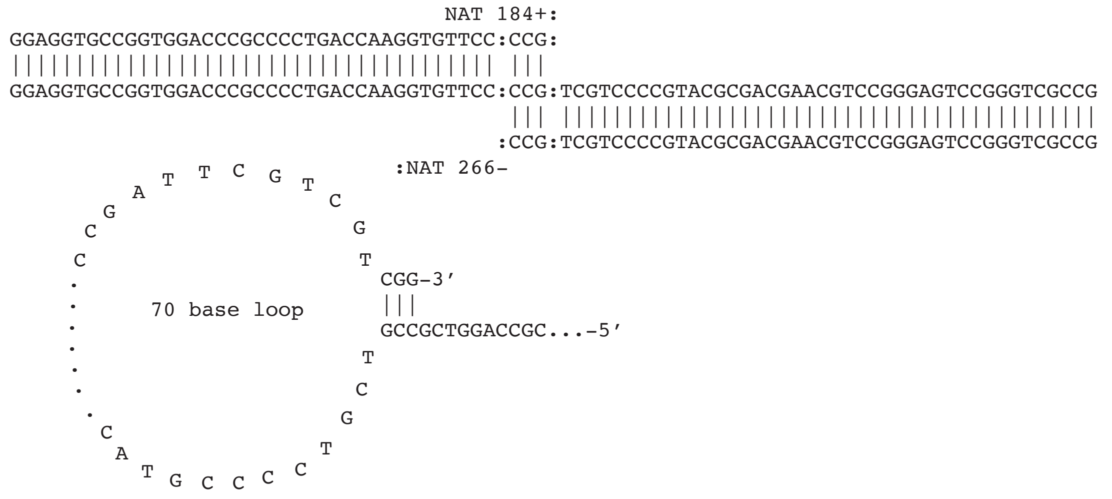

ASYG301
(uGCR *mrc1*)

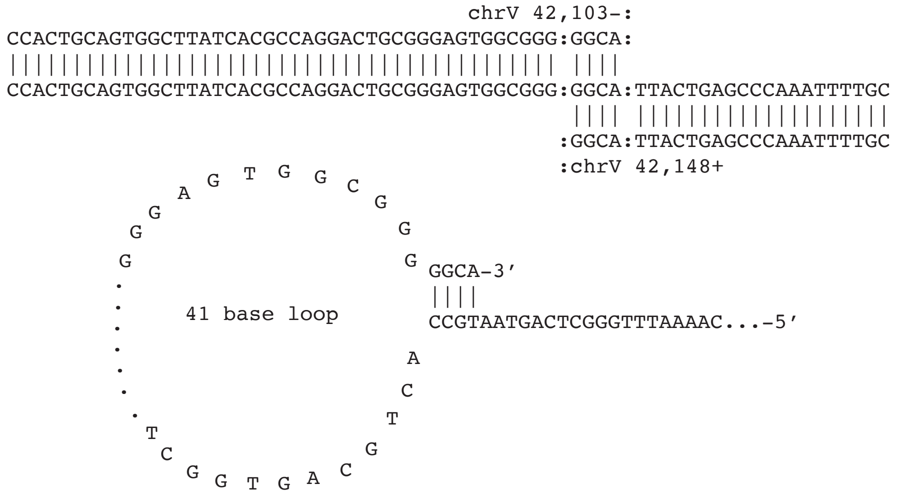

ASYG302
(uGCR *mrc1*)

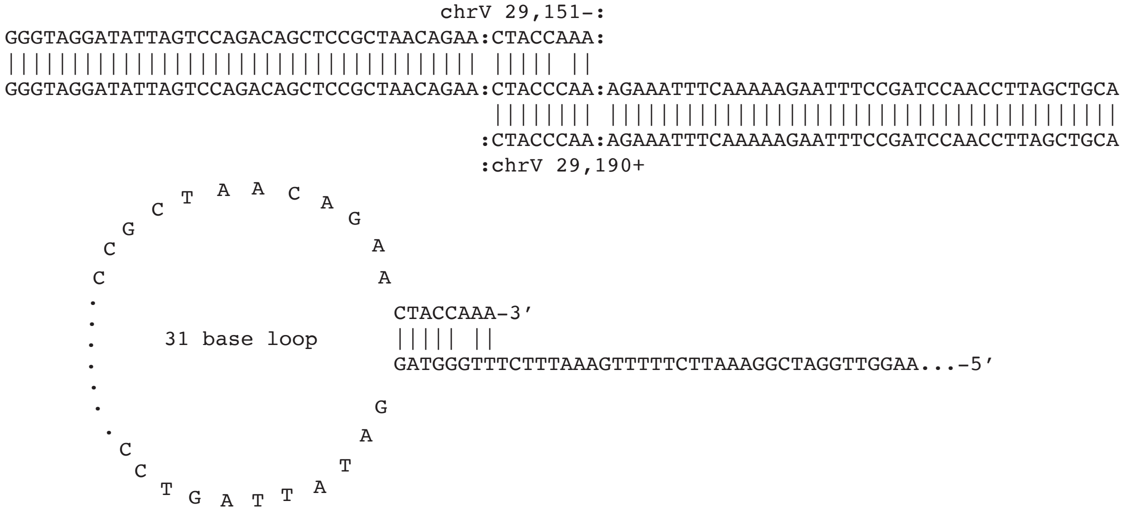

Supplementary Figure 12G

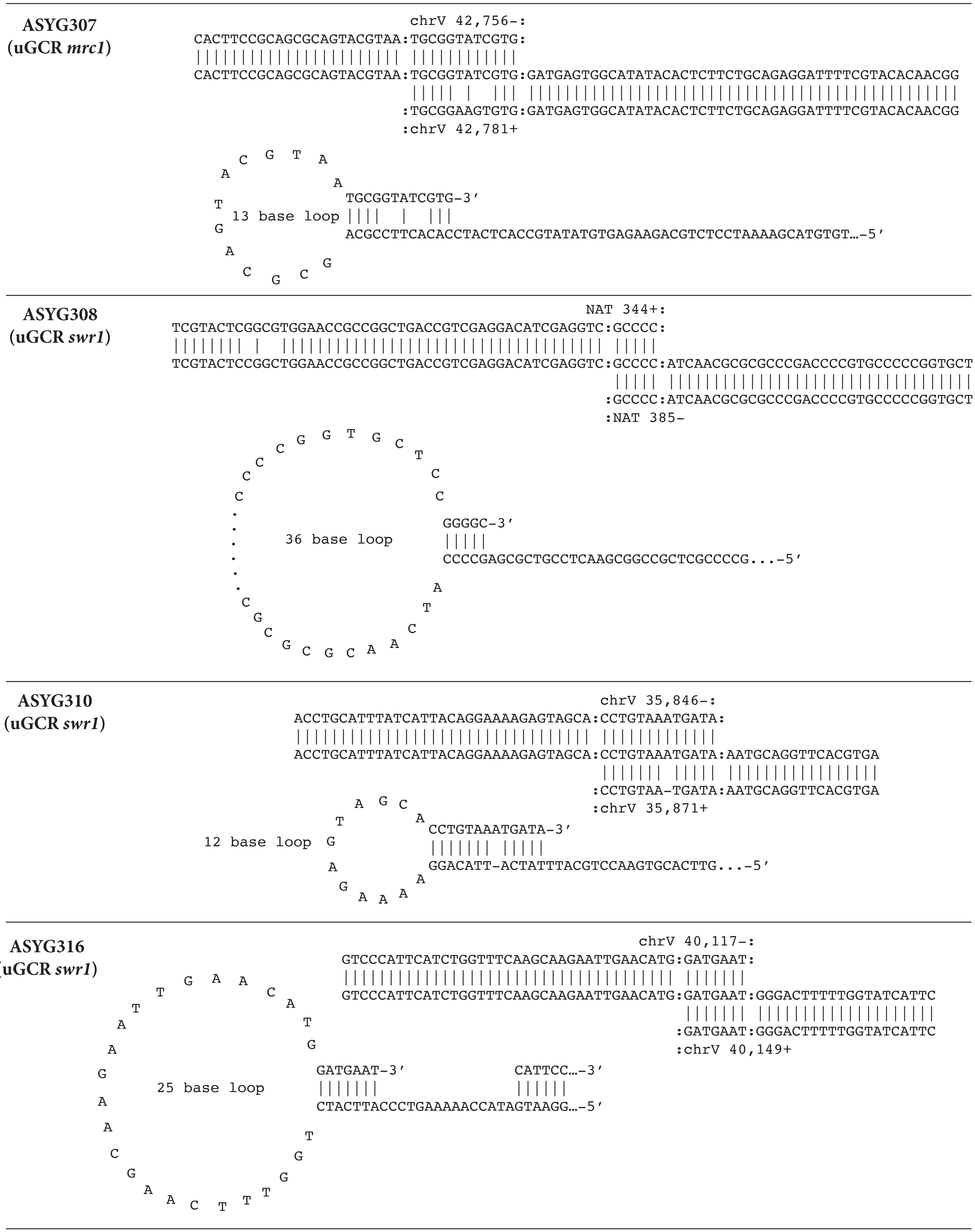

Supplementary Figure 12H

ASYG317
(uGCR *swr1*)

```
                                      NAT 104+:
CGAGGCCATCGAGGCACTGGATGGGTCCTTCACCA:CCGACACCGT:
||||||||||||||||||||||||||||||||||| ||||| ||||
CGAGGCCATCGAGGCACTGGATGGGTCCTTCACCA:CCGACCCCGT:GCCCCCGGTGCTCCGGGGCGACCTCGATGTCCTCGA
                                    |||||||||| ||||||||||||||||||||||||||||||||||||
                                   :CCGACCCCGT:GCCCCCGGTGCTCCGGGGCGACCTCGATGTCCTCGA
                                   :NAT 368-
```

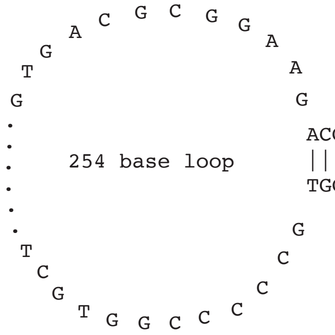


```
ACGGTGTCGG-3’
|||| |||||
TGCCCCAGCCCGCGCGCAACTACCCCGAGCGCTGCCTCAA…-5’
```

ASYG325
(uGCR *mrc1 swr1*)

```
                                      chrV 39,120-:
ACTGGTATATGGTATGACGTCAACAGGTAAAA:CATATACAATGACGG:
|||||||||||||||||||||||||||||||| |||||||||||||||
ACTGGTATATGGTATGACGTCAACAGGTAAAA:CATATACAATGACGG:TGCAATTGTAACCTTTAATGAAATCCTGGAATAATGGGC
                                 ||||||| |  |||| |||||||||||||||||||||||||||||||||||||||
                                :CATATACCAGTACGG:TGCAATTGTAACCTTTAATGAAATCCTGGAATAATGGGC
                                :chrV 39,156+
```

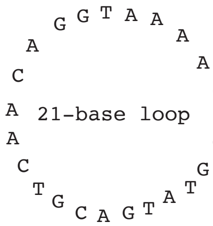


```
CATATACAATGACGG-3’
||||||| |  ||||
GTATATGGTCATGCCACGTTAACATTGGAAATTAC...-5’
```

ASYG328
(uGCR *mrc1 swr1*)

```
                                  chrV 35,846-:
AACCTGCATTTATCATTACAGGAAAAGAGTAGCA:CCTGTAAATGATA:
|||||||||||||||||||||||||||||||||| |||||||||||||
AACCTGCATTTATCATTACAGGAAAAGAGTAGCA:CCTGTAAATGATA:AATGCAGGTTCACGTGAACCA
                                   ||||||| ||||| |||||||||||||||||||||
                                  :CCTGTAA-TGATA:AATGCAGGTTCACGTGAACCA
                                  :chrV 35,871+
```

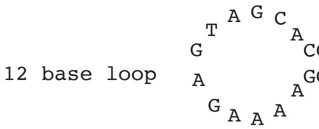


```
CCTGTAAATGATA-3’
GGACATT-ACTATTTACGTCCAAGTGCACTTGGT...-5’
```

ASYG477
(uGCR *mrc1*)

```
                                chrV 35,846-:
CCTGCATTTATCATTACAGGAAAAGAGTAGCA:CCTGTAAATGATA:
|||||||||||||||||||||||||||||||| |||||||||||||
CCTGCATTTATCATTACAGGAAAAGAGTAGCA:CCTGTAAATGATA:AATGCAGGTTCACGTGAACCAAAAGAAATGACACCAATAAT
                                 ||||||| ||||| |||||||||||||||||||||||||||||||||||||||||
                                :CCTGTAA-TGATA:AATGCAGGTTCACGTGAACCAAAAGAAATGACACCAATAAT
                                :chrV 35,871+
```

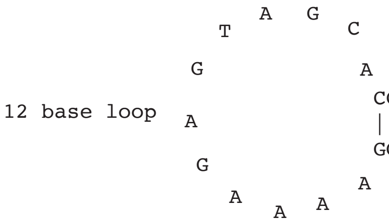


```
CCTGTAAATGATAA-3’
||||||| ||||||
GGACATT-ACTATTTACGTCCAAGTGCACTTGGTTTTCTTTACTGTGGTTATTA...-5’
```

Supplementary Figure 12I

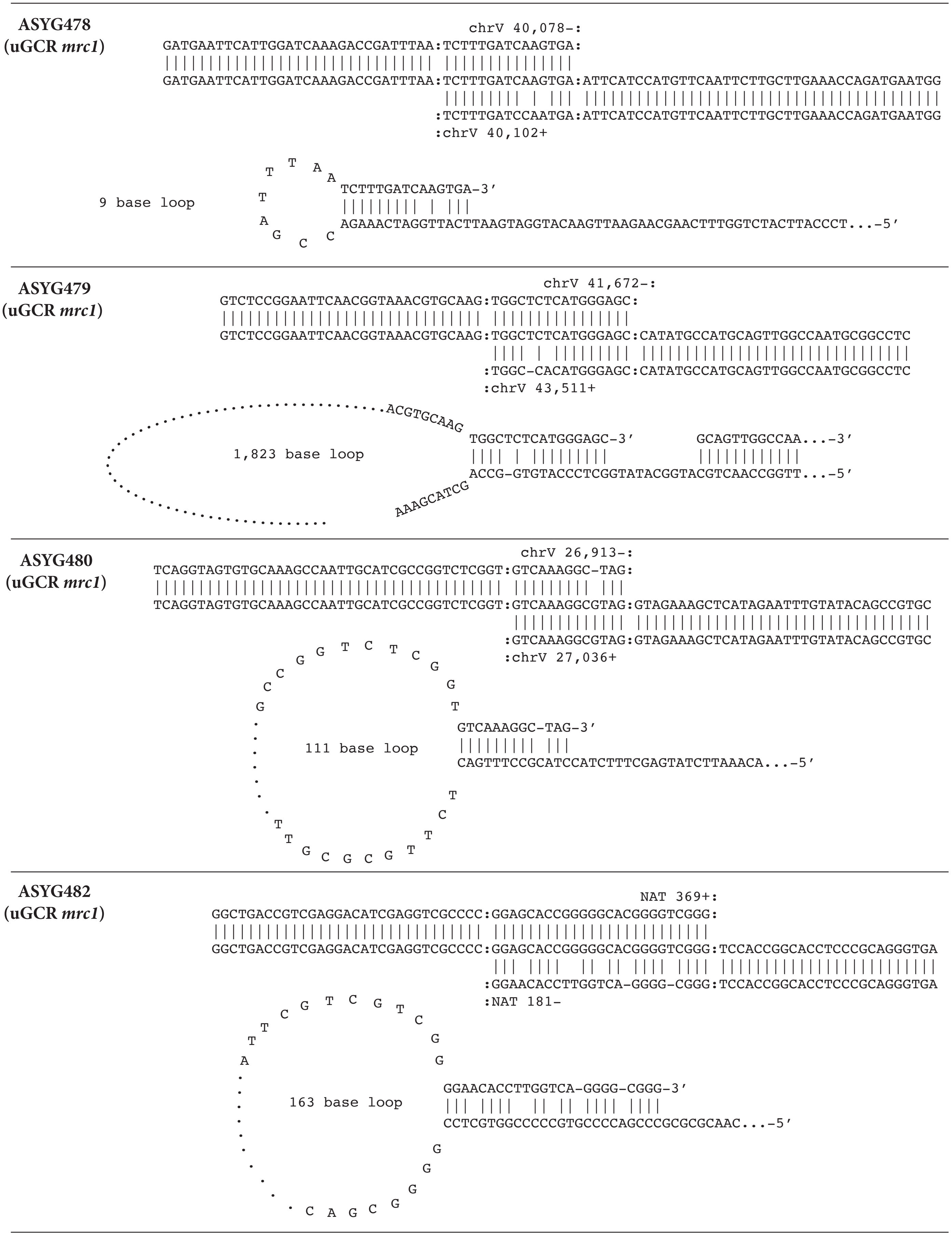

Supplementary Figure 12J

ASYG483
(uGCR *mrc1*)

```
                                                   NAT 276+:
CCCGGACTCCCGGACGTTCGTCGCGTACGGGGACGACGGCGACCT:GGCG:
||||||||||||||||||||||||||||||||||||||||||||| ||||
CCCGGACTCCCGGACGTTCGTCGCGTACGGGGACGACGGCGACCT:GGCG:ACCTCGATGTCCTCGACGGTCAGCCGGCGGTTC
                                               |||| |||||||||||||||||||||||||||||||||
                                              :GGCG:ACCTCGATGTCCTCGACGGTCAGCCGGCGGTTC
                                              :NAT 342-
```

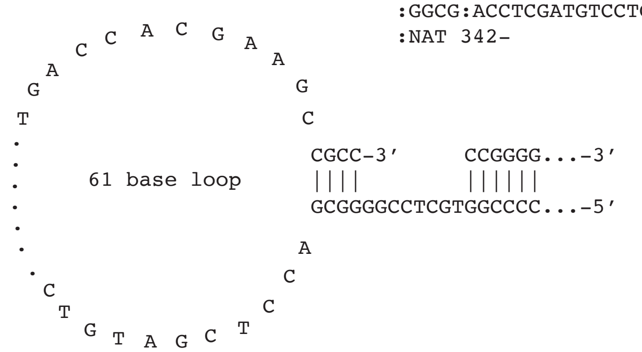

ASYG485
(uGCR *swr1*)

```
                                             NAT 190+:
GAGGTGCCGGTGGACCCGCCCCTGACCAAGGTGTTCCCCGA:CGACG:
||||||||||||||||||||||||||||||||||||||||| |||||
GAGGTGCCGGTGGACCCGCCCCTGACCAAGGTGTTCCCCGA:CGACG:GTCAGCCGGCGGTTCCAGCCGGAGTACGAGACGACCACGAA
                                          ||||| ||||||||||||||||  | |||||||||| |||||||||
                                         :CGACG:GTCAGCCGGCGGTTCCACGCCGAGTACGAGATGACCACGAA
                                         :NAT 325-
```

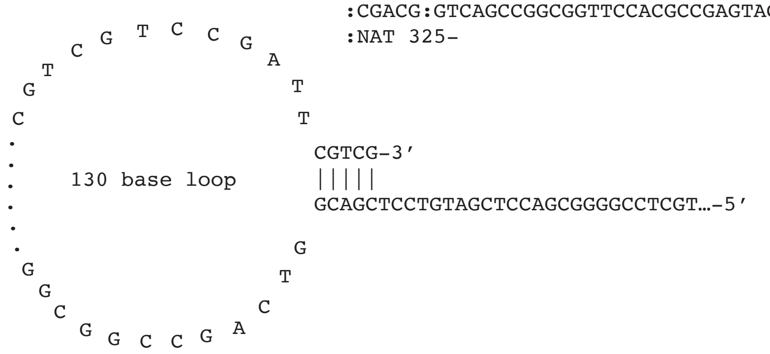

ASYG490
(uGCR *swr1*)

```
                     NAT 61+:
ACCGCACCAGTGTCCCGGGG:GACGCCG:
|||||||||||||||||||| ||| |||
ACCGCACCAGTGTCCCGGGG:GACCCCG:TGCCCCCGGTGCTCCGGGGCGACCTCGATGTCCTCGACGGTCAGCCGGCGGTT
                      ||||||| ||||||||||||||||||||||||||||||||||||||||||||||||||||
                     :GACCCCG:TGCCCCCGGTGCTCCGGGGCGACCTCGATGTCCTCGACGGTCAGCCGGCGGTT
                     :NAT 366-
```

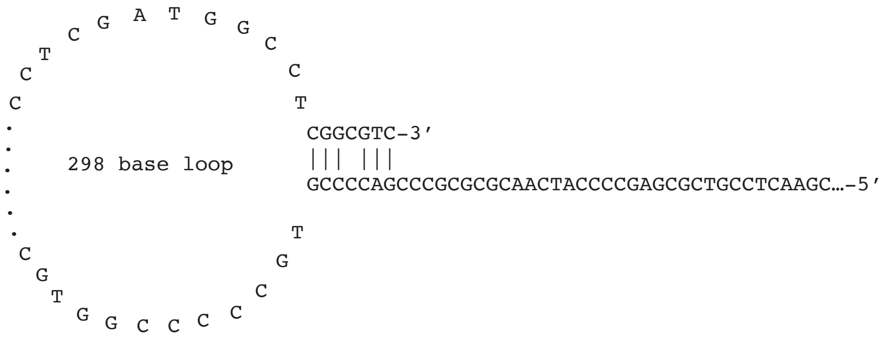

Supplementary Figure 12. Structures of hairpin intermediates in hairpin-mediated inverted duplications. **A**. Potential mechanism for the formation of a hairpin-mediated inverted duplication. **B.-J**. Top for each isolate: junction sequences recovered from NGS of the hairpin-mediated inverted duplication GCRs (middle line) and alignments to the two portions of the reference genome (top and bottom lines). The sequence between the colons corresponds to the microhomology at the junction, which likely corresponds to the annealed position of the hairpin intermediate. Bottom for each isolate: structures of the key hairpin intermediates. Loop sizes are not drawn to scale.

Supplementary Figure 13.

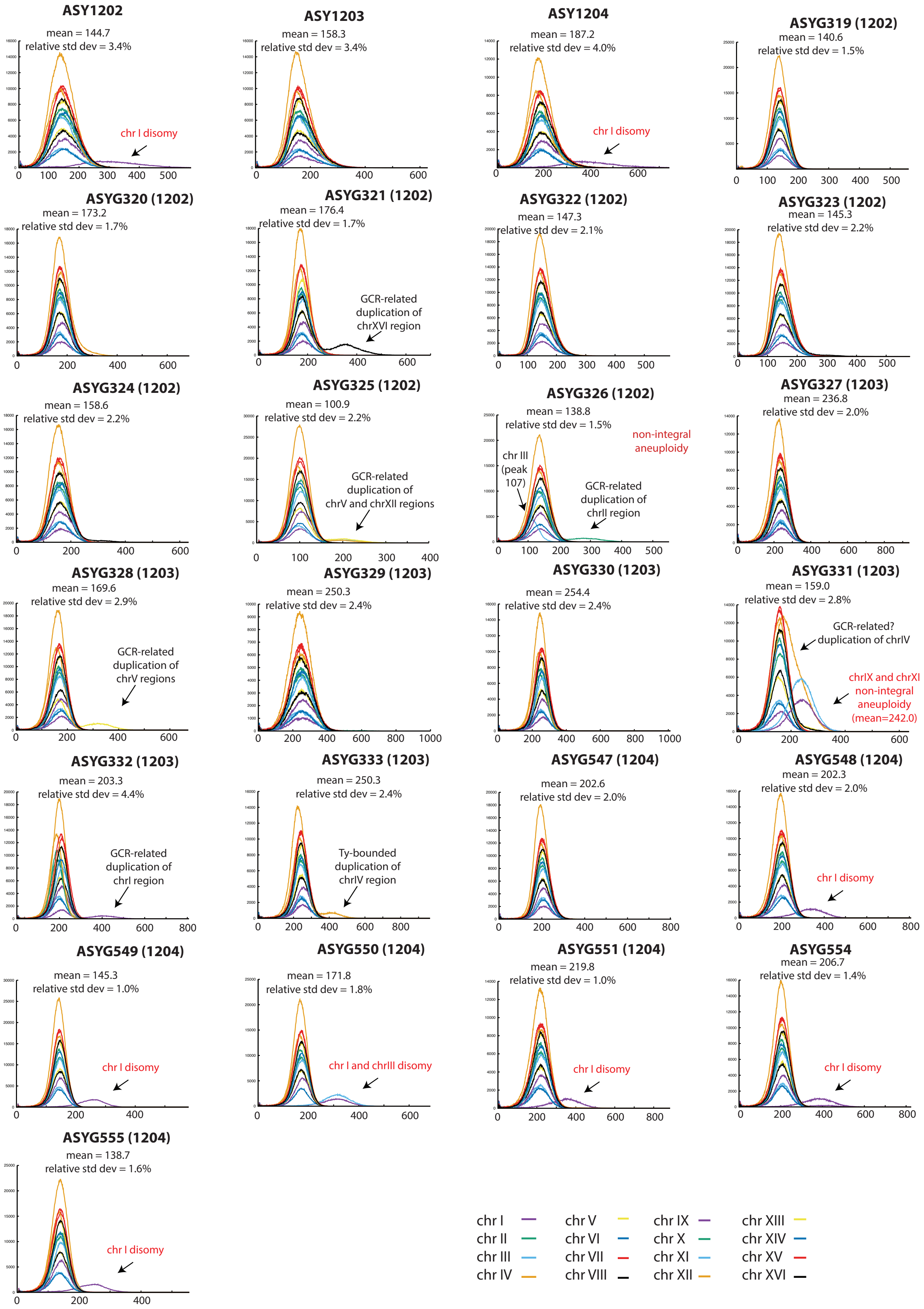

Supplementary Figure 13. Analysis of the aneuploidy in *mrc1Δ swr1Δ* parental strains and GCR-containing isolates. Copy number histogram plots for all chromosomes of the sequenced *mrc1Δ swr1Δ* uGCR parental strains (ASY1202-ASY1204) and the GCR-containing isolates derived from these strains. The parental strain for each isolate is indicated in parentheses. The x-value at the peak position for each plot corresponds to the average number of times base pairs in unique sequences were present in sequence reads. Secondary peaks specified by the arrow and accompanying description indicate copy number alterations in specific chromosomes, which are either GCR-related or due to aneuploidy.

Supplementary Figure 14

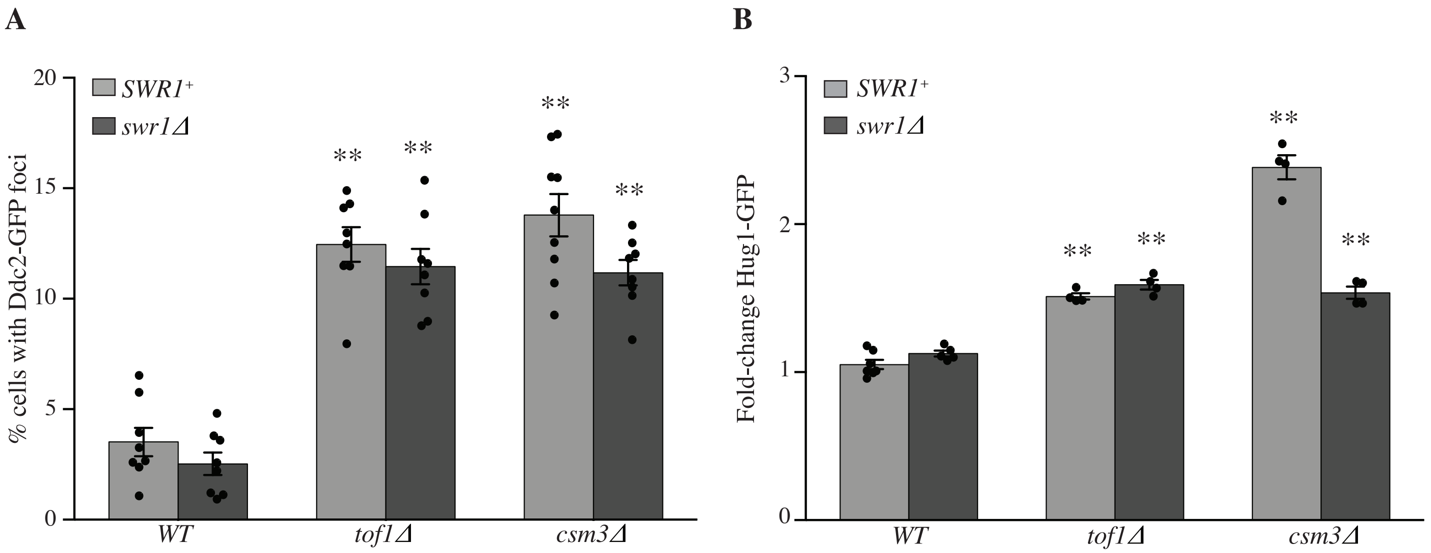

Supplementary Figure 14. Defects in Tof1-Csm3 cause constitutive DNA-damage checkpoint activation. A. Distribution of Ddc2-GFP foci in strains with a *tof1Δ* or *csm3Δ* mutation with or without the *swr1Δ* mutation. **B.** Hug1-GFP induction in strains with a *tof1Δ* or *csm3Δ* mutation with or without the *swr1Δ* mutation, shown as the fold change in the mean Hug1-GFP level measured by FACS. The mean fold changes were calculated from at least 2 independent experiments using at least 2 independent isolates for each strain. Individual observations are shown as dots overlaid on the bar graphs. The error bars represent the standard error of the mean. Asterisks represent significant differences with respect to the wild-type strain as follows: * $p < 0.005$, ** $p < 0.0005$ (2-tailed t-test).

Supplementary Figure 15

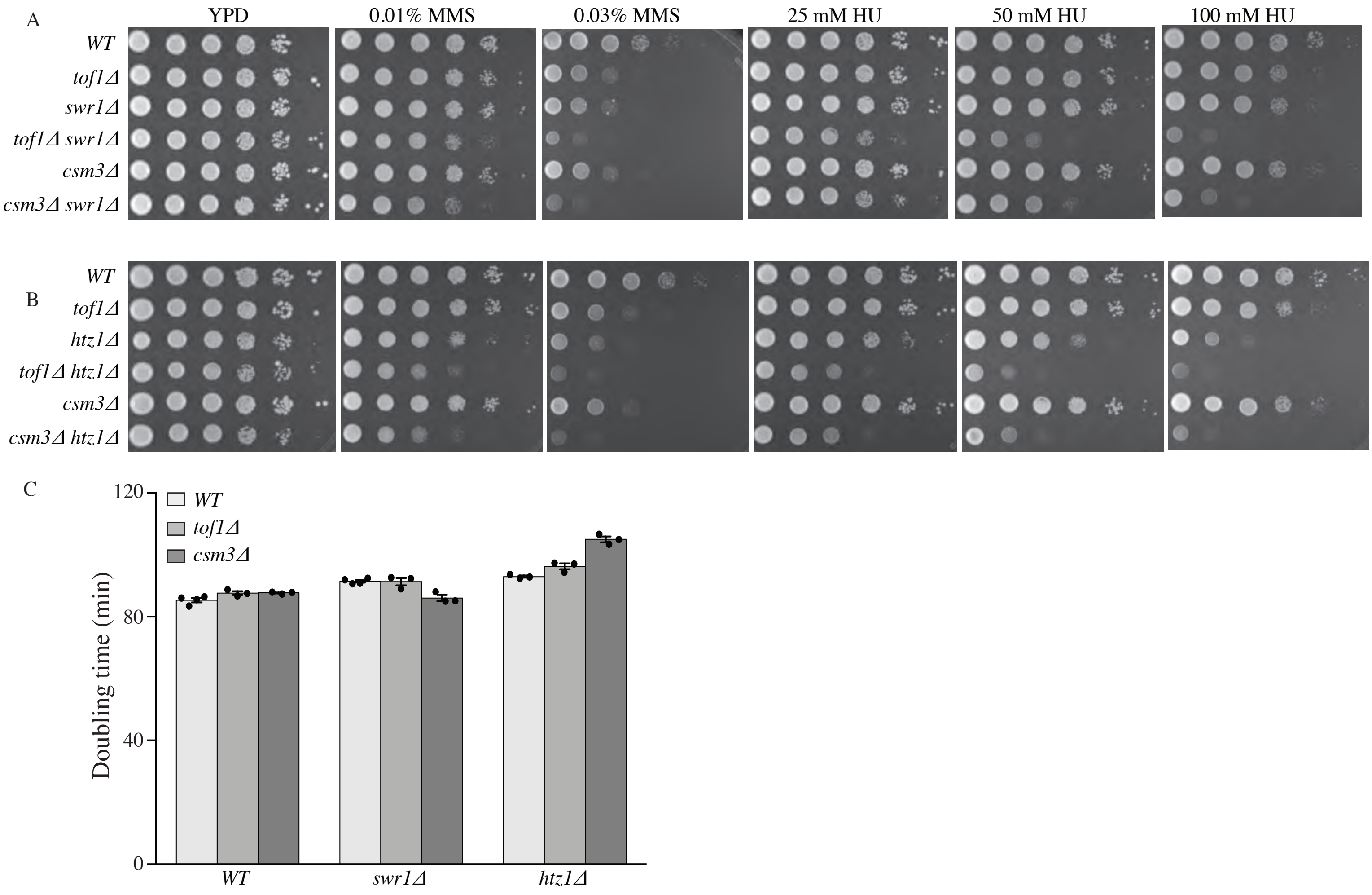

Supplementary Figure 15. Defects in Tof1-Csm3 and SWR-C/Htz1 cause synergistic sensitivity to replication stress. A, B. Ten-fold serial dilutions of wild-type and mutant strains containing the *tof1Δ* or *csm3Δ* mutation with or without the *swr1Δ* mutation (**A**) or *htz1Δ* mutation (**B**) were plated on media containing 0-0.03% MMS or 0-100 mM HU. Plates were imaged after 2 days of growth at 30°C. **C**. Doubling times of the indicated strains in the absence of DNA-damaging agents. Individual observations as shown as dots overlaid on the bar graphs. The error bars represent the standard error of the mean.

Supplementary Figure 16

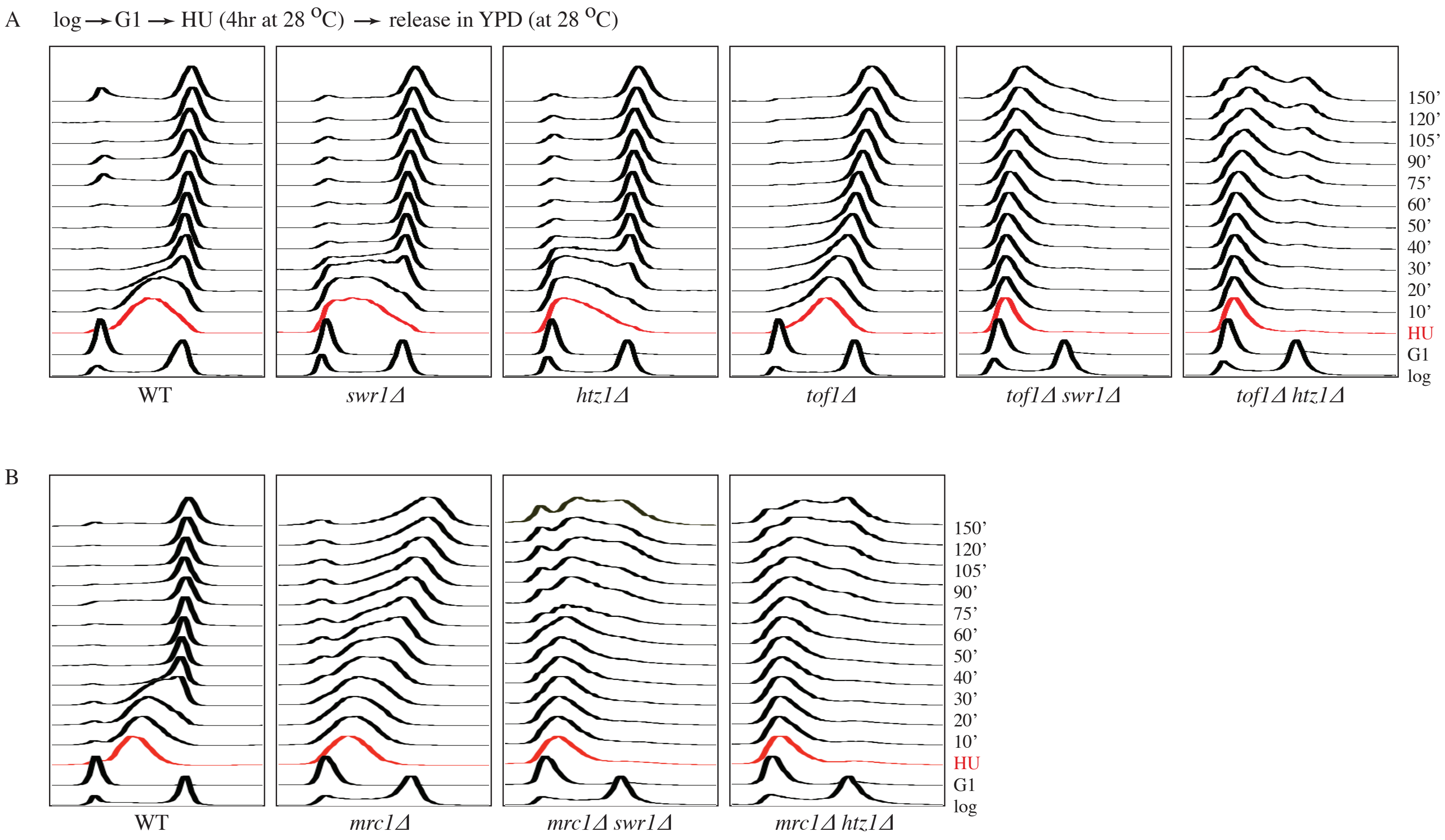

Supplementary Figure 16. S-phase progression in the presence of HU and after transient HU treatment. Logarithmically growing cells of the indicated genotypes were arrested in G1 phase, allowed to replicate in the presence of 200 mM HU for 4 hr and then released in normal media. Samples were taken at the indicated time-points for FACS analysis.

Supplementary Figure 17

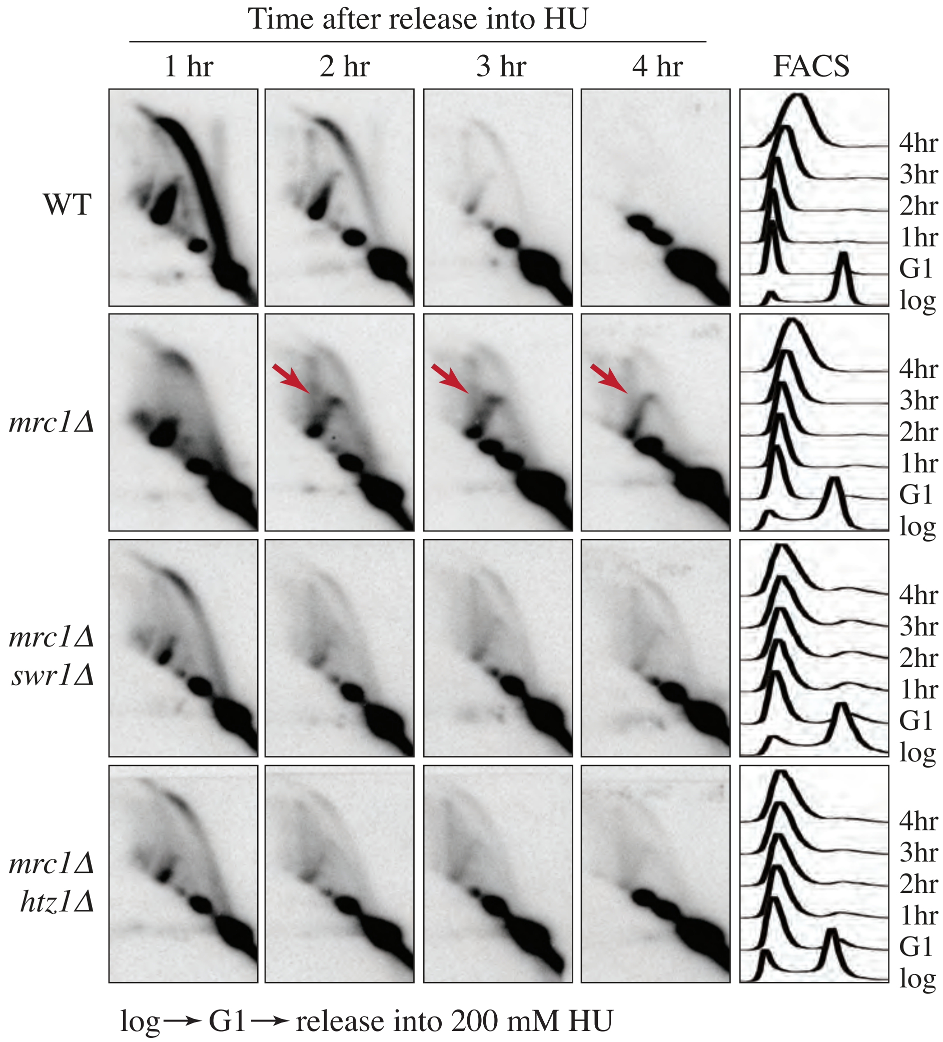

Supplementary Figure 17. Profile of replication intermediates in *mrc1* strains proficient or deficient in the SWR-C/Htz1. 2D gels and FACS profiles of strains of the indicated genotype, replicating in the presence of 200 mM HU. The profile of replication intermediates revealed by 2D gels corresponds to the ARS305 region, as in Fig. 5A.

Supplementary Figure 18

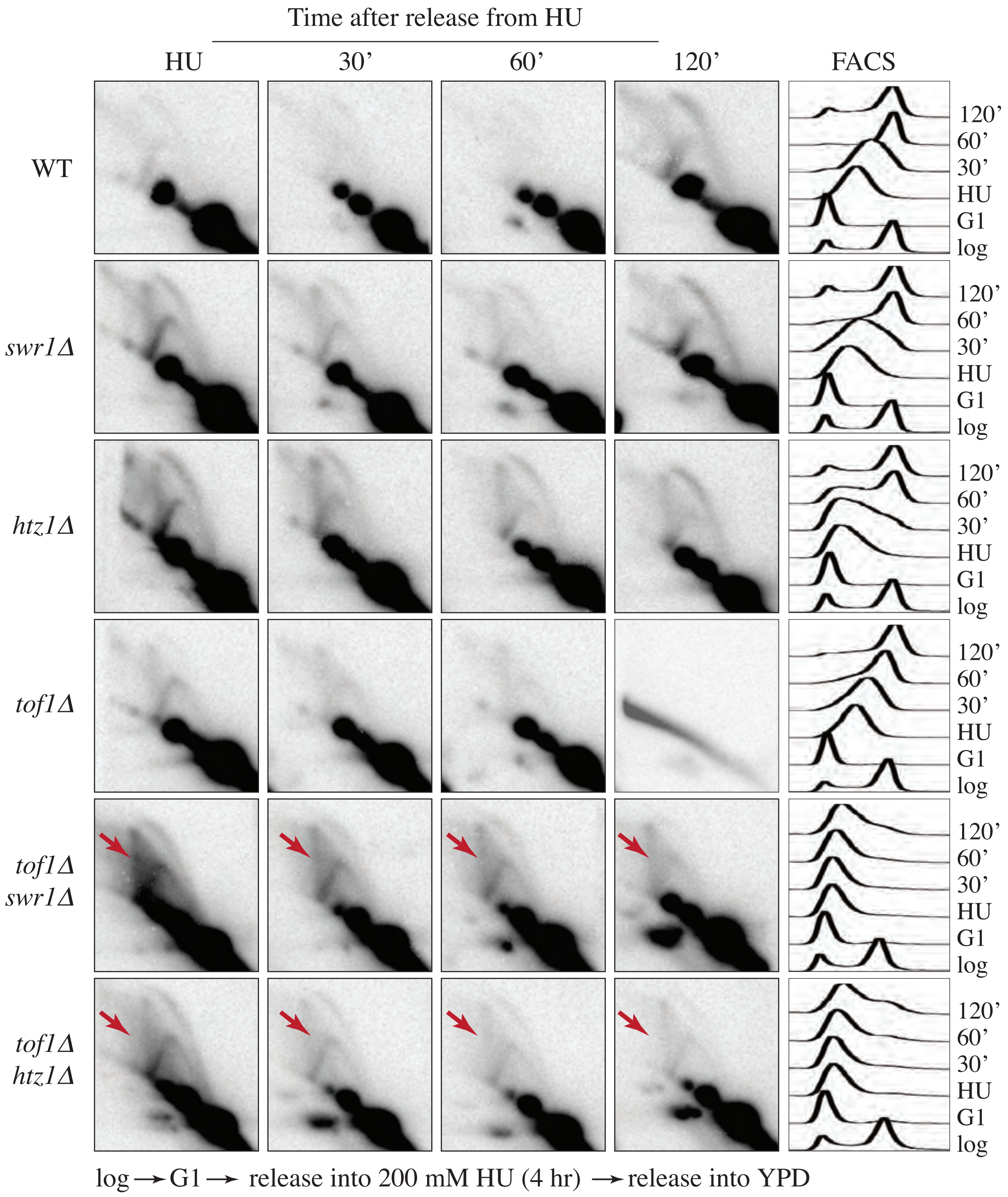

Supplementary Figure 18. Profile of replication intermediates after release from HU treatment in *tof1* strains proficient or deficient in the SWR-C/Htz1. 2D gels and FACS profiles of the indicated strains released into normal YPD medium after treatment with 200 mM HU for 4 hours. The profile of replication intermediates revealed by 2D gels corresponds to the ARS305 region as in Fig. 5A.

Supplementary Figure 19

A

```
Mrc1 1-843-EDDVLQQYRL KRRELMRKRR LEIGDDAKLV KNPREQKLGT ITSYNIHYTK
           LSGIRRAGEQ KLISEEDLNG EQKLISEEDL NGLDGEQKLI SEEDLNGEQK
           LISEEDLNGE QKLISEEDLN GLDGEQKLIS EEDLNGEQKL ISEEDLNGEQ
           KLISEEDLNG LDGEQKLISE EDLNGEQKLI SEEDLNGEQK LISGEDLNGL
           DGEQKLISEE DLNGEQKLIS EEDLNGEQKL INH.
Mrc1 1-843-RTLQVDGSPG .
```

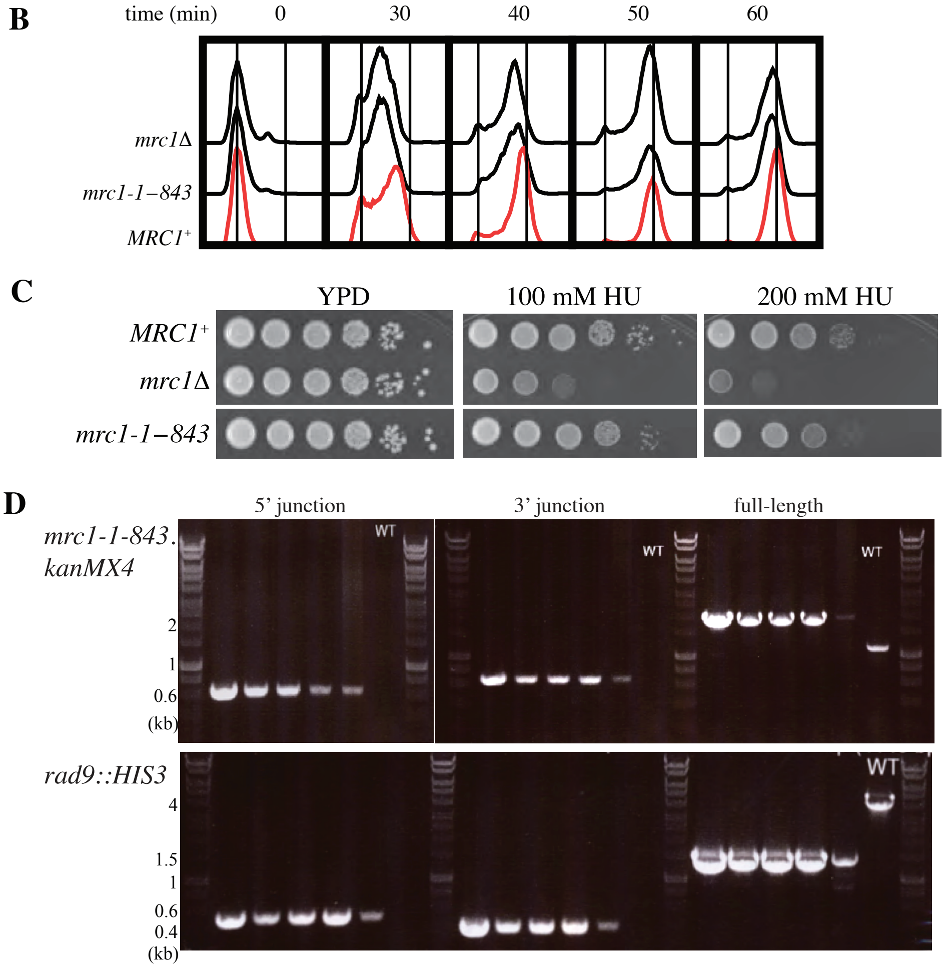

Supplementary Figure 19. The *mrc1-1-843* truncation is a replication-defective separation-of-function allele. A. Sequences of the previously published Mrc1-c14 mutant protein (upper)[1] and the Mrc1-1-843 construct generated in this study (lower). In each case, the amino acid sequence downstream of Mrc1 amino acid 843 is shown. **B.** Replication profiles of the wild-type (BY4741), *mrc1-1-843* (RDKY9285) and BY4741 *mrc1Δ* (obtained from the BY4741 *MAT***a** yeast deletion collection) strains after release from alpha-factor-mediated arrest. Log-phase cultures were arrested in G1, released into fresh YPD medium, and samples were taken at the indicated time-points for FACS analysis. **C.** The *mrc1-1-843* mutation does not cause sensitivity to HU. Ten-fold serial dilutions of wild-type (RDKY7635), *mrc1Δ* (RDKY8301), and *mrc1-1-843* (RDKY8967) were plated on YPD and plates containing 100 or 200 mM HU. Plates were imaged after 2 days of growth at 30°C. The white horizontal line indicates the position where images from the same plate were spliced together for clarity. **D.** The *mrc1-1-843* mutation does not cause synthetic lethality when combined with deletion of *RAD9*. Gel images showing the PCR reactions used to verify the *mrc1-1-843* truncation mutation (upper) and the deletion of *RAD9* (lower) in 5 independent isolates containing *mrc1-1-843.kanMX4* and *rad9Δ::HIS3* (RDKY9286-9290). For each isolate, 3 pairs of PCR reactions were performed: one each to verify the 5' (left) and 3' (middle) insertion junctions and a third reaction (right) to amplify across the *kanMX4* insertion site in the *mrc1-1-843.kanMX4* locus or the entire *rad9Δ::HIS3* deletion. A control reaction was performed for all primer pairs using wild-type genomic DNA as a template. The product sizes expected for the 5' junction, 3' junction, and full-length PCRs were 637 bp (0 bp in wild-type), 678 bp (0 bp in wild-type) and 1902 bp (1155 bp in wild-type), respectively, for *mrc1-1-843.kanMX4* and 500 bp (0 bp in wild-type), 396 bp (0 bp in wild-type), and 1467 bp (4143 bp in wild-type), respectively, for *rad9::HIS3*.

Supplementary Table 1. dGCR rates for strains with single and multiple mutations affecting *MRC1*, *TOF1*, *CSM3*, *SWR1*, and *HTZ1*[^].

	SWR1⁺			***swr1Δ***		
**Relevant Genotype**	**RDKY**	**Rate (x 10^{-8})[&]**	**Fold change[#]**	**RDKY**	**Rate (x 10^{-8})**	**Fold change**
wild-type	7635	8.1 [6.4-15]	1	7785	16 [11-34]	2.0
mrc1Δ	8301	26 [18-44]	3.2	8302	409 [152-964]	50.3
htz1Δ	8969	14 [9.9-22]	1.7	9138	14 [11-24]	1.7
mrc1Δ htz1Δ	8975	350 [269-456]	43.1	9140	246 [163-591]	30.3
tof1Δ	8963	22 [16-37]	2.7	8971	51 [36-116]	6.3
mrc1Δ tof1Δ	9141	155 [122-316]	19	9143	1080 [542-2180]	132.9
mrc1-aq	8305	18 [12-34]	2.2	8306	19 [12-43]	2.3
mrc1-aq tof1Δ	9145	39 [22-80]	4.8	9147	91 [49-154]	11
mrc1-1-843	8967	22 [16-30]	2.7	8973	219 [132-341]	27
mrc1-1-843 tof1Δ	9150	158 [123-230]	20	9152	1340 [560-1810]	164.5
csm3Δ	8965	34 [20-76]	4.2	8972	72 [50-95]	8.9
mrc1Δ csm3Δ	9154	131 [86-168]	16	9156	1730 [978-3360]	212.5
mrc1-aq csm3Δ	9158	30 [20-112]	3.7	9160	60 [29-153]	7.4
mrc1-1-843 csm3Δ	9163	147 [134-218]	18.0	9166	933 [819-4590]	115

[^] P values for significance calculated using the Mann-Whitney 2-tailed test are presented in Supplementary Data 1.

[&] The numbers in square brackets represent the 95% confidence interval for each rate.

[#] Fold change = fold change with respect to the wild-type dGCR rate.

Supplementary Table 2. dGCR rates for mutations affecting Htz1 sumoylation, acetylation, and retention in chromatin^.

**Relevant Genotype**	**WT**		***mrc1Δ***		***tof1Δ***		***csm3Δ***	
	**RDKY**	**Rate (x 10^{-8})&#**	**RDKY**	**Rate (x 10^{-8})**	**RDKY**	**Rate (x 10^{-8})**	**RDKY**	**Rate (x 10^{-8})**
HTZ1+	7635	8.1 [6.4-15] (1)	8301	26 [18-44] (3.2)	8963	22 [16-37] (2.7)	8965	34 [20-76] (4.2)
htz1Δ	8969	14 [9.9-22] (1.7)	8975	350 [269-456] (43)	9119	105 [48-201] (13)	9134	90 [59-202] (11)
htz1-K(126,133)R	9169	8.7 [5.6-12] (1.1)	9171	26 [18-50] (3.2)	9173	23 [22-91] (2.9)	9174	38 [24-94] (4.7)
htz1-K(4,9,11,15)R	9248	11 [6.6-21] (1.3)	9256	41 [32-54] (5)		n.d.		n.d.
htz1-K(4,9,11,15)Q	9251	6.4 [3.6-16] (0.8)	9258	32 [25-36] (4)		n.d.		n.d.
htz1-K(4,9,11,15,126,133)R	9260	18 [12-28] (2.2)	9263	56 [34-79] (6.9)		n.d.		n.d.
htz1—1-114	9265	10 [6.9-21] (1.3)	9269	240 [162-305] (30)		n.d.		n.d.
htz1—1-120	9267	10 [6.5-18] (1.2)	9271	79 [50-111] (10)		n.d.		n.d.

^ P values for significance calculated using the Mann-Whitney 2-tailed test are presented in Supplementary Data 1.

& The numbers in square brackets represent the 95% confidence interval for each rate.

The numbers within parentheses represent the fold change in rate with respect to the wild-type rate.

*The dGCR rate of a strain (RDKY9167) with wild-type *HTZ1* tagged with *HIS3* analogously to the *HIS3*-tagged *htz1* sumoylation, acetylation, and truncation mutants is 9.0 [6.6-18] x 10^{-8} (1.1).

Supplementary Table 3. Statistics for Whole-Genome Sequencing results.

Sample	Relevant Genotype	No. Read Pairs*	% Read 1 Mapped	% Read 2 Mapped	No. Uniquely Mapping Read Pairs	Median Intra-Read Pair Distance (bp)	Median Read Depth**
ASY987	uGCR wild-type	23,376,079 (24,496,086)	98.12%	93.63%	19,050,833	298	178
ASY988	uGCR wild-type	30,104,520 (34,582,188)	99.40%	94.86%	25,187,017	414	239
ASY989	uGCR wild-type	18,935,568 (20,303,221)	99.31%	93.97%	15,608,784	348	141
ASYG281	uGCR wild-type (from ASY989)	17,126,153 (18,420,892)	99.30%	93.15%	13,866,101	372	123
ASYG282	uGCR wild-type (from ASY989)	12,424,567 (12,927,135)	99.11%	90.73%	9,846,983	419	93
ASYG283	uGCR wild-type (from ASY989)	19,627,703 (21,105,027)	99.06%	90.93%	15,646,363	416	149
ASYG284	uGCR wild-type (from ASY989)	16,057,803 (16,862,585)	99.01%	92.69%	13,015,468	370	117
ASYG285	uGCR wild-type (from ASY989)	20,722,556 (23,455,425)	99.34%	92.35%	16,738,649	401	139
ASYG286	uGCR wild-type (from ASY989)	28,872,532 (32,459,697)	99.09%	92.80%	23,636,298	342	213
ASYG287	uGCR wild-type (from ASY989)	31,291,982 (37,403,924)	99.08%	93.63%	25,938,879	337	230
ASYG288	uGCR wild-type (from ASY987)	17,920,347 (20,922,770)	99.26%	93.91%	14,573,648	349	129
ASYG289	uGCR wild-type (from ASY987)	28,430,642 (32,134,880)	99.34%	93.78%	23,589,825	362	224
ASYG291	uGCR wild-type (from ASY987)	18,387,074 (19,236,609)	99.27%	93.98%	15,352,397	346	144
ASYG292	uGCR wild-type (from ASY987)	41,478,740 (52,132,280)	99.45%	94.57%	34,948,818	409	330
ASYG293	uGCR wild-type (from ASY987)	30,859,997 (35,252,546)	99.40%	94.99%	26,038,189	390	249
ASYG294	uGCR wild-type (from ASY987)	218,15,526 (23,794,909)	99.27%	94.33%	17,986,885	400	164
ASYG470	uGCR wild-type (from ASY988)	24,572,077 (26,199,707)	99.26%	94.90%	20,490,971	373	189
ASYG471	uGCR wild-type (from ASY988)	34,296,268 (40,891,308)	99.47%	94.27%	28,720,356	435	273
ASYG472	uGCR wild-type (from ASY988)	28,277,304 (31,820,162)	99.40%	94.89%	23,858,706	390	220
ASYG473	uGCR wild-type (from ASY988)	25,038,346 (27,784,280)	99.36%	95.32%	20,947,262	367	196
ASYG474	uGCR wild-type (from ASY988)	23,821,750 (25,245,187)	98.84%	95.20%	19,758,486	326	177
ASYG475	uGCR wild-type (from ASY988)	21,370,789 (22,545,474)	99.28%	96.09%	17,857,241	327	165
ASYG476	uGCR wild-type (from ASY988)	20,661,165 (22,175,618)	99.40%	95.93%	17,377,040	362	156
ASY1118	uGCR *swr1*	44,704,402 (52,521,068)	99.05%	93.93%	40,748,537	364	341
ASY1119	uGCR *swr1*	31,892,154 (36,536,444)	99.07%	93.22%	26,252,341	408	245
ASY1120	uGCR *swr1*	24,071,282 (26,346,057)	98.86%	94.27%	19,852,334	387	182
ASYG308	uGCR *swr1* (from ASY1118)	32,308,775 (37,186,153)	98.42%	91.70%	26,047,256	383	229
ASYG309	uGCR *swr1*	18,330,773	97.87%	89.83%	14,110,468	427	125

	(from ASY1118)	(19,949,184)					
ASYG310	uGCR *swr1*<br>(from ASY1118)	23,209,649<br>(25,397,978)	98.45%	91.70%	18,563,880	428	168
ASYG311	uGCR *swr1*<br>(from ASY1118)	25,269,217<br>(27,475,319)	97.89%	91.79%	20,308,338	370	189
ASYG312	uGCR *swr1*<br>(from ASY1118)	21,984,126<br>(24,362,202)	98.84%	92.89%	17,817,112	388	160
ASYG313	uGCR *swr1*<br>(from ASY1118)	22,685,112<br>(24,279,571)	99.13%	93.40%	18,736,464	393	176
ASYG314	uGCR *swr1*<br>(from ASY1119)	31,184,705<br>(32,776,331)	98.82%	94.91%	26,184,530	326	243
ASYG315	uGCR *swr1*<br>(from ASY1119)	24,784,865<br>(26,404,563)	99.03%	94.47%	20,687,585	347	189
ASYG316	uGCR *swr1*<br>(from ASY1119)	19,342,810<br>(21,791,165)	99.02%	93.50%	15,504,598	392	136
ASYG317	uGCR *swr1*<br>(from ASY1119)	28,261,504<br>(31,299,346)	98.91%	94.07%	23,345,609	377	215
ASYG318	uGCR *swr1*<br>(from ASY1119)	26,190,588<br>(28,900,759)	98.54%	92.86%	21,465,271	401	192
ASYG484	uGCR *swr1*<br>(from ASY1120)	26,940,220<br>(29,168,815)	98.49%	93.31%	22,050,712	401	208
ASYG485	uGCR *swr1*<br>(from ASY1120)	32,899,599<br>(36,420,672)	98.96%	93.81%	27,441,725	405	246
ASYG486	uGCR *swr1*<br>(from ASY1120)	30,633,914<br>(33,057,051)	98.88%	95.00%	25,664,687	329	233
ASYG487	uGCR *swr1*<br>(from ASY1120)	36,057,219<br>(38,915,348)	98.98%	95.24%	30,299,834	335	285
ASYG488	uGCR *swr1*<br>(from ASY1120)	24,971,894<br>(24,971,894)	97.64%	93.76%	19,686,956	343	184
ASYG489	uGCR *swr1*<br>(from ASY1120)	29,507,000<br>(32,170,499)	98.97%	95.09%	24,760,814	369	229
ASYG490	uGCR *swr1*<br>(from ASY1120)	29,336,235<br>(31,724,841)	99.04%	94.94%	24,530,467	376	226
ASY1112	uGCR *mrc1*	25,906,981<br>(28,124,516)	98.70%	94.08%	21,460,558	347	190
ASY1113	uGCR *mrc1*	31,156,000<br>(34,060,558)	98.90%	94.43%	26,181,552	332	235
ASY1114	uGCR *mrc1*	19,700,113<br>(21,139,074)	98.95%	94.44%	16,167,693	359	139
ASYG295	uGCR *mrc1*<br>(from ASY1112)	30,365,528<br>(33,363,371)	98.74%	93.83%	25,593,476	348	224
ASYG296	uGCR *mrc1*<br>(from ASY1112)	24,987,273<br>(26,222,585)	99.11%	94.54%	21,028,483	384	188
ASYG297	uGCR *mrc1*<br>(from ASY1112)	22,228,081<br>(24,585,461)	99.08%	93.99%	18,464,630	398	163
ASYG298	uGCR *mrc1*<br>(from ASY1112)	23,894,055<br>(26,023,966)	99.07%	94.15%	20,003,472	398	174
ASYG299	uGCR *mrc1*<br>(from ASY1112)	24,322,179<br>(26,014,929)	99.07%	94.39%	20,454,848	384	179
ASYG300	uGCR *mrc1*<br>(from ASY1112)	22,873,645<br>(23,934,690)	98.28%	93.44%	18,972,703	366	169
ASYG301	uGCR *mrc1*<br>(from ASY1112)	19,319,261<br>(21,673,803)	99.15%	96.16%	16,131,882	338	103
ASYG302	uGCR *mrc1*<br>(from ASY1113)	23,442,892<br>(25,613,614)	99.10%	94.24%	19,453,428	396	168
ASYG303	uGCR *mrc1*<br>(from ASY1113)	18,901,002<br>(19,727,778)	99.14%	94.57%	15,934,366	383	135
ASYG304	uGCR *mrc1*<br>(from ASY1113)	23,566,390<br>(33,614,488)	97.57%	87.69%	18,062,964	267	169
ASYG305	uGCR *mrc1*<br>(from ASY1113)	18,869,963<br>(25,043,473)	93.08%	80.11%	13,125,113	317	123
ASYG306	uGCR *mrc1*<br>(from ASY1113)	25,491,767<br>(36,914,552)	95.59%	85.17%	18,997,775	195	175

ASYG307	uGCR *mrc1* (from ASY1113)	22,977,152 (27,280,208)	97.15%	86.31%	17,756,996	370	155
ASYG477	uGCR *mrc1* (from ASY1114)	(31,488,670) 47,020,009	96.46%	87.88%	24,090,011	171	220
ASYG478	uGCR *mrc1* (from ASY1114)	17,021,917 (20,197,463)	96.68%	85.47%	12,850,489	380	117
ASYG479	uGCR *mrc1* (from ASY1114)	18,117,222 (19,556,363)	98.91%	89.87%	14,501,441	391	129
ASYG480	uGCR *mrc1* (from ASY1114)	17,825,332 (19,467,799)	98.74%	89.03%	14,200,885	400	125
ASYG481	uGCR *mrc1* (from ASY1114)	18,404,059 (19,086,258)	99.10%	89.95%	14,827,287	379	131
ASYG482	uGCR *mrc1* (from ASY1114)	15,911,684 (16,768,127)	98.98%	90.71%	12,806,725	381	109
ASYG483	uGCR *mrc1* (from ASY1114)	19,731,166 (21,910,983)	99.13%	89.02%	15,517,139	405	137
ASY1202	uGCR *mrc1 swr1*	20,187,141 (21,935,238)	99.30%	95.11%	16,831,125	393	143
ASY1203	uGCR *mrc1 swr1*	23,147,635 (24,599,624)	99.23%	95.09%	19,488,741	382	157
ASY1204	uGCR *mrc1 swr1*	26,231,330 (30,112,660)	99.29%	94.28%	22,054,922	420	186
ASYG319	uGCR *mrc1 swr1* (from ASY1202)	18,894,086 (20,078,195)	98.37%	93.05%	15,503,966	381	140
ASYG320	uGCR *mrc1 swr1* (from ASY1202)	23,474,939 (25,142,429)	99.03%	95.20%	19,838,503	363	172
ASYG321	uGCR *mrc1 swr1* (from ASY1202)	23,371,386 (24,322,843)	99.20%	95.09%	19,988,330	386	175
ASYG322	uGCR *mrc1 swr1* (from ASY1202)	21,143,713 (26,764,877)	99.21%	94.15%	17,570,103	420	147
ASYG323	uGCR *mrc1 swr1* (from ASY1202)	20,628,010 (25,303,427)	98.81%	93.14%	16,970,940	419	145
ASYG324	uGCR *mrc1 swr1* (from ASY1202)	21,169,437 (22,018,969)	99.05%	94.27%	17,748,137	429	158
ASYG325	uGCR *mrc1 swr1* (from ASY1202)	13,793,159 (14,256,293)	98.66%	92.78%	11,427,573	428	100
ASYG326	uGCR *mrc1 swr1* (from ASY1202)	18,306,956 (19,960,761)	98.85%	93.27%	15,270,946	420	138
ASYG327	uGCR *mrc1 swr1* (from ASY1203)	29,768,563 (33,728,163)	99.29%	95.26%	25,501,083	391	234
ASYG328	uGCR *mrc1 swr1* (from ASY1203)	22,037,490 (22,848,479)	99.04%	95.30%	18,779,033	359	168
ASYG329	uGCR *mrc1 swr1* (from ASY1203)	32,005,773 (33,955,557)	99.38%	95.66%	27,344,928	365	250
ASYG330	uGCR *mrc1 swr1* (from ASY1203)	32,929,505 (34,769,495)	99.34%	96.06%	28,389,751	345	253
ASYG331	uGCR *mrc1 swr1* (from ASY1203)	22,012,191 (23,223,768)	99.27%	95.14%	18,717,169	372	159
ASYG332	uGCR *mrc1 swr1* (from ASY1203)	26,415,686 (27,635,712)	99.29%	96.38%	22,811,727	338	202
ASYG333	uGCR *mrc1 swr1* (from ASY1203)	31,654,102 (33,309,334)	99.39%	95.53%	27,174,355	382	241
ASYG547	uGCR *mrc1 swr1* (from ASY1204)	27,730,453 (31,707,796)	99.28%	94.86%	23,498,772	398	201
ASYG548	uGCR *mrc1 swr1* (from ASY1204)	26,062,351 (28,254,234)	99.46%	95.86%	22,351,458	375	202
ASYG549	uGCR *mrc1 swr1* (from ASY1204)	19,475,864 (20,248,250)	99.33%	95.88%	16,513,147	364	145
ASYG550	uGCR *mrc1 swr1* (from ASY1204)	22,937,487 (23,906,687)	99.38%	95.88%	19,575,957	342	171
ASYG551	uGCR *mrc1 swr1* (from ASY1204)	28,082,532 (30,271,895)	99.21%	95.49%	24,040,217	362	221
ASYG554	uGCR *mrc1 swr1*	27,058,220	99.28%	95.79%	23,095,220	379	206

	(from ASY1204)	(30,421,857)					
ASYG555	uGCR *mrc1 swr1* (from ASY1204)	17,953,909 (18,583,766)	99.40%	95.78%	15,270,616	381	139
ASY566	sGCR *swr1*	8,132,022 (8,160,288)	99.65%	17.72%	1,219,615	325	34
ASYG176	sGCR *swr1* (from ASY566)	6,083,208 (6,417,764)	99.66%	18.67%	1,058,529	298	24
ASYG178	sGCR *swr1* (from ASY566)	7,840,029 8,305,509)	99.65%	18.66%	1,371,814	282	34
ASYG179	sGCR *swr1* (from ASY566)	6,401,948 (6,812,554)	99.61%	18.22%	1,086,278	317	28
ASYG180	sGCR *swr1* (from ASY566)	6,523,795 (6,886,909)	99.66%	18.11%	1,107,001	323	29
ASYG261	sGCR *swr1* (from ASY566)	5,534,129 (5,890,067)	99.70%	17.79%	914,939	337	24
ASYG262	sGCR *swr1* (from ASY566)	8,364,439 (8,901,945)	99.56%	18.89%	1,476,391	283	37
ASYG271	sGCR *swr1* (from ASY566)	7,743,516 (8,214,877)	99.67%	19.12%	1,387,035	279	35
ASYG272	sGCR *swr1* (from ASY566)	7,602,776 (8,077,050)	99.66%	17.98%	1,281,728	310	34
ASYG273	sGCR *swr1* (from ASY566)	6,180,114 (6,575,046)	99.52%	18.39%	1,055,044	306	26
ASYG274	sGCR *swr1* (from ASY566)	6,546,628 (6,919,868)	99.63%	18.61%	1,136,823	308	28
ASYG275	sGCR *swr1* (from ASY566)	5,124,147 (5,405,300)	99.58%	18.35%	873,930	320	22
ASY558	sGCR *mrc1*	14,333,092 (15,127,144)	99.37%	87.40%	11,165,893	324	100
ASYG158	sGCR *mrc1* (from ASY558)	16,106,100 (16,770,424)	99.33%	86.44%	12,077,980	346	109
ASYG159	sGCR *mrc1* (from ASY558)	12,357,394 (12,460,487)	99.31%	86.12%	9,317,786	343	78
ASYG160	sGCR *mrc1* (from ASY558)	9,399,184 (9,550,774)	99.09%	80.76%	6,516,200	361	58
ASYG161	sGCR *mrc1* (from ASY558)	15,649,712 (15,823,834)	99.74%	85.93%	12,285,214	305	99
ASYG162	sGCR *mrc1* (from ASY558)	15,274,592 (15,481,627)	99.76%	86.36%	11,898,470	305	98
ASYG163	sGCR *mrc1* (from ASY558)	15,602,016 (15,781,092)	99.75%	85.81%	12,068,700	307	105
ASYG164	sGCR *mrc1* (from ASY558)	18,501,076 (18,873,712)	99.75%	86.22%	14,464,523	304	126
ASYG266	sGCR *mrc1* (from ASY558)	17,399,408 (17,641,092)	99.75%	85.32%	13,578,075	296	117
ASYG267	sGCR *mrc1* (from ASY558)	17,320,966 (17,532,548)	99.70%	85.98%	13,442,946	288	121
ASYG268	sGCR *mrc1* (from ASY558)	18,536,042 (18,834,645)	99.71%	86.29%	14,395,392	298	128
ASYG269	sGCR *mrc1* (from ASY558)	16,614,439 (16,889,090)	99.69%	85.53%	12,814,861	302	114
ASY639	sGCR *mrc1-1-843*	16,598,566 (17,132,570)	99.66%	94.86%	13,656,572	318	117
ASYG187	sGCR *mrc1-1-843* (from ASY639)	17,363,334 (18,202,096)	99.57%	94.78%	14,357,597	323	122
ASYG188	sGCR *mrc1-1-843* (from ASY639)	16,970,060 (17,373,928)	99.48%	95.81%	14,291,186	311	124
ASYG189	sGCR *mrc1-1-843* (from ASY639)	20,364,927 (23,974,334)	99.57%	94.55%	16,667,128	340	137
ASYG190	sGCR *mrc1-1-843* (from ASY639)	15,444,735 (15,863,721)	99.49%	96.11%	12,879,723	285	103
ASYG191	sGCR *mrc1-1-843* (from ASY639)	14,922,403 (15,259,055)	99.63%	95.12%	12,451,784	324	103

ASYG263	sGCR *mrc1-1-843* (from ASY639)	15,739,640 (16,182,665)	99.26%	96.79%	13,292,124	227	106
ASYG264	sGCR *mrc1-1-843* (from ASY639)	15,336,826 (15,851,380)	99.44%	96.57%	12,908,815	203	102
ASYG276	sGCR *mrc1-1-843* (from ASY639)	14,882,008 (15,255,734)	99.54%	95.93%	12,356,206	268	103
ASYG277	sGCR *mrc1-1-843* (from ASY639)	13,516,114 (13,817,350)	99.39%	96.18%	11,373,699	224	95
ASYG278	sGCR *mrc1-1-843* (from ASY639)	15,305,197 (15,608,735)	99.55%	95.41%	12,860,362	323	104
ASYG279	sGCR *mrc1-1-843* (from ASY639)	13,327,796 (13,594,348)	99.49%	95.55%	11,171,737	316	90
ASY749	sGCR *mrc1 swr1*	16,019,761 (16,297,017)	98.84%	75.29%	11,417,214	334	103
ASYG230	sGCR *mrc1 swr1* (from ASY749)	22,341,396 (23,145,370)	96.71%	76.18%	15,631,724	340	142
ASYG231	sGCR *mrc1 swr1* (from ASY749)	16,810,296 (19,049,178)	96.90%	81.85%	11,765,989	307	67
ASYG232	sGCR *mrc1 swr1* (from ASY749)	18,379,715 (18,999,395)	98.60%	82.50%	13,763,311	309	124
ASYG233	sGCR *mrc1 swr1* (from ASY749)	24,416,574 (25,847,204)	98.72%	85.22%	18,692,689	278	176
ASYG234	sGCR *mrc1 swr1* (from ASY749)	29,110,586 (29,988,422)	99.19%	83.72%	22,436,223	294	202
ASYG235	sGCR *mrc1 swr1* (from ASY749)	26,066,871 (26,687,664)	99.11%	81.22%	19,321,575	317	181
ASYG236	sGCR *mrc1 swr1* (from ASY749)	18,536,267 (18,829,756)	98.68%	81.49%	13,972,877	316	129
ASYG237	sGCR *mrc1 swr1* (from ASY749)	25,489,689 (26,256,946)	98.76%	80.41%	18,953,624	323	169
ASYG238	sGCR *mrc1 swr1* (from ASY749)	25,154,023 (25,793,412)	98.97%	81.84%	19,026,698	313	166
ASYG239	sGCR *mrc1 swr1* (from ASY749)	24,159,794 (24,619,315)	98.88%	83.17%	18,733,120	283	168
ASYG240	sGCR *mrc1 swr1* (from ASY749)	25,459,051 (26,194,764)	99.09%	80.29%	18,900,653	315	169
ASY746	sGCR *mrc1-1-843 swr1*	18,906,039 (19,253,813)	98.94%	82.72%	14,242,860	320	128
ASYG200	sGCR *mrc1-1-843 swr1* (from ASY746)	36,698,543 (39,470,655)	99.13%	80.54%	26,469,373	356	248
ASYG201	sGCR *mrc1-1-843 swr1* (from ASY746)	20,837,549 (22,366,357)	99.06%	79.59%	14,582,871	368	125
ASYG202	sGCR *mrc1-1-843 swr1* (from ASY746)	31,777,246 (32,625,570)	99.13%	85.97%	24,865,119	308	238
ASYG203	sGCR *mrc1-1-843 swr1* (from ASY746)	22,370,717 (24,006,033)	99.05%	82.47%	16,309,000	341	151
ASYG204	sGCR *mrc1-1-843 swr1* (from ASY746)	37,884,998 (38,997,141)	99.20%	84.46%	29,065,296	323	269
ASYG205	sGCR *mrc1-1-843 swr1* (from ASY746)	33,478,896 (34,669,412)	99.11%	84.37%	25,261,235	327	237
ASYG206	sGCR *mrc1-1-843 swr1* (from ASY746)	21,088,276 (21,416,385)	99.14%	84.36%	16,177,114	331	145
ASYG208	sGCR *mrc1-1-843 swr1* (from ASY746)	24,020,049 (24,702,758)	99.10%	83.73%	18,102,267	322	166
ASYG209	sGCR *mrc1-1-843*	20,749,491	98.91%	86.04%	16,212,667	275	145

	swr1 (from ASY746)	(21,113,394)					
ASYG210	sGCR *mrc1-1-843* *swr1* (from ASY746)	26,864,647 (27,600,826)	99.09%	85.78%	20,886,454	301	177

*Numbers in parentheses indicate the number of read pairs prior to culling PCR duplicates. Reads were 50 bases long.
**Median read depth is the median number of times that each base in uniquely mapping regions of the nuclear genome was present within a read.

Supplementary Table 4. GCR structures for Can^R 5FOA^R isolates from the uGCR assay with sequenced genomes.

Sample (Relevant genotype)	GCR description	GCR Junction Evidence[1]
ASYG281 (wild-type)	Hairpin-mediated inverted duplication on chr V L (hairpin inside *NAT* at *can1::P_{LEU2}-NAT*, 36-base loop)	Copy number (Supplementary Fig. 3b)<br>Junction sequence (17 reads; Supplementary Fig. 12D)
	Homology-mediated translocation between chr V L *YELWdelta1/2* and chr V L *YELCdelta4*	Copy number (Supplementary Fig. 3b)<br>Discordant read pairs (6 read pairs)
	Homology-mediated translocation between chrV L *IMT4* and chr X R *IMT3*	Copy number (Supplementary Fig. 3b)<br>Discordant read pairs (205 read pairs)
ASYG282 (wild-type)	*de novo* telomere addition on chr V L at 34,800 bp (inside *NPR2*)	Copy number (Supplementary Fig. 3a)<br>Junction sequence (16 reads; Supplementary Fig. 3a)
ASYG283 (wild-type)	*de novo* telomere addition on chr V L at 34,872 bp (inside *NPR2*)	Copy number (Supplementary Fig. 3a)<br>Junction sequence (54 reads; Supplementary Fig. 3a)
ASYG284 (wild-type)	*de novo* telomere addition on chr V L at 39,748 bp	Copy number (Supplementary Fig. 3a)<br>Junction sequence (18 reads; Supplementary Fig. 3a)
ASYG285 (wild-type)	*de novo* telomere addition on chr V L at 26,384 bp (inside *YEL067C*)	Copy number (Supplementary Fig. 3a)<br>Junction sequence (56 reads; Supplementary Fig. 3a)
ASYG286 (wild-type)	*de novo* telomere addition on chr V L at 34,850 bp (inside *NPR2*)	Copy number (Supplementary Fig. 3a)<br>Junction sequence (43 reads; Supplementary Fig. 3a)
ASYG287 (wild-type)	Hairpin-mediated inverted duplication on chr V L (hairpin inside *NAT* at *can1::P_{LEU2}-NAT*, 135-base loop)	Copy number (Supplementary Fig. 3b)<br>Discordant read pairs (72 read pairs)<br>Junction sequence (52 reads; Supplementary Fig. 12D)
	Homology-mediated translocation between chr V L *YELCdelta4* and chr V R *YERCdelta14*	Copy number (Supplementary Fig. 3b)<br>Discordant read pairs (23 read pairs)
ASYG288 (wild-type)	Hairpin-mediated inverted duplication on chr V L (hairpin inside *NAT* at *can1::P_{LEU2}-NAT*, 22-base loop)	Copy number (Supplementary Fig. 3b)<br>Junction sequence (9 reads; Supplementary Fig. 12E)
	Homology-mediated translocation between chr V L *PAU2* and chr I R *PAU8*	Copy number (Supplementary Fig. 3b)<br>Discordant read pairs (31 read pairs)
ASYG289 (wild-type)	*de novo* telomere addition on chr V L at 34,842 bp (inside *NPR2*)	Copy number (Supplementary Fig. 3a)<br>Junction sequence (130 reads; Supplementary Fig. 3a)
ASYG291 (wild-type)	*de novo* telomere addition on chr V L at 34,845 bp (inside *NPR2*)	Copy number (Supplementary Fig. 3a)<br>Junction sequence (56 reads; Supplementary Fig. 3a)
ASYG292 (wild-type)	*de novo* telomere addition on chr V L at 34,849 bp (inside *NPR2*)	Copy number (Supplementary Fig. 3a)<br>Junction sequence (196 reads; Supplementary Fig. 3a)
ASYG293 (wild-type)	*de novo* telomere addition on chr V L at 34,835 bp (inside *NPR2*)	Copy number (Supplementary Fig. 3a)<br>Junction sequence (71 reads; Supplementary Fig. 3a)
ASYG294 (wild-type)	*de novo* telomere addition on chr V L inside *NAT* at *can1::P_{LEU2}-NAT*	Copy number (Supplementary Fig. 3a)<br>Junction sequence (16 reads; Supplementary Fig. 3a)
ASYG470 (wild-type)	*de novo* telomere addition on chr V L at 26,524 bp inside *YEL067C*	Copy number (Supplementary Fig. 3a)<br>Junction sequence (28 reads; Supplementary Fig. 3a)
ASYG471 (wild-type)	No GCR; point mutations inside *CAN1* (c.1000G>C, GGC->CGC, p.334G>R) and *URA3* (c.179C>A, ACA->AAA, p.60T>K)	Genome sequence
ASYG472 (wild-type)	*de novo* telomere addition on chr V L at 35,574 bp inside *NPR2*	Copy number (Supplementary Fig. 3a)<br>Junction sequence (47 reads;

		Supplementary Fig. 3a)
ASYG473 (wild-type)	*de novo* telomere addition on chr V L at 26,386 bp inside *YEL067C*	Copy number (Supplementary Fig. 3a) Junction sequence (84 reads; Supplementary Fig. 3a)
ASYG474 (wild-type)	*de novo* telomere addition on chr V L at 26,931 bp inside *HPA3*	Copy number (Supplementary Fig. 3a) Junction sequence (35 reads; Supplementary Fig. 3a)
ASYG475 (wild-type)	Interstitial deletion from 21,011 bp (inside *DSF1*) to 40,300 bp, 8 bp microhomology	Copy number (Supplementary Fig. 3b) Junction sequence (61 reads; Supplementary Fig. 3b)
ASYG476 (wild-type)	*de novo* telomere addition on chr V L at 27,780 bp inside *SIT1*	Copy number (Supplementary Fig. 3a) Junction sequence (54 reads; Supplementary Fig. 3a)
ASYG308 (*swr1*)	Hairpin-mediated inverted duplication on chr V L (hairpin inside *NAT* at *can1::*P_{LEU2}*-NAT*, 36-base loop)	Copy number (Supplementary Fig. 4b) Junction sequence (6 reads; Supplementary Fig. 12G)
	Homology-mediated translocation between chr V L *YELCdelta4* and chr II L *YBLWdelta2*	Copy number (Supplementary Fig. 4b) Discordant read pairs (66 read pairs)
ASYG309 (*swr1*)	Microhomology-containing translocation between chr V L (42,293 bp) and chr XIII L (141,052 bp, inside *SMA2*) with 2 bp microhomology	Copy number (Supplementary Fig. 4b) Discordant read pairs (353 read pairs) Junction sequence (96 reads)
ASYG310 (*swr1*)	Hairpin-mediated inverted duplication on chr V L (hairpin inside *NPR2*, 12 base loop)	Copy number (Supplementary Fig. 4b) Discordant read pairs (51 read pairs) Junction sequence (41 reads)
	Homology-mediated translocation between chr V L *YELWdelta1*/ *YELWdelta2* and chr V R "*YERWdelta27*", an unannotated delta at chrV:449,322-449,631	Copy number (Supplementary Fig. 4b) Discordant read pairs (52 read pairs)
ASYG311 (*swr1*)	Interstitial deletion on chr V L from 20,497 bp (inside *DSF1*) – 36,162 bp (inside *NPR2*) with 5 bp microhomology	Copy number (Supplementary Fig. 4a) Junction sequence (29 reads; Supplementary Fig. 4a)
ASYG312 (*swr1*)	Microhomology-containing translocation between chr V L (36,578 bp) and an unidentified full-length Ty	Copy number (Supplementary Fig. 4b) Junction sequence
	Homology-mediated translocation between a Ty element and chr III R *YCRWdelta8*, *9*, or *10*	Copy number (Supplementary Fig. 4b)
ASYG313 (*swr1*)	*de novo* telomere addition on chr V L at 29,943 bp inside *AVT2*	Copy number (Supplementary Fig. 4a) Junction sequence (59 reads; Supplementary Fig. 4a)
ASYG314 (*swr1*)	Interstitial deletion on chr V L from 15,497 bp to 32,566 bp inside *CAN1* at *yel068c::CAN1-URA3*	Copy number (Supplementary Fig. 4a) Discordant read pairs (663 read pairs) Junction sequence (169 reads; Supplementary Fig. 4a)
ASYG315 (*swr1*)	*de novo* telomere addition on chr V L at 34,845 bp inside *NPR2*	Copy number (Supplementary Fig. 4a) Junction sequence (46 reads; Supplementary Fig. 4a)
ASYG316 (*swr1*)	Hairpin-mediated inverted duplication on chr V L (hairpin inside *PRB1*, 25 base loop)	Copy number (Supplementary Fig. 4b) Discordant read pairs (78 read pairs) Junction sequence (116 reads; Supplementary Fig. 12G)
	de novo telomere addition at 83,912 bp inside *ANP1* on chr V L	Copy number (Supplementary Fig. 4b) Junction sequence (190 reads; Supplementary Fig. 4b)
ASYG317 (*swr1*)	Hairpin-mediated inverted duplication on chr V L (hairpin inside *NAT* at *can1::*P_{LEU2}*-NAT*, 254 base loop)	Copy number (Supplementary Fig. 4b) Junction sequence (9 reads; Supplementary Fig. 12H
	Homology-mediated translocation between chr V L *PAU2* and either chr IX-L *PAU14* or chr X-L *PAU1*; sequences from telomere to *PAU1/PAU14* are identical, hence the junction is not distinguishable	Copy number (Supplementary Fig. 4b) Junction sequence
ASYG318 (*swr1*)	Microhomology-containing translocation between chr V L (25,840 bp in *YEL068C* at *yel068c::CAN1-URA3*) and chr IV R (903,251 bp inside *RAD9*) with 12 bp of imperfect microhomology (11/12 bp)	Copy number (Supplementary Fig. 4b) Discordant read pairs (679 read pairs) Junction sequence (89 reads)
ASYG484	*de novo* telomere addition on chr V L at 34,421 bp inside *NPR2*	Copy number (Supplementary Fig. 4a)

(*swr1*)		Junction sequence (19 reads; Supplementary Fig. 4a)
ASYG485 (*swr1*)	Hairpin-mediated inverted duplication on chr V L (hairpin inside *NAT* at *can1::*P_{LEU2}*-NAT*, 130-base loop)	Copy number (Supplementary Fig. 4b) Discordant read pairs (96 read pairs) Junction sequence (95 reads; Supplementary Fig. 12J)
	Homology-mediated translocation between chr V L *tR(UCU)E* and chr XI L *tR(UCU)K*	Copy number (Supplementary Fig. 4b) Discordant read pairs (544 read pairs)
ASYG486 (*swr1*)	*de novo* telomere addition on chr V L inside *NAT* at *can1::*P_{LEU2}*-NAT*	Copy number (Supplementary Fig. 4a) Junction sequence (56 reads; Supplementary Fig. 4a)
ASYG487 (*swr1*)	*de novo* telomere addition on chr V L at 34,831 bp inside *NPR2*	Copy number (Supplementary Fig. 4a) Junction sequence (57 reads; Supplementary Fig. 4a)
ASYG488 (*swr1*)	*de novo* telomere addition on chr V L at 34,831 bp inside *NPR2*	Copy number (Supplementary Fig. 4a) Junction sequence (61 reads; Supplementary Fig. 4a)
ASYG489 (*swr1*)	*de novo* telomere addition on chr V L at 91,326 bp in P_{LEU2} in *can1::*P_{LEU2}*-NAT*	Copy number (Supplementary Fig. 4a) Junction sequence (35 reads; Supplementary Fig. 4a)
ASYG490 (*swr1*)	Hairpin-mediated inverted duplication on chr V L (hairpin inside *NAT* at *can1::*P_{LEU2}*-NAT*, 298-base loop)	Copy number (Supplementary Fig. 4b) Junction sequence (7 reads; Supplementary Fig. 12J)
	Homology-mediated translocation between chr V L *YELWdelta6* and chr XV L *YOLCdelta1*	Copy number (Supplementary Fig. 4b) Discordant read pairs (347 read pairs)
ASYG295 (*mrc1*)	Hairpin-mediated inverted duplication on chr V L (hairpin inside *NPR2*, 269-base loop) followed by whole-chromosome duplication	Copy number (Supplementary Fig. 5a) Discordant read pairs (469 read pairs) Junction sequence (201 reads; Supplementary Fig. 12E)
	Deletion of one copy of *CEN5*	Copy number (Supplementary Fig. 5a) Discordant read pairs (353 read pairs) Junction sequence (129 reads)
	Interstitial deletion (deleting one copy) on chr V R between *YERCTy1-1* and *YERCTy1-2*	Copy number (Supplementary Fig. 5a)
ASYG296 (*mrc1*)	Hairpin-mediated inverted duplication on chr V L (hairpin inside *PRB1*, 26-base loop)	Copy number (Supplementary Fig. 5a) Discordant read pairs (142 read pairs) Junction sequence (132 reads; Supplementary Fig. 12E)
	Homology-mediated translocation between chr V L *YELCdelta4* and chr II L *YBLWdelta1*	Copy number (Supplementary Fig. 5a) Discordant read pairs (59 read pairs)
ASYG297 (*mrc1*)	Microhomology-containing translocation between chr V L (30,089 bp, inside *AVT2*) and chr IV R (either *YDRWTy2-2* or *YDRCTy1-3*)); and chr V R (*YERCTy1-1*) with 13 bp identity at junctions; presence of other intermediate Ty elements in the junction is not known	Copy number (Supplementary Fig. 5c) Junction sequence (23 reads; Supplementary Fig. 5c)
	Homology-mediated translocation between chr IV R (either *YDRWTy2-2* or *YDRCTy1-3*) and chr V R *YERCTy1-1*; presence of other intermediate Ty elements in the junction is not known	Copy number (Supplementary Fig. 5c)
ASYG298 (*mrc1*)	First altered chromosome (contains chrV R and centromeric portion of chrV L): Microhomology-containing translocation between chr V L (26,042 bp) and chr IV R (*YDRWTy1-5*)	Copy number (Supplementary Fig. 5c) Junction sequence (16 reads; Supplementary Fig. 5c)
	Second altered chromosome (contains telomeric portion of chr VL): Homology-mediated translocation between telomeric end of chr V L and chr XIV R (using *DSF1-HXT13* region on chr V L and *YNR073C-HXT17* region on chr XIV R), with deletion of terminal region of chr XIV	Copy number (Supplementary Fig. 5c)
ASYG299 (*mrc1*)	First altered chromosome (contains chrV R and centromeric portion of chrV L): Microhomology-containing translocation between chr V L (26,042 bp) and chr IV R (*YDRWTy1-5*)	Copy number (Supplementary Fig. 5c) Junction sequence (5 reads; Supplementary Fig. 5c)
	Second altered chromosome (contains telomeric portion of chr VL): Homology-mediated translocation between telomeric end of chr V L and chr XIV R (using *DSF1-HXT13* region on chr V	Copy number (Supplementary Fig. 5c)

	L and *YNR073C-HXT17* region on chr XIV R), with deletion of terminal region of chr XIV	
ASYG300 (*mrc1*)	Hairpin-mediated inverted duplication (hairpin within *NAT* at *can1::*P_{LEU2}*-NAT*, 70-base loop)	Copy number (Supplementary Fig. 5a)<br>Junction sequence (44 reads; Supplementary Fig. 12F)
	Homology-mediated translocation between chr V L *PAU2* and subtelomeric *PAU* gene, most likely chrII L *PAU9*	Copy number (Supplementary Fig. 5a)
ASYG301 (*mrc1*)	Hairpin-mediated inverted duplication on chr V L (41-base loop)	Copy number (Supplementary Fig. 5a)<br>Junction sequence (4 reads; Supplementary Fig. 12F)
	Homology-mediated translocation between chr V L *YELCdelta4* and chr IV R *YDRCTy1-2*	Copy number (Supplementary Fig. 5a)
ASYG302 (*mrc1*)	Hairpin-mediated inverted duplication on chr V L (hairpin inside *SIT1*, 31-base loop)	Copy number (Supplementary Fig. 5a)<br>Discordant read pairs (104 read pairs)<br>Junction sequence (94 reads; Supplementary Fig. 12F)
	Homology-mediated translocation between chr V L *YELWdelta1/2* and chr V R *"YERWdelta27"*, an unannotated delta at chrV:449,322-449,631	Copy number (Supplementary Fig. 5a)<br>Discordant read pairs (46 read pairs)
ASYG303 (*mrc1*)	Microhomology-containing translocation between chr V L (41,897 bp, inside *PRB1*) and chr IV R (1,257,951 bp, inside *YDR319C*)	Copy number (Supplementary Fig. 5c)<br>Discordant read pairs (559 read pairs)<br>Junction sequence (79 reads; Supplementary Fig. 5c)
ASYG306 (*mrc1*)	*de novo* telomere addition inside *NAT* (369 bp) at *can1::*P_{LEU2}*-NAT*	Copy number (Supplementary Fig. 5c)<br>Junction sequence (24 read pairs)
ASYG307 (*mrc1*)	Hairpin-mediated inverted duplication on chr V L (hairpin inside *HHY1*, 13-base loop)	Copy number (Supplementary Fig. 5a)<br>Discordant read pairs (57 read pairs)<br>Junction sequence (65 reads; Supplementary Fig. 12G)
	Homology-mediated translocation between chr V L *YELCdelta4* and chr IV L *YDRCTy1-3*	Copy number (Supplementary Fig. 5a)
ASYG477 (*mrc1*)	Hairpin-mediated inverted duplication on chr V L (hairpin inside *NPR2*, 12-base loop)	Copy number (Supplementary Fig. 5b)<br>Discordant read pairs (51 read pairs)<br>Junction sequence (80 reads; Supplementary Fig. 12H)
	Homology-mediated translocation between chr V L *YELWdelta1* chr V R "*YERWdelta27*", an unannotated delta at chrV:449,322-449,631	Copy number (Supplementary Fig. 5b)<br>Discordant read pairs (41 read pairs)
ASYG478 (*mrc1*)	Hairpin-mediated inverted duplication on chr V L (hairpin inside *PRB1*, 9-base loop)	Copy number (Supplementary Fig. 5b)<br>Discordant read pairs (39 read pairs)<br>Junction sequence (81 reads; Supplementary Fig. 12I)
	Homology-mediated translocation between chr V L *PAU2* and chr XII R *PAU4*	Copy number (Supplementary Fig. 5b)<br>Discordant read pairs (64 read pairs)
ASYG479 (*mrc1*)	Hairpin-mediated inverted duplication on chr V L (hairpin from *PRB1-PCM1*: 1,823-base loop)	Copy number (Supplementary Fig. 5b)<br>Discordant read pairs (579 read pairs)<br>Junction sequence (78 reads; Supplementary Fig. 12I)
	Homology-mediated translocation between chr V L *YELCdelta4* and chr XII R *YLRCTy2-2*	Copy number (Supplementary Fig. 5b)
ASYG480 (*mrc1*)	Hairpin-mediated inverted duplication on chr V L (hairpin inside *HPA3*, 111-base loop)	Copy number (Supplementary Fig. 5b)<br>Discordant read pairs (143 read pairs)<br>Junction sequence (58 reads)
	Homology-mediated translocation between chr V L *YELCdelta4* and chr IV R *YDRCTy1-2* or *YDRWTy2-3*	Copy number (Supplementary Fig. 5b)
	Homology-mediated translocation between chr IV R *YDRWTy2-3* or *YDRCTy1-2* and chr V R *YERWdelta22*	Copy number (Supplementary Fig. 5b)
ASYG481 (*mrc1*)	Hairpin-mediated inverted duplication on chr V L (hairpin inside *NAT* at *can1::*P_{LEU2}*-NAT*, 163-base loop)	Copy number (Supplementary Fig. 5b)
	Homology-mediated translocation between chr V L *YELCdelta4* and chr IV R *YDRCTy1-3*	Copy number (Supplementary Fig. 5b)
ASYG482 (*mrc1*)	Hairpin-mediated inverted duplication on chr V L (hairpin inside *NAT* at *can1::*P_{LEU2}*-NAT*, 163-base loop)	Copy number (Supplementary Fig. 5b)<br>Discordant read pairs (11 read pairs)

		Junction sequence (11 reads;<br>Supplementary Fig. 12I)
	Homology-mediated translocation between chr V L *YELCdelta4* and chr IV R *YDRCTy1-3*	Copy number (Supplementary Fig. 5b)
ASYG483<br>(*mrc1*)	Hairpin-mediated inverted duplication on chr V L (hairpin inside *NAT* at *can1::P_{LEU2}-NAT*, 61-base loop)	Copy number (Supplementary Fig. 5b)<br>Junction sequence (23 reads;<br>Supplementary Fig. 12J)
	Homology-mediated translocation between chr V L *YELCdelta4* and chr IV R *YDRCTy1-2*	Copy number
ASYG319<br>(*mrc1 swr1*)	Interstitial deletion on chr V L inside *yel072w::CAN1-URA3* from 116,282 bp (inside *URA3*) to 33,176 bp (inside *CAN1*) with 12 bp microhomology	Copy number (Supplementary Fig. 6a)<br>Discordant read pairs (323 read pairs)<br>Junction sequence (120 reads;<br>Supplementary Fig. 6a)
ASYG320<br>(*mrc1 swr1*)	Interstitial deletion (23,321-35,586 bp) 6 bp microhomology	Copy number (Supplementary Fig. 6a)<br>Discordant read pairs (216 read pairs)<br>Junction sequence (50 reads;<br>Supplementary Fig. 6a)
	Duplication on chr IV R (885,000 to 992,000 bp flanked by a pair of inverted Ty elements at each end)	Copy number (data not shown)
ASYG321<br>(*mrc1 swr1*)	Microhomology-containing translocation between chrV L (34997, inside *NPR2*) and chr XVI L (240,584 bp, inside *MLH3*)- 10 bp identity	Copy number (Supplementary Fig. 6b)<br>Discordant read pairs (418 read pairs)<br>Junction sequence (220 reads;<br>Supplementary Fig. 6b)
	Microhomology-containing translocation inside *MLH3* between 240,627 and 240,577; 7 bp microhomology	Copy number (Supplementary Fig. 6b)<br>Discordant read pairs (418 read pairs)<br>Junction sequence (220 reads;<br>Supplementary Fig. 6b)
ASYG322<br>(*mrc1 swr1*)	Microhomology-containing translocation between chr V L (27,282 bp) and chr IV L (5,121 bp) or chr X R (740,680 bp), 13 bp imperfect homology (12/13 bp microhomology)	Copy number (Supplementary Fig. 6b)<br>Junction sequence (49 reads;<br>Supplementary Fig. 6b)
ASYG323<br>(*mrc1 swr1*)	Microhomology-containing translocation between *NAT* at *can1*::*P_{LEU2}-NAT* (nt 219) and chr XVI R (918,337 bp, inside *RPC82*); 19 bp of imperfect homology (17/19 microhomology)	Copy number (Supplementary Fig. 6b)<br>Discordant read pairs (192 read pairs)<br>Junction sequence (20 reads;<br>Supplementary Fig. 6b)
ASYG324<br>(*mrc1 swr1*)	Microhomology-containing translocation between chr V L (inside *SIT1*, 29,425 bp) and chr XVI R (inside *ATG13*, 908,824 bp) with 12 bp of microhomology	Copy number (Supplementary Fig. 6b)<br>Discordant read pairs (617 read pairs)<br>Junction sequence (109 reads;<br>Supplementary Fig. 6b)
ASYG325<br>(*mrc1 swr1*)	Hairpin-mediated inverted duplication on chr V L (hairpin inside *CIN8*, 21-base loop)	Copy number (Supplementary Fig. 6c)<br>Discordant read pairs (42 read pairs)<br>Junction sequence (52 reads;<br>Supplementary Fig. 12H)
	Homology-mediated translocation between chr V L *YELCdelta4* and chr XII R *YLRWTy1-3*	Copy number (Supplementary Fig. 6c)<br>Discordant read pairs (43 read pairs)
	Homology-mediated translocation between chr XII R *YLRWTy1-2* and chr III L *YCLWTy2-1*	Copy number (Supplementary Fig. 6c)
ASYG326<br>(*mrc1 swr1*)	Microhomology-containing translocation between chr V L (inside *CIN8*, 36,729 bp) and chr II L (inside *YEL1*, 108,035 bp) with 6 bp microhomology	Copy number (Supplementary Fig. 6b)<br>Discordant read pairs (427 read pairs)<br>Junction sequence (80 reads;<br>Supplementary Fig. 6b)
ASYG327<br>(*mrc1 swr1*)	Interstitial deletion inside *URA3* and across *CAN1* (in *yel068c::CAN1-URA3* (116,693-25,841 bp) with 6 bp microhomology	Copy number (Supplementary Fig. 6a)<br>Discordant read pairs (815 read pairs)<br>Junction sequence (112 reads;<br>Supplementary Fig. 6a)
ASYG328<br>(*mrc1 swr1*)	Hairpin-mediated inverted duplication on chr V L (hairpin inside *NPR2*, 12-base loop)	Copy number (Supplementary Fig. 6c)<br>Discordant read pairs (33 read pairs)<br>Junction sequence ()
	Homology-mediated translocation between chr V L *YELWdelta1/2* and chr V R "*YERWdelta27*", an unannotated delta at chrV:449,322-449,631	Copy number (Supplementary Fig. 6c)<br>Discordant read pairs (30 read pairs)
ASYG329<br>(*mrc1 swr1*)	Microhomology-containing translocation between chr V L (37,543 bp, inside *CIN8*) and chr X L (27,189 bp, inside *HXT8*) with 10 bp microhomology	Copy number (Supplementary Fig. 6a)<br>Discordant read pairs (604 read pairs)<br>Junction sequence (117 reads;

		Supplementary Fig. 6a)
ASYG330<br>(*mrc1 swr1*)	Interstitial deletion from 21,337 bp (inside *DSF1*) to 40,003 bp with 16 bp imperfect microhomology (15/16 bp)	Copy number (Supplementary Fig. 6a)<br>Discordant read pairs (107 read pairs)<br>Junction sequence (42 reads;<br>Supplementary Fig. 6a)
ASYG332<br>(*mrc1 swr1*)	Microhomology-containing translocation between chr V L (38,625 bp, inside *CIN8*) and chr I L (85,486 bp, *GIP4*) with 7 bp microhomology	Copy number (Supplementary Fig. 6b)<br>Discordant read pairs (545 read pairs)<br>Junction sequence (103 reads;<br>Supplementary Fig. 6b)
ASYG333<br>(*mrc1 swr1*)	Interstitial deletion within *yel072w::CAN1-URA3*; 116,282 bp to 32,720 bp; 14 bp microhomology	Copy number (Supplementary Fig. 6a)<br>Discordant read pairs (798 read pairs)<br>Junction sequence (171 reads;<br>Supplementary Fig. 6a)
	Duplicated region within chrIV bounded by *YDRWTy1-4* and *YDRWTy1-5*, not obviously related to the GCR	Copy number (Data not shown)
ASYG547<br>(*mrc1 swr1*)	Interstitial deletion from 25,408 bp to 33,484 bp inside *yel072w::CAN1-URA3*, deletes *CAN1* promoter	Copy number (Supplementary Fig. 6a)<br>Discordant read pairs (601 read pairs)<br>Junction sequence (102 reads;<br>Supplementary Fig. 6a)
ASYG548<br>(*mrc1 swr1*)	Interstitial deletion from 25,412 bp to 29,418 bp (inside *SIT1*) across *yel072w::CAN1-URA3* with 3 bp microhomology	Copy number (Supplementary Fig. 6a)<br>Discordant read pairs (510 read pairs)<br>Junction sequence (74 reads;<br>Supplementary Fig. 6a)
	Disomy of chr I, also observed in parental strain ASY1204	Copy number histogram plot<br>(Supplementary Fig. 13)
ASYG549<br>(*mrc1 swr1*)	Interstitial deletion from 116,699 bp to 33,406 bp inside *yel068c::CAN1-URA3* with 4 bp microhomology	Copy number (Supplementary Fig. 6a)<br>Discordant read pairs (439 read pairs)<br>Junction sequence (71 reads;<br>Supplementary Fig. 6a)
	Disomy of chr I, also observed in parental strain ASY1204	Copy number histogram plot<br>(Supplementary Fig. 13)
ASYG550<br>(*mrc1 swr1*)	*de novo* telomere addition on chr V L at 34,405 bp	Copy number (Supplementary Fig. 6a)<br>Junction sequence (21 reads;<br>Supplementary Fig. 6a)
	Disomy of chr I, also observed in parental strain ASY1204	Copy number histogram plot<br>(Supplementary Fig. 13)
	Disomy of chr III	Copy number histogram plot<br>(Supplementary Fig. 13)
ASYG551<br>(*mrc1 swr1*)	Microhomology-containing translocation between chr V L (37,903 bp, inside *CIN8*) and chr IV L (27,031 bp, inside *ADY3*) with 4 bp microhomology	Copy number (Supplementary Fig. 6b)<br>Discordant read pairs (510 read pairs)<br>Junction sequence (103 reads;<br>Supplementary Fig. 6b)
	Disomy of chr I, also observed in parental strain ASY1204	Copy number histogram plot<br>(Supplementary Fig. 13)
ASYG554<br>(*mrc1 swr1*)	Microhomology-containing translocation between chr V L (32,138 bp, inside *CAN1* at *yel072w::CAN1-URA3*) and chr IX L (54,571 bp, inside *IMP2'*) with 14 bp imperfect microhomology	Copy number (Supplementary Fig. 6b)<br>Discordant read pairs (622 read pairs)<br>Junction sequence (177 reads;<br>Supplementary Fig. 6b)
	Non-reciprocal translocation between a telomeric fragment of chr V L and chr XIV R using the *DSF1-HXT13* and *YNR073C-HXT17* homology, with deletion of the telomeric fragment of chr XIV R	Copy number (Supplementary Fig. 6b)
	Disomy of chr I, also observed in parental strain ASY1204	Copy number histogram plot<br>(Supplementary Fig. 13)
ASYG555<br>(*mrc1 swr1*)	Interstitial deletion on chr V L from 23,168 bp to 41,480 bp; 7 bp microhomology	Copy number (Supplementary Fig. 6a)<br>Junction sequence (38 reads;<br>Supplementary Fig. 6a)
	Disomy of chr I, also observed in parental strain ASY1204	Copy number histogram plot<br>(Supplementary Fig. 13)

[1]Evidence supporting GCR-related junctions can be derived from whole genome sequencing data in several ways. Frequently, multiple lines of evidence support many junctions. Copy number changes, from normalized read depth plots of uniquely mapping regions of the genome, can suggest possible

rearrangements, particularly those involving HR between repetitive elements. Discordant read pairs are read pairs where two uniquely mapping reads map to regions consistent with the GCR, but not with reference chromosome; note that the number of discordant read pairs is influenced by whether the rearrangement is within or adjacent to non-uniquely mapping regions of the genome. Junction sequences can be determined in two ways, depending on the junction. For junctions that generate a novel sequence, such as a deletion or hairpin-mediated inversion, the sequence can be derived from read pairs adjacent to junction-defining discordant read pairs in which one read does not map to the reference and potentially sequences the novel junction. In these cases, the numbers of reads that align to define the novel junction sequence are reported. For junctions mediated by homologies, such as tRNA genes or Ty-related sequences, sequences can be determined by aligning reads that are linked to uniquely mapping regions adjacent to the homologies involved in the junction.

Supplementary Table 5. GCR structures for Can^R 5FOA^R isolates from the sGCR assay with sequenced genomes.

Sample (Relevant genotype)	GCR description	GCR Junction Evidence[1]
ASYG176 (*swr1*)	Hairpin-mediated inverted duplication on chr V L (hairpin between *NAT* at *can1::P_{LEU2}-NAT* and *PRB1* at 40,999 bp (7,437 base loop)	Copy number (Supplementary Fig. 7a)<br>Discordant read pairs (14 read pairs)<br>Junction sequence (4 reads; Supplementary Fig. 12B)
	Homology-mediated translocation between chr V L *YELCdelta4* and chr XII R *YLRCTy2-2*	Copy number (Supplementary Fig. 7a)
ASYG178 (*swr1*)	Homology-mediated inverted duplication on chr V L between *YCLWdelta5* at *can1::P_{LEU2}-NAT* and *YELWdelta1/2*	Copy number (Supplementary Fig. 7a)<br>Discordant read pairs (22 read pairs)
	de novo telomere addition on chr V L at 140,721 bp inside *YEL008W*	Copy number (Supplementary Fig. 7a)<br>Junction sequence (2 reads; Supplementary Fig. 7a)
ASYG179 (*swr1*)	*de novo* telomere addition on chr V L at 34,905 bp inside *NPR2*	Copy number (Supplementary Fig. 7a)<br>Junction sequence (7 reads; Supplementary Fig. 7a)
ASYG180 (*swr1*)	*de novo* telomere addition on chr V L at 38,542 bp inside *CIN8*	Copy number (Supplementary Fig. 7a)<br>Junction sequence (3 reads; Supplementary Fig. 7a)
ASYG261 (*swr1*)	Homology-mediated translocation between chr V L *YCLWdelta5* at *can1::P_{LEU2}-NAT* and an unidentified Ty-containing target	Copy number (Supplementary Fig. 7b)
	Homology-mediated translocation between the unidentified Ty-containing target and chr VIII R *YHRCTy1-1*	Copy number (Supplementary Fig. 7b)
ASYG262 (*swr1*)	Homology-mediated inverted duplication on chr V L between *YCLWdelta5* at *can1::P_{LEU2}-NAT* and *YELWdelta1/2*	Copy number (Supplementary Fig. 7a)<br>Discordant read pairs (27 read pairs)
	Homology-mediated translocation between chr V L *YELCdelta4* and chr V R *YERCTy1-1*	Copy number (Supplementary Fig. 7a)
ASYG271 (*swr1*)	Homology-mediated translocation between chr V L *YCLWdelta5* at *can1::P_{LEU2}-NAT* and chr XII L *YLLCdelta1*	Copy number (Supplementary Fig. 7b)<br>Discordant read pairs (12 read pairs)
ASYG272 (*swr1*)	*de novo* telomere addition on chr V L at 34,842 bp inside *NPR2*	Copy number (Supplementary Fig. 7a)<br>Junction sequence (6 reads; Supplementary Fig. 7a)
ASYG273 (*swr1*)	Homology-mediated inverted duplication on chr V L between *YCLWdelta5* at *can1::P_{LEU2}-NAT* and *YELWdelta1/2*	Copy number (Supplementary Fig. 7a)<br>Discordant read pairs (10 read pairs)
	Homology-mediated translocation between chr V L *YELCdelta4* and chr V R *YERCdelta16*	Copy number (Supplementary Fig. 7a)
ASYG274 (*swr1*)	Homology-mediated translocation between chr V L *YCLWdelta5* at *can1::P_{LEU2}-NAT* and chr V R "*YERWdelta27*", an unannotated delta at chrV:449,322-449,631	Copy number (Supplementary Fig. 7b)<br>Discordant read pairs (17 read pairs)
ASYG275 (*swr1*)	Homology-mediated inverted duplication on chr V L (mediated by pairing between *YCLWdelta5* at *can1::P_{LEU2}-NAT* and *YELWdelta1/2*	Copy number (Supplementary Fig. 7a)<br>Discordant read pairs (11 read pairs)
	Homology-mediated translocation between chr V L *PAU2* and another *PAU* gene, most likely chrII R *PAU24*	Copy number (Supplementary Fig. 7a)<br>Discordant read pairs (2 read pairs)
ASYG158 (*mrc1*)	Homology-mediated translocation between chr V L *YCLWdelta5* at *can1::P_{LEU2}-NAT* and chr V R "*YERWdelta27*", an unannotated delta at chrV:449,322-449,631	Copy number (Supplementary Fig. 8a)<br>Discordant read pairs (201 read pairs)
ASYG159 (*mrc1*)	*de novo* telomere addition on chr V L (at 34,849 bp inside *NPR2*)	Copy number (Supplementary Fig. 8b)<br>Junction sequence (25 reads;

		Supplementary Fig. 8b)
	Disomy of chr II and chr IX	Copy number histogram plot
ASYG160 (*mrc1*)	Homology-mediated translocation between chr V L *YCLWdelta5* at *can1::P_{LEU2}-NAT* and chr X R *YJRWdelta17*	Copy number (Supplementary Fig. 8a) Discordant read pairs (94 read pairs)
ASYG161 (*mrc1*)	Homology-mediated translocation between chrV L *YCLWdelta5* at *can1::P_{LEU2}-NAT* and chrV R *"YERWdelta27"*, an unannotated delta at chrV:449,322-449,631	Copy number (Supplementary Fig. 8a) Discordant read pairs (167 read pairs)
	Homology-mediated translocation between chr V R "*YERWdelta27*" and chrIV R *YDRWTy2-3*	Copy number (Supplementary Fig. 8a)
	Non-integral aneuploidy of chr II and chr IX	Copy number histogram plot
ASYG162 (*mrc1*)	Homology-mediated translocation between chr V L *YCLWdelta5* at *can1::P_{LEU2}-NAT* and chr IV R *YDRWTy2-2*	Copy number (Supplementary Fig. 8a) Discordant read pairs (103 read pairs)
	Non-integral aneuploidy of chr IX	Copy number histogram plot
ASYG163 (*mrc1*)	Homology-mediated translocation between chrV L *YCLWdelta5* at *can1::P_{LEU2}-NAT* and chrV R *"YERWdelta27"*, an unannotated delta at chrV:449,322-449,631	Copy number (Fig. 8a) Discordant read pairs (143 read pairs)
	Homology-mediated translocation between chr V R "*YERWdelta27*" and an unidentified Ty-containing target	-
	Homology-mediated translocation between an unidentified Ty-containing target and chrV R *YERCTy1-2*	Copy number (Fig. 8a)
	Non-integral aneuploidy of chr IX	Copy number histogram plot
ASYG164 (*mrc1*)	Homology-mediated translocation between chr V L *YCLWdelta5* at *can1::P_{LEU2}-NAT* and chr V R "*YERWdelta27"*, an unannotated delta at chrV:449,322-449,631	Copy number (Supplementary Fig. 8a) Discordant read pairs (194 read pairs)
ASYG266 (*mrc1*)	Homology-mediated translocation between chr V L *YCLWdelta5* at *can1::P_{LEU2}-NAT* and chr III R *YCRWdelta13*	Copy number (Supplementary Fig. 8a) Discordant read pairs (172 read pairs)
	Disomy of chr IX disomy	Copy number histogram plot
ASYG267 (*mrc1*)	*de novo* telomere addition on chr V L (at 30,823 bp inside *AVT2*)	Copy number (Supplementary Fig. 8b) Junction sequence (22 reads; Supplementary Fig. 8b)
ASYG268 (*mrc1*)	Interstitial deletion on chr V L from 12,564 bp to 31,929 bp (inside *CAN1* at *yel068c::CAN1-URA3*) with 7-bp microhomology	Copy number (Supplementary Fig. 8b) Discordant read pairs (256 read pairs) Junction sequence (53 reads; Supplementary Fig. 8b)
ASYG269 (*mrc1*)	Homology-mediated translocation between chr V L *YCLWdelta5* at *can1::P_{LEU2}-NAT* and chr V R "*YERWdelta27"*, an unannotated delta at chrV:449,322-449,631	Copy number (Supplementary Fig. 8a) Discordant read pairs (128 read pairs)
ASYG187 (*mrc1-1-843*)	Homology-mediated translocation between chr V L *YCLWdelta5* at *can1::P_{LEU2}-NAT* and chr V R "*YERWdelta27"*, an unannotated delta at chrV:449,322-449,631	Copy number (Supplementary Fig. 9a) Discordant read pairs (203 read pairs)
ASYG188 (*mrc1-1-843*)	*de novo* telomere addition on chr V L (at 34,841 bp inside *NPR2*)	Copy number (Supplementary Fig. 9b) Junction sequence (29 reads; Supplementary Fig. 9b)

ASYG189 (*mrc1-1-843*)	Homology-mediated translocation between chr V L *YCLWdelta5* at *can1::*P_{LEU2}*-NAT* and chr VII R *YGRWdelta28* in *YGRCTy2-2*	Copy number (Supplementary Fig. 9a)<br>Discordant read pairs (60 read pairs)
	Homology-mediated translocation between chr VII R *YGRWTy2-2* and chrXII R *YLRCdelta21*	Copy number (Supplementary Fig. 9a)
	Homology-mediated translocation between chr XII R *YLRCTy2-2* and chr V R *YERCdelta20*	Copy number (Supplementary Fig. 9a)
ASYG190 (*mrc1-1-843*)	Homology-mediated translocation between chr V L *YCLWdelta5* at *can1::*P_{LEU2}*-NAT* and chr XII R *YGRWTy2-2*	Copy number (Supplementary Fig. 9a)
ASYG191 (*mrc1-1-843*)	Homology-mediated translocation between chr V L *YCLWdelta5* at *can1::*P_{LEU2}*-NAT* and chr IV R *YDRWTy2-3*	Copy number (Supplementary Fig. 9a)<br>Discordant read pairs (58 read pairs)
ASYG263 (*mrc1-1-843*)	Homology-mediated translocation between chr V L *SUP53* at *can1::*P_{LEU2}*-NAT* and chr XIII R *tL(CAA)M*	Copy number (Supplementary Fig. 9a)<br>Discordant read pairs (78 read pairs)
ASYG264 (*mrc1-1-843*)	Homology-mediated translocation between chr V L *YCLWdelta5* at *can1::*P_{LEU2}*-NAT* and chr IV R *YDRWTy2-3*	Copy number (Supplementary Fig. 9a)<br>Discordant read pairs (38 read pairs)
ASYG276 (*mrc1-1-843*)	Homology-mediated translocation between chr V L *YCLWdelta5* at *can1::*P_{LEU2}*-NAT* and chr X R *YJRWdelta17*	Copy number (Supplementary Fig. 9a)<br>Discordant read pairs (134 read pairs)
ASYG277 (*mrc1-1-843*)	*de novo* telomere addition on chr V L (at 36,339 bp)	Copy number (Supplementary Fig. 9b)<br>Junction sequence (37 reads; Supplementary Fig. 9b)
ASYG278 (*mrc1-1-843*)	Microhomology-containing translocation between chr V L (36,841 bp, inside *CIN8*) and chr II R (557,876 bp, inside *AMN1*) with 12 bp microhomology	Copy number (Supplementary Fig. 9b)<br>Discordant read pairs (166 read pairs)<br>Junction sequence (21 reads; Supplementary Fig. 9b)
ASYG279 (*mrc1-1-843*)	Homology-mediated translocation between chr V L *YCLWdelta5* at *can1::*P_{LEU2}*-NAT* and chr X R *YJRWdelta17*)	Copy number (Supplementary Fig. 9a)<br>Discordant read pairs (90 read pairs)
ASYG230 (*mrc1 swr1*)	Hairpin-mediated inverted duplication on chr V L (hairpin inside *SIT1*, 5-base loop, duplication until *YELWdelta1/2*)	Copy number (Supplementary Fig. 10a)<br>Discordant read pairs (54 read pairs)<br>Junction sequence (42 reads; Supplementary Fig. 12D)
	Homology-mediated translocation between chr V L *YERWdelta1/2* and chr III R *YCRWdelta11*	Copy number (Supplementary Fig. 10a)
ASYG231 (*mrc1 swr1*)	Interstitial deletion on chr V L from 18,986 bp to 31,330 bp downstream of *CAN1* at *yel068c::CAN1-URA3* with 8 bp microhomology	Copy number (Supplementary Fig. 10b)<br>Discordant read pairs (71 read pairs)<br>Junction sequence (10 reads; Supplementary Fig. 10b)
ASYG232 (*mrc1 swr1*)	*de novo* telomere addition on chr V L at 31,669 bp at *yel068c::CAN1-URA3*	Copy number (Supplementary Fig. 10b)<br>Junction sequence (10 reads; Supplementary Fig. 10b)
ASYG233 (*mrc1 swr1*)	Homology-mediated translocation between chr V L *YCLWdelta5* at *can1::*P_{LEU2}*-NAT* and chr V R "*YERWdelta27*", an unannotated delta at chrV:449,322-449,631	Copy number (Supplementary Fig. 10a)<br>Discordant read pairs (101 read pairs)
ASYG234 (*mrc1 swr1*)	Interstitial deletion on chr V L from 25,751 bp (inside *YEL068C*) to 32,347 bp (inside *CAN1* at *yelo068c::CAN1-URA3*) with 20 bp imperfect microhomology (17/20 bp identity)	Copy number (Supplementary Fig. 10b)<br>Discordant read pairs (415 read pairs)<br>Junction sequence (93 reads; Supplementary Fig. 10b)
ASYG235 (*mrc1 swr1*)	Homology-mediated translocation between chr V L *YCLWdelta5* at *can1::*P_{LEU2}*-NAT* and chr IV R *YDRWTy2-2*	Copy number (Supplementary Fig. 10a)<br>Discordant read pairs (216 read pairs)
	Homology-mediated translocation between chrIV R *YDRCTy1-2* and chrVIII R *YHRCTy1-1*	Copy number (Supplementary Fig. 10a)
ASYG236 (*mrc1 swr1*)	Homology-mediated translocation between chr V L *YCLWdelta5* at *can1::*P_{LEU2}*-NAT* and chr X L *YJLCdelta4/5*	Copy number (Supplementary Fig. 10a)<br>Discordant read pairs (127 read pairs)

ASYG237 (*mrc1 swr1*)	*de novo* telomere addition on chr V L at 28,339 bp (inside *SIT1*)	Copy number (Supplementary Fig. 10b)<br>Junction sequence (34 reads; Supplementary Fig. 10b)
	Chromosome end-fusion between chr V L *de novo* telomere and chr VI R	Copy number (Supplementary Fig. 10b)<br>Discordant read pairs (109 read pairs)
	Homology-mediated inverted duplication between chr VI R *SUP11* and chr VI R *SUP6*	Copy number (Supplementary Fig. 10b)<br>Discordant read pairs (344 read pairs)
ASYG238 (*mrc1 swr1*)	Homology-mediated translocation between chr V L *YCLWdelta5* at *can1::*P_{LEU2}*-NAT* and chr IV R *YDRWTy2-3*	Copy number (Supplementary Fig. 10a)<br>Discordant read pairs (99 read pairs)
ASYG239 (*mrc1 swr1*)	Homology-mediated translocation between chr V L *YCLWdelta5* at *can1::*P_{LEU2}*-NAT* and chr V R "*YERWdelta27*", an unannotated delta at chrV:449,322-449,631	Copy number (Supplementary Fig. 10a)<br>Discordant read pairs (181 read pairs)
ASYG200 (*mrc1-1-843 swr1*)	Hairpin-mediated inverted duplication on chr V L (hairpin inside *NAT* at *can1::*P_{LEU2}*-NAT*, 243-base loop)	Copy number (Supplementary Fig. 11a)<br>Discordant read pairs (89 read pairs)<br>Junction sequence (26 reads; Supplementary Fig. 12B)
	Homology-mediated translocation between chr V L *YELCdelta4* and chr IV R *YDRCTy1-3*	Copy number (Supplementary Fig. 11a)
ASYG201 (*mrc1-1-843 swr1*)	Hairpin-mediated inverted duplication on chr V L (hairpin inside *NAT* at *can1::*P_{LEU2}*-NAT*, 157-base loop)	Copy number (Supplementary Fig. 11a)<br>Discordant read pairs (11 read pairs)<br>Junction sequence (23 reads; Supplementary Fig. 12B)
	Homology-mediated translocation between chr V L *YELCdelta4* and chr IV R *YDRCTy1-3*	Copy number (Supplementary Fig. 11a)
ASYG202 (*mrc1-1-843 swr1*)	Interstitial deletion on chr V L from 19,129 bp to 27,085 bp (inside *HPA3*) with 8 bp microhomology	Copy number (Supplementary Fig. 11b)<br>Discordant read pairs (561 read pairs)<br>Junction sequence (95 reads; Supplementary Fig. 11b)
ASYG203 (*mrc1-1-843 swr1*)	Hairpin-mediated inverted duplication on chr V L (hairpin inside *NPR2*)	Copy number (Supplementary Fig. 11a)<br>Discordant read pairs (104 read pairs)<br>Junction sequence (72 reads; Supplementary Fig. 12A)
	Homology-mediated translocation between chr V L *YELCdelta4* and chr I R *YARCTy1-1*	Copy number (Supplementary Fig. 11a)<br>Discordant read pairs (33 read pairs)
ASYG204 (*mrc1-1-843 swr1*)	Note: Isolate has 2n genomic copy with two chrV-derived GCRs; duplications on chr V L, chr IV R, and chr VII R are all 1.5x higher than the median 2n copy number.	-
	First GCR: Hairpin-mediated inverted duplication on chr V L (hairpin inside NAT)	Copy number (Supplementary Fig. 11a)<br>Junction sequence (52 reads; Supplementary Fig. 12C)
	First GCR: Homology-mediated translocation between *YELCdelta4* and chr IV R *YDRWTy2-3/YDRWTy1-3* or chrVII *YGRWTy2-2/YGRCTy1-3*	Copy number (Supplementary Fig. 11a)
	Second GCR: Homology-mediated translocation between chr V L *YCLWdelta5* at *can1::*P_{LEU2}*-NAT* and chr IV R *YDRWTy2-3/YDRWTy1-3* or chrVII *YGRWTy2-2/YGRCTy1-3*	Copy number (Supplementary Fig. 11a)
ASYG205 (*mrc1-1-843 swr1*)	Interstitial deletion on chr V L from 116,363 bp (inside *URA3* at *yel068c::CAN1-URA3*) to 30,475 bp (inside *AVT2*) with 14 bp microhomology	Copy number (Supplementary Fig. 11b)<br>Discordant read pairs (626 read pairs)<br>Junction sequence (68 reads; Supplementary Fig. 11b)
ASYG206 (*mrc1-1-843 swr1*)	Homology-mediated translocation between chr V L *SUP53* at *can1::*P_{LEU2}*-NAT* and chr XIII R *tL(CAA)M*	Copy number (Supplementary Fig. 11b)<br>Discordant read pairs (171 read pairs)
ASYG208 (*mrc1-1-843 swr1*)	Homology-mediated translocation between chr V L *YCLWdelta5* at *can1::*P_{LEU2}*-NAT* and chr IV R *YDRWTy2-3*	Copy number (Supplementary Fig. 11b)<br>Discordant read pairs (71 read pairs)

	Homology-mediated translocation between chr IV R *YDRWTy2-3* and chr XV R *YORWTy2-2*	Copy number (Supplementary Fig. 11b)
ASYG209 (*mrc1-1-843 swr1*)	Note: Isolate has 4 copies of the sequence between hairpin-mediated inverted duplication and *YELCdelta4*, 2 copies of the sequence between *YELCdelta4* and *YELWdelta6*, and two copies of the sequence telomeric to *YERCTy1-1*.	-
	Hairpin-mediated inverted duplication on chr V L (hairpin inside *SIT1*, 45-base loop)	Copy number (Supplementary Fig. 11a)<br>Discordant read pairs (238 read pairs)<br>Junction sequence (165 reads; Supplementary Fig. 12C)
	Homology-mediated inverted duplication on chr V L (mediated by pairing between *YELWdelta6* and *YELCdelta4*)	Copy number (Supplementary Fig. 11a)<br>Discordant read pairs (19 read pairs)
	Hairpin-mediated inverted duplication on chr V L (hairpin inside *SIT1*, 45-base loop)	Copy number (Supplementary Fig. 11a)<br>Discordant read pairs (238 read pairs)<br>Junction sequence (165 reads; Supplementary Fig. 12C)
	Homology-mediated translocation between chr V L *YELCdelta4* and chr V R *YERCTy1-1*	Copy number (Supplementary Fig. 11a)
ASYG210 (*mrc1-1-843 swr1*)	Hairpin-mediated inverted duplication on chr V L (hairpin inside *PRB1*, 7-base loop)	Copy number (Supplementary Fig. 11a)<br>Discordant read pairs (73 read pairs)<br>Junction sequence (85 reads; Supplementary Fig. 12D)
	Homology-mediated translocation between chr V L *YELCdelta4* and chr IV R *YDRCTy1-3*	Copy number (Supplementary Fig. 11a)

[1]Evidence supporting GCR-related junctions can be derived from whole genome sequencing data in several ways. Frequently, multiple lines of evidence support many junctions. Copy number changes, from normalized read depth plots of uniquely mapping regions of the genome, can suggest possible rearrangements, particularly those involving HR between repetitive elements. Discordant read pairs are read pairs where two uniquely mapping reads map to regions consistent with the GCR, but not with reference chromosome; note that the number of discordant read pairs is influenced by whether the rearrangement is within or adjacent to non-uniquely mapping regions of the genome. Junction sequences can be determined in two ways, depending on the junction. For junctions that generate a novel sequence, such as a deletion or hairpin-mediated inversion, the sequence can be derived from read pairs adjacent to junction-defining discordant read pairs in which one read does not map to the reference and potentially sequences the novel junction. In these cases, the numbers of reads that align to define the novel junction sequence are reported. For junctions mediated by homologies, such as tRNA genes or Ty-related sequences, sequences can be determined by aligning reads that are linked to uniquely mapping regions adjacent to the homologies involved in the junction.

[2]Translocation observed by junction-defining read pairs for the translocation target with both ChrV L (adjacent to P_{LEU2}-*YCLWdelta5* fragment, listed first) and ChrIII L (within the P_{LEU2}-*YCLWdelta5* fragment, listed second).

Supplementary Table 6. *S. cerevisiae* strains used in this study

Strain	Genotype*	Reference
RDKY7629	*MAT*alpha *hom3-10 ura3-Δ0 leu2-Δ0 trp1-Δ63 his3-Δ200 lyp1Δ::TRP1 cyh2-Q38K iYFR016c::*P_{MFA1}*-LEU2 can1Δ::*$P_{LEU2(SUP53,YCLWdelta5)}$*-natNT2*	Ref. 2
RDKY8624	*MAT*alpha *hom3-10 ura3-Δ0 leu2-Δ0 trp1-Δ63 his3-Δ200 lyp1Δ::TRP1 cyh2-Q38K iYFR016c::*P_{MFA1}*-LEU2 can1Δ::*P_{LEU2}*-natNT2*	Ref. 2
RDKY7635	*MAT*alpha *hom3-10 ura3-Δ0 leu2-Δ0 trp1-Δ63 his3-Δ200 lyp1Δ::TRP1 cyh2-Q38K iYFR016c::*P_{MFA1}*-LEU2 can1Δ::*$P_{LEU2(SUP53,YCLWdelta5)}$*-natNT2 yel072w::CAN1-URA3*	Ref. 2
RDKY7964	*MAT*alpha *hom3-10 ura3-Δ0 leu2-Δ0 trp1-Δ63 his3-Δ200 lyp1Δ::TRP1 cyh2-Q38K iYFR016c::*P_{MFA1}*-LEU2 can1Δ::*$P_{LEU2(SUP53,YCLWdelta5)}$*-natNT2 yel068c::CAN1-URA3*	Ref. 2
RDKY8625	*MAT*alpha *hom3-10 ura3-Δ0 leu2-Δ0 trp1-Δ63 his3-Δ200 lyp1Δ::TRP1 cyh2-Q38K iYFR016c::*P_{MFA1}*-LEU2 can1Δ::*P_{LEU2}*-natNT2 yel068c::CAN1-URA3*	Ref. 2
RDKY7785	RDKY7635 *swr1Δ::HIS3*	Ref. 2
RDKY9077	RDKY7964 *swr1Δ::HIS3*	this study
RDKY8808	RDKY8625 *swr1Δ::HIS3*	this study
RDKY8969	RDKY7635 *htz1Δ::HIS3*	this study
RDKY9079	RDKY7964 *htz1Δ::HIS3*	this study
RDKY8810	RDKY8625 *htz1Δ::HIS3*	this study
RDKY8301	RDKY7635 *mrc1Δ::kanMX4*	this study
RDKY9081	RDKY7964 *mrc1Δ::kanMX4*	this study
RDKY8804	RDKY8625 *mrc1Δ::kanMX4*	this study
RDKY8302	RDKY7635 *mrc1Δ::kanMX4 swr1Δ::HIS3*	this study
RDKY9083	RDKY7964 *mrc1Δ::kanMX4 swr1Δ::HIS3*	this study
RDKY9085	RDKY8625 *mrc1Δ::kanMX4 swr1Δ::HIS3*	this study
RDKY8975	RDKY7635 *mrc1Δ::kanMX4 htz1Δ::HIS3*	this study
RDKY9087	RDKY8625 *mrc1Δ::kanMX4 htz1Δ::HIS3*	this study
RDKY9291	RDKY7635 *vps71Δ::HIS3*	this study
RDKY9293	RDKY7635 *vps72Δ::HIS3*	this study
RDKY9295	RDKY7635 *swc3Δ::HIS3*	this study
RDKY9297	RDKY7635 *swc5Δ::HIS3*	this study
RDKY9299	RDKY7635 *arp6Δ::HIS3*	this study
RDKY9301	RDKY7635 *yaf9Δ::HIS3*	this study
RDKY9303	RDKY7635 *mrc1Δ::kanMX4 vps71Δ::HIS3*	this study
RDKY9305	RDKY7635 *mrc1Δ::kanMX4 vps72Δ::HIS3*	this study
RDKY9307	RDKY7635 *mrc1Δ::kanMX4 swc3Δ::HIS3*	this study
RDKY9309	RDKY7635 *mrc1Δ::kanMX4 swc5Δ::HIS3*	this study
RDKY9311	RDKY7635 *mrc1Δ::kanMX4 arp6Δ::HIS3*	this study
RDKY9313	RDKY7635 *mrc1Δ::kanMX4 yaf9Δ::HIS3*	this study
RDKY8304	RDKY7629 *mrc1-aq.kanMX4*	this study
RDKY8305	RDKY7635 *mrc1-aq.kanMX4*	this study

RDKY9089	RDKY7964 *mrc1-aq.kanMX4*	this study
RDKY8818	RDKY8624 *mrc1-aq.kanMX4*	this study
RDKY9091	RDKY8625 *mrc1-aq.kanMX4*	this study
RDKY8306	RDKY7635 *mrc1-aq.kanMX4 swr1Δ::HIS3*	this study
RDKY9093	RDKY7964 *mrc1-aq.kanMX4 swr1Δ::HIS3*	this study
RDKY9095	RDKY8625 *mrc1-aq.kanMX4 swr1Δ::HIS3*	this study
RDKY9097	RDKY7635 *mrc1-aq.kanMX4 htz1Δ::HIS3*	this study
RDKY9099	RDKY7964 *mrc1-aq.kanMX4 htz1Δ::HIS3*	this study
RDKY9101	RDKY8625 *mrc1-aq.kanMX4 htz1Δ::HIS3*	this study
RDKY9315	RDKY7635 *mrc1-aq.kanMX4 vps71Δ::HIS3*	this study
RDKY9317	RDKY7635 *mrc1-aq.kanMX4 vps72Δ::HIS3*	this study
RDKY9319	RDKY7635 *mrc1-aq.kanMX4 swc3Δ::HIS3*	this study
RDKY9321	RDKY7635 *mrc1-aq.kanMX4 swc5Δ::HIS3*	this study
RDKY9323	RDKY7635 *mrc1-aq.kanMX4 arp6Δ::HIS3*	this study
RDKY9325	RDKY7635 *mrc1-aq.kanMX4 yaf9Δ::HIS3*	this study
RDKY8967	RDKY7635 *mrc1-1-843.kanMX4*	this study
RDKY9102	RDKY7964 *mrc1-1-843.kanMX4*	this study
RDKY8814	RDKY8625 *mrc1-1-843.kanMX4*	this study
RDKY8973	RDKY7635 *mrc1-1-843.kanMX4 swr1Δ::HIS3*	this study
RDKY9104	RDKY7964 *mrc1-1-843.kanMX4 swr1Δ::HIS3*	this study
RDKY9106	RDKY8625 *mrc1-1-843.kanMX4 swr1Δ::HIS3*	this study
RDKY9108	RDKY7635 *mrc1-1-843.kanMX4 htz1Δ::HIS3*	this study
RDKY9110	RDKY7964 *mrc1-1-843.kanMX4 htz1Δ::HIS3*	this study
RDKY9112	RDKY8625 *mrc1-1-843.kanMX4 htz1Δ::HIS3*	this study
RDKY8963	RDKY7635 *tof1Δ::kanMX4*	this study
RDKY9114	RDKY7964 *tof1Δ::kanMX4*	this study
RDKY8816	RDKY8625 *tof1Δ::kanMX4*	this study
RDKY8971	RDKY7635 *tof1Δ::kanMX4 swr1Δ::HIS3*	this study
RDKY9115	RDKY7964 *tof1Δ::kanMX4 swr1Δ::HIS3*	this study
RDKY9117	RDKY8625 *tof1Δ::kanMX4 swr1Δ::HIS3*	this study
RDKY9119	RDKY7635 *tof1Δ::kanMX4 htz1Δ::HIS3*	this study
RDKY9121	RDKY8625 *tof1Δ::kanMX4 htz1Δ::HIS3*	this study
RDKY8965	RDKY7635 *csm3Δ::kanMX4*	this study
RDKY9128	RDKY7964 *csm3Δ::kanMX4*	this study
RDKY8806	RDKY8625 *csm3Δ::kanMX4*	this study
RDKY8972	RDKY7635 *csm3Δ::kanMX4 swr1Δ::HIS3*	this study
RDKY9165	RDKY7635 *csm3Δ::hphNT1 swr1Δ::HIS3*	this study
RDKY9130	RDKY7964 *csm3Δ::kanMX4 swr1Δ::HIS3*	this study
RDKY9132	RDKY8625 *csm3Δ::kanMX4 swr1Δ::HIS3*	this study
RDKY9134	RDKY7635 *csm3Δ::kanMX4 htz1Δ::HIS3*	this study
RDKY9136	RDKY8625 *csm3Δ::kanMX4 htz1Δ::HIS3*	this study

RDKY9138	RDKY7635 *swr1Δ::HIS3 htz1Δ::hphNT1*	this study
RDKY9140	RDKY7635 *mrc1Δ::kanMX4 swr1Δ::HIS3 htz1Δ::hphNT1*	this study
RDKY9141	RDKY7635 *mrc1Δ::kanMX4 tof1Δ::hphNT1*	this study
RDKY9143	RDKY7635 *mrc1Δ::kanMX4 swr1Δ::HIS3 tof1Δ::hphNT1*	this study
RDKY9145	RDKY7635 *mrc1-aq.kanMX4 tof1Δ::hphNT1*	this study
RDKY9147	RDKY7635 *mrc1-aq.kanMX4 swr1Δ::HIS3 tof1Δ::hphNT1*	this study
RDKY9150	RDKY7635 *mrc1-1-843.kanMX4 tof1Δ::hphNT1*	this study
RDKY9152	RDKY7635 *mrc1-1-843.kanMX4 swr1Δ::HIS3 tof1Δ::hphNT1*	this study
RDKY9154	RDKY7635 *mrc1::kanMX4 csm3Δ::hphNT1*	this study
RDKY9156	RDKY7635 *mrc1Δ::kanMX4 swr1Δ::HIS3 csm3Δ::hphNT1*	this study
RDKY9158	RDKY7635 *mrc1-aq.kanMX4 csm3Δ::hphNT1*	this study
RDKY9160	RDKY7635 *mrc1-aq.kanMX4 swr1Δ::HIS3 csm3Δ::hphNT1*	this study
RDKY9163	RDKY7635 *mrc1-1-843.kanMX4 csm3Δ::hphNT1*	this study
RDKY9166	RDKY7635 *mrc1-1-843.kanMX4 swr1Δ::HIS3 csm3Δ::hphNT1*	this study
RDKY9167	RDKY7635 *HTZ1.HIS3*	this study
RDKY9169	RDKY7635 *htz1-K126R,K133R.HIS3*	this study
RDKY9171	RDKY7635 *mrc1Δ::kanMX4 htz1-K126R,K133R.HIS3*	this study
RDKY9173	RDKY7635 *tof1Δ::kanMX4 htz1-K126R,K133R.HIS3*	this study
RDKY9174	RDKY7635 *csm3Δ::kanMX4 htz1-K126R,K133R.HIS3*	this study
RDKY9248	RDKY7635 *htz1-K(4,9,11,15)R.HIS3*	this study
RDKY9256	RDKY7635 *mrc1Δ::kanMX4 htz1-K(4,9,11,15)R.HIS3*	this study
RDKY9251	RDKY7635 *htz1-K(4,9,11,15)Q.HIS3*	this study
RDKY9258	RDKY7635 *mrc1Δ::kanMX4 htz1-K(4,9,11,15)Q.HIS3*	this study
RDKY9260	RDKY7635 *htz1-K(4,9,11,15,126,133)R.HIS3*	this study
RDKY9263	RDKY7635 *mrc1Δ::kanMX4 htz1-K(4,9,11,15,126,133)R.HIS3*	this study
RDKY9265	RDKY7635 *htz1-1-114.HIS3*	this study
RDKY9269	RDKY7635 *mrc1Δ::kanMX4 htz1—1-114.HIS3*	this study
RDKY9267	RDKY7635 *htz1-1-120.HIS3*	this study
RDKY9271	RDKY7635 *mrc1Δ::kanMX4 htz1—1-120.HIS3*	this study
RDKY9176	RDKY8625 *rad52Δ::hphNT1*	this study
RDKY9178	RDKY8625 *mrc1Δ::kanMX4 rad52Δ::hphNT1*	this study
RDKY9180	RDKY8625 *swr1Δ::HIS3 rad52Δ::hphNT1*	this study
RDKY9182	RDKY8625 *mrc1Δ::kanMX4 swr1Δ::HIS3 rad52Δ::hphNT1*	this study
RDKY9184	RDKY8625 *ino80Δ::hphNT1*	this study
RDKY9186	RDKY8625 *mrc1Δ::kanMX4 ino80Δ::hphNT1*	this study
RDKY9188	RDKY8625 *swr1Δ::HIS3 ino80Δ::hphNT1*	this study
RDKY9190	RDKY8625 *mrc1Δ::kanMX4 swr1Δ::HIS3 ino80Δ::hphNT1*	this study
RDKY9273	RDKY8625 *htz1Δ::HIS3 ino80Δ::hphNT1*	this study
RDKY9275	RDKY8625 *mrc1Δ::kanMX4 htz1Δ::HIS3 ino80Δ::hphNT1*	this study
RDKY8977	RDKY7635 *DDC2-EGFP.hphNT1*	this study
RDKY8978	RDKY7635 *mrc1Δ::kanMX4 DDC2-EGFP.hphNT1*	this study

RDKY8979	RDKY7635 *swr1Δ::HIS3 DDC2-EGFP.hphNT1*	this study
RDKY8980	RDKY7635 *mrc1Δ::kanMX4 swr1Δ::HIS3 DDC2-EGFP.hphNT1*	this study
RDKY8983	RDKY7635 *mrc1-1-843.kanMX4 DDC2-EGFP.hphNT1*	this study
RDKY8987	RDKY7635 *mrc1-1-843.kanMX4 swr1Δ::HIS3 DDC2-EGFP.hphNT1*	this study
RDKY9277	RDKY7635 *mrc1-aq.kanMX4 DDC2-EGFP.hphNT1*	this study
RDKY9279	RDKY7635 *mrc1-aq.kanMX4 swr1Δ::HIS3 DDC2-EGFP.hphNT1*	this study
RDKY8981	RDKY7635 *tof1Δ::kanMX4 DDC2-EGFP.hphNT1*	this study
RDKY8985	RDKY7635 *tof1Δ::kanMX4 swr1Δ::HIS3 DDC2-EGFP.hphNT1*	this study
RDKY8982	RDKY7635 *csm3Δ::kanMX4 DDC2-EGFP.hphNT1*	this study
RDKY8986	RDKY7635 *csm3Δ::kanMX4 swr1Δ::HIS3 DDC2-EGFP.hphNT1*	this study
RDKY8984	RDKY7635 *htz1Δ::HIS3 DDC2-EGFP.hphNT1*	this study
RDKY8988	RDKY7635 *mrc1Δ::kanMX4 htz1Δ::HIS3 DDC2-EGFP.hphNT1*	this study
RDKY8989	RDKY7635 *HUG1-EGFP.hphNT1*	this study
RDKY8991	RDKY7635 *mrc1Δ::kanMX4 HUG1-EGFP.hphNT1*	this study
RDKY8993	RDKY7635 *swr1Δ::HIS3 HUG1-EGFP.hphNT1*	this study
RDKY8995	RDKY7635 *mrc1Δ::kanMX4 swr1Δ::HIS3 HUG1-EGFP.hphNT1*	this study
RDKY8997	RDKY7635 *tof1Δ::kanMX4 HUG1-EGFP.hphNT1*	this study
RDKY8999	RDKY7635 *csm3Δ::kanMX4 HUG1-EGFP.hphNT1*	this study
RDKY9394	RDKY7635 *mrc1-1-843.kanMX4 HUG1-EGFP.hphNT1*	this study
RDKY9003	RDKY7635 *htz1Δ::HIS3 HUG1-EGFP.hphNT1*	this study
RDKY9005	RDKY7635 *tof1Δ::kanMX4 swr1Δ::HIS3 HUG1-EGFP.hphNT1*	this study
RDKY9007	RDKY7635 *csm3Δ::kanMX4 swr1Δ::HIS3 HUG1-EGFP.hphNT1*	this study
RDKY9009	RDKY7635 *mrc1-1-843.kanMX4 swr1Δ::HIS3 HUG1-EGFP.hphNT1*	this study
RDKY9011	RDKY7635 *mrc1Δ::kanMX4 htz1Δ::HIS3 HUG1-EGFP.hphNT1*	this study
RDKY9281	RDKY7635 *mrc1-aq.kanMX4 HUG1-EGFP.hphNT1*	this study
RDKY9283	RDKY7635 *mrc1-aq.kanMX4 swr1Δ::HIS3 HUG1-EGFP.hphNT1*	this study
BY4741	*MAT***a** *his3-Δ1 leu2-Δ0 met15-Δ0 ura3-Δ0*	Ref. 3
RDKY9285	BY4741 *mrc1-1-843.kanMX4*	this study
RDKY9286, RDKY9287	RDKY7635 *mrc1-1-843.kanMX4 rad9Δ::HIS3*	this study
RDKY9288, RDKY9289, RDKY9290	RDKY7964 *mrc1-1-843.kanMX4 rad9Δ::HIS3*	this study
FY1296	W303 *MAT***a** *ade2-1 trp1-1 leu2-3,112 his3-11,15 ura3 can1-100 RAD5*$^+$	Ref. 4
HY3777	W303 *MAT***a** *ade2-1 trp1-1 leu2-3,112 his3-11,15 ura3 can1-100 RAD5*$^+$ *mrc1Δ::HIS5*	this study
RDKY8798	W303 *MAT***a** *ade2-1 trp1-1 leu2-3,112 his3-11,15 ura3 can1-100 RAD5*$^+$ *swr1Δ::kanMX4*	this study
RDKY8800	W303 *MAT***a** *ade2-1 trp1-1 leu2-3,112 his3-11,15 ura3 can1-100 RAD5*$^+$ *htz1Δ::kanMX4*	this study
RDKY8801	W303 *MAT***a** *ade2-1 trp1-1 leu2-3,112 his3-11,15 ura3 can1-100 RAD5*$^+$ *mrc1Δ::HIS5 swr1Δ::kanMX4*	this study
RDKY8802	W303 *MAT***a** *ade2-1 trp1-1 leu2-3,112 his3-11,15 ura3 can1-100 RAD5*$^+$ *tof1Δ::HIS5*	this study

RDKY8820	W303 *MAT***a** *ade2-1 trp1-1 leu2-3,112 his3-11,15 ura3 can1-100 RAD5*$^{+}$ *mrc1Δ::HIS5 htz1Δ::kanMX4*	this study
RDKY8822	W303 *MAT***a** *ade2-1 trp1-1 leu2-3,112 his3-11,15 ura3 can1-100 RAD5*$^{+}$ *tof1Δ::HIS5 swr1Δ::kanMX4*	this study
RDKY8824	W303 *MAT***a** *ade2-1 trp1-1 leu2-3,112 his3-11,15 ura3 can1-100 RAD5*$^{+}$ *tof1Δ::HIS5 htz1Δ::kanMX4*	this study

* Strains are isogenic to S288c unless otherwise indicated

Supplementary Table 7. Primers used in this study

Name	Sequence (5'-3')	Purpose
oAS145	TTCTAACTGCTCTTTGCATTTTCCAAGTTATTGCATTACAAGAATATATGgagcagattgtactgagagtgcacc	Amplify *HIS3* from *pRS303* to delete *SWR1*
oAS146	TCCGATTTGGACAACTAAGGCAGCGGTGAAGAGTAGAACCTGGTCCTTCActccttacgcatctgtgcggtatttc	
oAS659	TTCTAACTGCTCTTTGCATTTTCCAAGTTATTGCATTACAAGAATATATGcgtacgctgcaggtcgac	Amplify *kanMX4* from *pFA6a-kanMX4* to delete *SWR1*
oAS660	TCCGATTTGGACAACTAAGGCAGCGGTGAAGAGTAGAACCTGGTCCTTCAatcgatgaattcgagctcgt	
oAS147	CCAAAGCATTCGATTACTGC	Verify *swr1Δ*
oAS148	GGCGATAGTGTTGTCGGAAA	
oAS323	AGGAAGTTCGTTATTCGCTTTTGAACTTATCACCAAATATTTTAGTGATGcgtacgctgcaggtcgac	Amplify *kanMX4* from *pFA6a-kanMX4* to delete *MRC1*
oAS324	ACAGCTTCTGGAGTTCAATCAACTTCTTCGGAAAAGATAAAAAACCACTAatcgatgaattcgagctcgt	
oAS325	GCTCCGGAAGAAGAAGCA	Verify *mrc1Δ*
oAS326	GGGGAGAAAGAATAAGGGCA	
oAS329	CTAGCTTGTGGGGTTTAGTGTATCTTTAATATAGGAGGGCGCACACTATGcgtacgctgcaggtcgac	Amplify *kanMX4* from *pFA6a-kanMX4* to delete *TOF1*
oAS330	TTCTAAAATTACACGTATTAAAGGGATTAATTACTACATATTCATTCTCAatcgatgaattcgagctcgt	
oAS500	CTAGCTTGTGGGGTTTAGTGTATCTTTAATATAGGAGGGCGCACACTATGcgtacgctgcaggtcgac	Amplify *hphNT1* from *pFA6a-hphNT1* to delete *TOF1*
oAS501	TTCTAAAATTACACGTATTAAAGGGATTAATTACTACATATTCATTCTCAatcgatgaattcgagctcg	
oAS331	GAATTGCTTCCCTGTGGAAA	Verify *tof1Δ*
oAS332	TGATAAAGAATGAATCGCATGT	
oAS356	TGGATTAAAATGCCATGAAAACGTGAACAGAAACTTTTATTGAGGTCATGcgtacgctgcaggtcgac	Amplify *kanMX4* from *pFA6a-kanMX4* to delete *CSM3*
oAS357	TATAGATGCCCACACGCACGTTTGGATTATTACCTTCAATGACATTGCTAatcgatgaattcgagctcgt	
oAS502	TGGATTAAAATGCCATGAAAACGTGAACAGAAACTTTTATTGAGGTCATGcgtacgctgcaggtcgac	Amplify *hphNT1* from *pFA6a-hphNT1* to delete *CSM3*
oAS503	TATAGATGCCCACACGCACGTTTGGATTATTACCTTCAATGACATTGCTAatcgatgaattcgagctcg	
oAS358	AAGGAAAAATGCCGGGTAAT	Verify *csm3Δ*
oAS359	TCGAACCAGGCTCTTTCTACA	
oAS362	TTGATAGATCGAAGAAAAAGAACGGATCGCTTACTCATAACAGCGAAATGgagcagattgtactgagagtgcacc	Amplify *HIS3* from *pRS303* to delete *VPS71*
oAS363	TATAAATAGGGGAGAAAAAAGGGCTTACGAATACATACTGATCATTACTActccttacgcatctgtgcggtatttc	
oAS364	TGAACATGATGATCTTTGTGAGT	Verify *vps71Δ*
oAS365	CCTTGGAAAATGAGAATAAACGA	
oAS366	TAATGAATACAAATAGAAGTGAAACCGAAGCAAATTCACAGTGCACGATGgagcagattgtactgagagtgcacc	Amplify *HIS3* from *pRS303* to delete *VPS72*
oAS367	AATATAGTCAAATGTTTAACGACCTACCATTATATAGTCTGTCTCAATTActccttacgcatctgtgcggtatttc	

oAS368	CAGTAAGCAGAAAGCACCATGT	Verify *vps72Δ*
oAS369	CCAGAAATTGATGAATGTAACGA	
oAS370	GCATATGCATGCGATTTGGAAGTAACGCTCGCCGTAGACAAGTAAGAATGgagcagattgtactgagagtgcacc	Amplify *HIS3* from *pRS303* to delete *SWC3*
oAS371	GTGTATAATCATAATGGCGTTAAAGCAGAATAAAGTAACCGAACACCTTActccttacgcatctgtgcggtatttc	
oAS372	CTGTGCTCAATCAATCGTGTT	Verify *swc3Δ*
oAS373	CGGCAAAGTTTGGCATACA	
oAS374	AAACTGGAATAATATTGTTTCAAGAAAGAAAACCCACTAGCCTATAAATGgagcagattgtactgagagtgcacc	Amplify *HIS3* from *pRS303* to delete *SWC5*
oAS375	AAAAATTTGAGCAGAAAAGCATGTTATTTAATACATGTAATATTTGTCTActccttacgcatctgtgcggtatttc	
oAS376	CCATATCGGGGAAAGACAAA	Verify *swc5Δ*
oAS377	CACACCCATACACCCCATTA	
oAS378	GACCAAAGGAGAAGAAGCGCAGAATCGTGGACAAGATAATTTAAACTATGgagcagattgtactgagagtgcacc	Amplify *HIS3* from *pRS303* to delete *ARP6*
oAS379	TCTCGACTTTTCGGATTTTCATTGTTGCTATGTAAATCTATAGTTAATTActccttacgcatctgtgcggtatttc	
oAS380	CTTTCCGTCCATAATTACAAATG	Verify *arp6Δ*
oAS381	TTTGGGAATTTGTCAATAAAAATGT	
oAS382	ATACATTACTTGTGACCACCTATTTACGGCATCACAAAGAAAGCGAGATGgagcagattgtactgagagtgcacc	Amplify *HIS3* from *pRS303* to delete *YAF9*
oAS383	ATTACTATGGCTGTTATGAAAATACCGTTGTTCCGGGTGCAGTGATCCTActccttacgcatctgtgcggtatttc	
oAS384	ATGACGTGATGACGCTTCG	Verify *yaf9Δ*
oAS385	TATGACTCTGCACACATTTCG	
oAS386	TAAATTCAATTTCGCACTATAGCCGCACGTAAAAATAACTTAACATAATGgagcagattgtactgagagtgcacc	Amplify *HIS3* from *pRS303* to delete *HTZ1*
oAS387	CAGGAGCAGGGAGAATTACGGAAATGGGAAAGAAAAACTATTCTTCTTActccttacgcatctgtgcggtatttc	
oAS807	TAAATTCAATTTCGCACTATAGCCGCACGTAAAAATAACTTAACATAATGcgtacgctgcaggtcgac	Amplify *hphNT1* from *pFA6a-hphNT1* to delete *HTZ1*
oAS808	CAGGAGCAGGGAGAATTACGGAAATGGGAAAGAAAAACTATTCTTCTTAatcgatgaattcgagctcg	
oAS661	TAAATTCAATTTCGCACTATAGCCGCACGTAAAAATAACTTAACATAATGcgtacgctgcaggtcgac	Amplify *kanMX4* from *pFA6a-kanMX4* to delete *HTZ1*
oAS662	CAGGAGCAGGGAGAATTACGGAAATGGGAAAGAAAAACTATTCTTCTTAatcgatgaattcgagctcgt	
oAS388	AACAGCTGAACTATTATCCAGTGTAA	Verify *htz1Δ*
oAS389	CTCGGGTACGGAGAGACAAA	
oAS498	GAAAAAGGAGATTAGAAATTGGCGATGATGCAAAGCTTGTTAAAAACCCAcgtacgctgcaggtcgac	Amplify *kanMX4* from *pFA6a-kanMX4* to generate *mrc1-1-843.kanMX4*
oAS516	ACAGCTTCTGGAGTTCAATCAACTTCTTCGGAAAAGATAAAAAACCACTAatcgatgaattcgagctcg	
oAS347	AAAATGGAGGATTTCGTA	Verify *mrc1-1-843.kanMX4*
oAS517	TCTTCTTTTGCTCCAAATGTTTA	
oAS283	tgcgcaactgttgggaag	Amplify *mrc1-aq.kanMX*4 from *pRDK1779*

oAS412	GGCTCCTAGTCGTAATGGTCCTCGTATTTCAAGTTCTAAGGCGTTGTTATttatgcttccggctcctatg	
oAS415	CTCAATGCAGGGACGAAAAACAG	Amplify *CAN1-URA3* from *pRDK1378* to insert at *YEL072W*
oAS416	GCGCTCTCCAAACTAAGGGGGTTG	
oAS455	TGAATGTCGGGATAATTTGC	Amplify *CAN1-URA3* from *pRDK1379* to insert at *YEL068C*
oAS456	GCAGCGAGCCTTAGTTCATC	
oAS970	GATCAGGGCTACAATTGCTTCAGGTGGTGTTTTGCCTCATATAAATAAAGCATTATTATTGAGAGTGGAAAAAAAGGGAAGTAGGAAATAAgagcagattgtactgagagtgcacc	Amplify *HIS3* from *pRS303* to introduce *HIS3* downstream of *HTZ1* and introduce K126R and K133R
oAS972	ATACAGGAGCAGGGAGAATTACGGGAAATGGGAAAGAAAAACTATTCTTCctccttacgcatctgtgcggtatttc	
oAS971	GATCAGGGCTACAATTGCTTCAGGTGGTGTTTTGCCTCATATAAATAAAGCATTATTATTGAAAGTGGAAAAAAAGGGAAGTAAGAAATAAgagcagattgtactgagagtgcacc	Amplify *HIS3* from *pRS303* to introduce *HIS3* downstream of *HTZ1*; use with oAS972
oAS1374	TTAGAGGTGACGATGAGTTAGATTCTTTGATCAGGGCTACAATTGCTTCAGGTGGTTAAgagcagattgtactgagagtgcacc	Amplify *HIS3* from *pRS303* to introduce *HIS3* downstream of *HTZ1* and generate *htz1-1-114.HIS3*; use with oAS972
oAS1375	TAGATTCTTTGATCAGGGCTACAATTGCTTCAGGTGGTGTTTTGCCTCATATAAATTAAgagcagattgtactgagagtgcacc	Amplify *HIS3* from *pRS303* to introduce *HIS3* downstream of *HTZ1* and generate *htz1-1-120.HIS3*; use with oAS972
oAS1352	gtggaagcttATGTCAGGAAAAGCTCATGG	Amplify *HTZ1* to clone into *pFA6a-hphNT1*
oAS1353	gtgggtcgacTTATTTCTTACTTCCCTTTTTTTCCAC	
oAS1354	gcggccgccagctgaagcttATGTCAGGAAGAGCTCATGGAGGTAGAGGTAGATCCGGCGCTAGAGACAGTGGTTCATTGAGATC	Site-directed mutagenesis to generate *htz1-K4,9,11,15R*
oAS1355	GATCTCAATGAACCACTGTCTCTAGCGCCGGATCTACCTCTACCTCCATGAGCTCTTCCTGACATaagcttcagctggcggccgc	
oAS1356	gcggccgccagctgaagcttATGTCAGGACAAGCTCATGGAGGTCAAGGTCAATCCGGCGCTCAAGACAGTGGTTCATTGAGATC	Site-directed mutagenesis to generate *htz1-K4,9,11,15Q*
oAS1357	GATCTCAATGAACCACTGTCTTGAGCGCCGGATTGACCTTGACCTCCATGAGCTTGTCCTGACATaagcttcagctggcggccgc	
oAS1359	TAAATTCAATTTCGCACTATAGCCGCACGTAAAAATAACTTAACATAATGTCAGGAAGAGCTCATGGAGGTAGAG	Amplify *htz1-K4,9,11,15R.HPH* from *pRDK1835* for genomic insertion
oAS1360	TAAATTCAATTTCGCACTATAGCCGCACGTAAAAATAACTTAACATAATGTCAGGACAAGCTCATGGAGGTCAAG	Amplify *htz1-K4,9,11,15Q.HPH* from *pRDK1836* for genomic insertion
oAS1361	CAGGAGCAGGGAGAATTACGGGAAATGGGAAAGAAAAACTATTCTTCTTAatcgatgaattcgagctcg	Reverse primer for oAS1359 and oAS1360
oAS518	GGTTGCCAAGAACTGCTGAAGGTTCTGGTGGCTTTGGTGTGTTGTTGATGcgtacgctgcaggtcgac	Amplify *hphNT1* from *pFA6a-hphNT1* to delete *RAD52*
oAS519	AATAAATAATGATGCAAATTTTTTATTTGTTTCGGCCAGGAAGCGTTTCAatcgatgaattcgagctcg	
oAS33	ATCGAATGGCGTTTTTAAGC	Verify *rad52Δ*
oAS34	CGACACATGGAGGAAAGAAAA	
BZP458	ATCTAACCACACTAGAGGAGGCCGATTCATTATATATCTCAATGGGACTGcgtacgctgcaggtcgac	Amplify *EGFP.hphNT1* from *pYM25* to tag *DDC2*
BZP459	ACAAGGTTTCTATAAAGCGTTGACATTTTCCCCTTTTGATTGTTGCCTTAatcgatgaattcgagctcg	
BZP475	GAACGGGGAGAATAATGCAA	Verify *DDC2-EGFP.hph* tag

BZP476	TTGCATTATTCTCCCCGTTC	
BZP14	CCGTCGAACGTCGCGGCGGTCTTTCTGACATTGGTAAG AATACTTCCAACcgtacgctgcaggtcgac	Amplify *EGFP.hphNT1* from *pYM25* to tag *HUG1*
BZP15	TTGTTCTTTCCTATCATTGGCCTACAAAAAAAAAGAGAA GCATGCTCTTAatcgatgaattcgagctcg	
BZP18	AGCAATTCTTCCTTGACGATGT	Verify *HUG1-EGFP.hph* tag
BZP19	CGATGTTCCAAAACAGTAACGA	
oAS31	AACGCCATAGAAAAGAGCATAGTGAGAAAATCTTCAAC ATCAGGGCTATGgagcagattgtactgagagtgcacc	Amplify *HIS3* from *pRS303* to delete *RAD9*
oAS32	TTAATCGTCCCTTTCTATCAATTATGAGTTTATATATTTT TATAATTTCActccttacgcatctgtgcggtatttc	
oAS49	ACACGCGAGGATTTTTGTTC	Verify *rad9Δ*
oAS50	CGTGTGGGAGGATGTTCTTAG	

Supplementary References

1. Naylor ML, Li JM, Osborn AJ, Elledge SJ. Mrc1 phosphorylation in response to DNA replication stress is required for Mec1 accumulation at the stalled fork. *Proc Natl Acad Sci U S A* **106**, 12765-12770 (2009).

2. Putnam CD*, et al.* A genetic network that suppresses genome rearrangements in Saccharomyces cerevisiae and contains defects in cancers. *Nat Commun* **7**, 11256 (2016).

3. Brachmann CB*, et al.* Designer deletion strains derived from Saccharomyces cerevisiae S288C: a useful set of strains and plasmids for PCR-mediated gene disruption and other applications. *Yeast* **14**, 115-132 (1998).

4. Gonzalez-Huici V*, et al.* DNA bending facilitates the error-free DNA damage tolerance pathway and upholds genome integrity. *EMBO J* **33**, 327-340 (2014).
