# Supplementary material for: The Swr1 chromatin-remodeling complex prevents genome instability induced by replication fork progression defects
Source: Nat Commun. 2018 Sep 11;9:3680. doi: 10.1038/s41467-018-06131-2 (PMC6134005; doi:10.1038/s41467-018-06131-2)
Supplement: Supplementary file 3 — Description of Additional Supplementary Files [file 41467_2018_6131_MOESM3_ESM.pdf]

Description of Additional Supplementary Files

File Name: Supplementary Data 1
Description: Spreadsheet containing all calculated p-values for significance.
